# Long noncoding RNA SNHG16 regulates TLR4-mediated autophagy and NETosis formation in alveolar hemorrhage associated with systemic lupus erythematosus

**DOI:** 10.1186/s12929-023-00969-5

**Published:** 2023-09-12

**Authors:** Yu-Tung Hsieh, Yi-Cheng Chen, Yu-Chi Chou, Pin‑Yu Kuo, Yi-Ting Yen, Hung-Wen Tsai, Chrong-Reen Wang

**Affiliations:** 1 Institute of Basic Medical Sciences, College of Medicine, National Cheng Kung University, Tainan, Taiwan; 2 Department of Internal Medicine, National Cheng Kung University Hospital, College of Medicine, National Cheng Kung University, Tainan, Taiwan; 3 Biomedical Translation Research Center, Academia Sinica, Taipei, Taiwan; 4 Department of Microbiology and Immunology, College of Medicine, National Cheng Kung University, Tainan, Taiwan; 5 Department of Surgery, National Cheng Kung University Hospital, College of Medicine, National Cheng Kung University, Tainan, Taiwan; 6 Department of Pathology, National Cheng Kung University Hospital, College of Medicine, National Cheng Kung University, Tainan, Taiwan

**Keywords:** Alveolar hemorrhage, Systemic lupus erythematosus, LncRNA, SNHG16, TLR4, TRAF6, Autophagy, NETosis

## Abstract

**Background:**

Dysregulated long noncoding RNA (lncRNA) expression with increased apoptosis has been demonstrated in systemic lupus erythematosus (SLE) patients with alveolar hemorrhage (AH). SNHG16, a lncRNA, can enhance pulmonary inflammation by sponging microRNAs, and upregulate toll-like receptor 4 (TLR4) expression via stabilizing its mRNAs. TRAF6, a TLR4 downstream signal transducer, can induce autophagy and NETosis formation. In this study, we investigated whether SNHG16 could regulate TLR4-mediated autophagy and NETosis formation in SLE-associated AH.

**Methods:**

Expression of SNHG16, TLR4 and TRAF6 and cell death processes were examined in lung tissues and peripheral blood (PB) leukocytes from AH patients associated with SLE and other autoimmune diseases, and in the lungs and spleen from a pristane-induced C57BL/6 mouse AH model. SNHG16-overexpressed or -silenced alveolar and myelocytic cells were stimulated with lipopolysaccharide (LPS), a TLR4 agonist, for analyzing autophagy and NETosis, respectively. Pristane-injected mice received the intra-pulmonary delivery of lentivirus (LV)-SNHG16 for overexpression and prophylactic/therapeutic infusion of short hairpin RNA (shRNA) targeting SNHG16 to evaluate the effects on AH. Renal SNHG16 expression was also examined in lupus nephritis (LN) patients and a pristane-induced BALB/c mouse LN model.

**Results:**

Up-regulated SNHG16, TLR4 and TRAF6 expression with increased autophagy and NETosis was demonstrated in the SLE-AH lungs. In such patients, up-regulated SNHG16, TLR4 and TRAF6 expression was found in PB mononuclear cells with increased autophagy and in PB neutrophils with increased NETosis. There were up-regulated TLR4 expression and increased LPS-induced autophagy and NETosis in SNHG16-overexpressed cells, while down-regulated TLR4 expression and decreased LPS-induced autophagy and NETosis in SNHG16-silenced cells. Pristane-injected lung tissues had up-regulated SNHG16, TLR4/TRAF6 levels and increased in situ autophagy and NETosis formation. Intra-pulmonary LV-SNHG16 delivery enhanced AH through up-regulating TLR4/TRAF6 expression with increased cell death processes, while intra-pulmonary prophylactic and early therapeutic sh-SNHG16 delivery suppressed AH by down-regulating TLR4/TRAF6 expression with reduced such processes. In addition, there was decreased renal SNHG16 expression in LN patients and mice.

**Conclusions:**

Our results demonstrate that lncRNA SNHG16 regulates TLR4-mediated autophagy and NETosis formation in the human and mouse AH lungs, and provide a therapeutic potential of intra-pulmonary delivery of shRNA targeting SNHG16 in this SLE-related lethal manifestation.

**Supplementary Information:**

The online version contains supplementary material available at 10.1186/s12929-023-00969-5.

## Background

Systemic lupus erythematosus (SLE) with a loss of immune tolerance to autoantigens, has overproduced autoantibodies, dysregulated cytokines milieu and defective T-cell subpopulations [1, 2]. Its crucial pathogenic mechanism is an imbalance between accelerated cell death and disposal of death-related materials [3]. Immune complexes (IC) formed by pre-existing autoantibodies with released autoantigens from cell death processes can deposit in different organs, leading to visceral inflammation. Lupus nephritis (LN) is the most common cause of disease morbidity with IC depositing in glomerular basement membranes [4]. Alveolar hemorrhage (AH), a fatal respiratory emergency, in SLE is mediated by accumulation of IC in pulmonary capillary walls, causing c with intra-alveolar assembly of red blood cells (RBCs) [5]. AH has an up to 10% occurrence in SLE, and an association with higher disease activity than other clinical manifestations such as LN.

Noncoding RNAs (ncRNAs) can be classified as small ncRNAs and long ncRNAs (lncRNAs) [6]. MicroRNAs (miRNAs), small ncRNAs, have complementary interaction with messenger RNAs (mRNAs) at their target sites in the 3′ untranslated region, i.e., miRNAs recognition element (MRE) [7], regulating the expression of proteins involved in various cell death processes [8–10]. LncRNAs modulate physiological responses through interacting with intra-cellular molecules [11], and function as competing endogenous RNAs (ceRNAs) to de-repress the activity of other transcript RNAs by competing for the same miRNAs [12, 13]. By the interaction of lncRNAs with miRNAs, the regulation of gene expression can be extended to a complicated network-based pattern. Interestingly, a novel therapeutic approach in SLE is to utilize small interfering RNAs (siRNAs) or short hairpin RNAs (shRNAs) to target miRNAs or cytoplasmic lncRNAs, and small-molecule compounds capable of de-stabilizing putative ncRNAs [14]. Our earlier experiments demonstrated that intra-pulmonary delivery of shRNA targeting lincRNA-p21, a pro-apoptotic lncRNA presenting in the cytoplasm, could reduce hemorrhagic frequencies through decreasing apoptosis formation in the SLE-AH lungs [15].

Small nucleolar RNAs with exon sequences, named small nucleolar RNA host gene (SNHG), can present in the cytoplasm to serve as ceRNAs to up-regulate the translation of target mRNAs [16, 17]. Increased SNHG16 expression has been demonstrated in interstitial lung disease and acute pulmonary infection, down-regulating the expression of specific miRNAs to enhance inflammation in the lungs [18–21]. TRAF6, a toll-like receptor 4 (TLR4) downstream signaling molecule, is a potent inducer of various cell death processes [22–24]. Silencing SNHG16 expression can alleviate apoptosis and autophagy induced by lipopolysaccharide (LPS), a TLR4 agonist, in lung cells by sponging particular miRNAs [19, 20, 25, 26]. SNHG16 acts as a ceRNA to upregulate TLR4 mRNA expression via targeting miR-15a/16 [27]. This lncRNA can recruit EIF4A3 to stabilize TLR4 mRNA, while its knockdown and overexpression can reduce and enhance the expression of TLR4, respectively [28]. MiR-146a, a ceRNA target of SNHG16, regulates TLR4-mediated cell death by targeting TLR4 and its downstream TRAF6 [19, 24, 29]. In our previous studies analyzing the SLE-associated AH lungs [24], down-regulated miR-146a expression was shown to enhance apoptosis and neutrophil extracellular traps (NETs) formation through reversing its targeting effects on TAF9b, a p53 co-activator/stabilizer, and TRAF6, a signal transducer activating IL-8 production, respectively. Altogether, these findings raise a possibility that SNHG16 participates in the SLE-associated AH pathogenesis via regulating the TLR4-mediated cell death formation.

In this study, SNHG16, TLR4 and TRAF6 expression and cell death processes including apoptosis, autophagy and NETs formation were examined in lung tissues and peripheral blood (PB) leukocytes from AH patients associated with SLE and other autoimmune diseases as well as in the lungs and spleen from a pristane-induced C57BL/6 AH mouse model. The expression of NEAT1, another lncRNA participating in SLE disease activity and sharing the same target miR-146a with SNHG16 [30], was also evaluated in SLE-AH patients and a mouse model. SNHG16-overexpressed or -silenced LPS-stimulated alveolar and myelocytic cells were analyzed for autophagy and NETs formation, respectively. SNHG16-overexpressed or -silenced alveolar cells were also stimulated with doxorubicin (Dox) to assess their apoptosis formation. Pristane-injected mice received the intra-pulmonary delivery of lentivirus (LV) vectors carrying SNHG16 for overexpression, and prophylactic/therapeutic infusion of shRNA targeting SNHG16 to evaluate their impact on the AH lungs. In addition, SNHG16 expression was examined in renal tissues from LN patients and the kidneys of a pristane-induced BALB/c mouse LN model.

## Materials and methods

### Patients and controls

SLE patients, 55 females and 7 males aged 20 to 62 years (36.2 ± 10.2), fulfilling the American College of Rheumatology revised criteria [31], and their age/sex-matched healthy controls (HC) were enrolled with an approval from the Institutional Review Board of National Cheng Kung University Hospital (No. A-ER-108-455 and No. B-ER-111-400). SLE disease activity index 2000 (SLEDAI-2K) scores were used for assessing their disease activity [32]. LN was diagnosed by histopathological and/or laboratory evidences, while AH was defined as new-onset pulmonary infiltrates, overt drop in hemoglobulin (Hb) and other clinical/laboratory findings [33]. Control groups for AH included age/sex-matched HC, LN (only nephritis) and Nil (neither nephritis nor AH). Blood and urine samples were obtained from SLE patients and HC. Lung specimens were from SLE-associated AH with controls from non-inflammatory pneumothorax (PTX). Renal specimens were from LN with controls from uninvolved parts of renal cell carcinoma. In addition, blood samples and lung specimens were from AH patients related to other autoimmune diseases, including anti-neutrophil cytoplasmic antibody (ANCA)-associated vasculitis (AAV), anti-phospholipid syndrome (APS) and IgA vasculitis (IgAV). A summarized table (Additional file 2: Table S1) shows the clinical profiles and therapeutic modalities of AH in different patient groups including SLE, AAV, APS and IgAV.

### Cell lines

The cell lines used in this study were 293T cells (human embryonic kidney cells, American Type Culture Collection, ATCC, Manassas, VA, USA), MLE-12 cells (mouse alveolar epithelial cells, ATCC) and HL-60 cells (human promyelocytic cells, ATCC). All were cultured with 1 × 10^6^ cells/mL in 3.5 cm dish in a humidified incubator with 5% CO_2_ at 37 °C.

### Cell purification

PB mononuclear cells (PBMCs) were purified by Ficoll-Paque PLUS (GE-Healthcare, Chicago, IL, USA) gradient centrifugation. PB neutrophils (PBNs) were obtained by Polymorphprep™ (AXIS-SHIELD PoC AS, Oslo, Norway) gradient separation for the granulocyte fraction with the removal of RBCs by hypotonic lysis. PB monocytes (PBMs) were isolated from strongly adherent cells by incubating PBMCs in tissue culture-treated dishes for 90 min at 37 °C. The purity of human neutrophils and monocytes was more than 90 and 80% by flow cytometric analysis of surface markers CD11b and CD14, respectively, in this study. Urine sediment cells (USCs) were purified by centrifugation of fresh urine specimens at 3000*g* for 30 min under 4 °C. In addition, CD3-positive T cells, CD19-positive B cells and CD14-positive monocytes in PBMCs as well as miR-146a-overexpressed and SNHG16-silenecd green fluorescence protein (GFP)-positive cells were sorted by a Moflo XDP cell sorter (Beckman Coulter, Mountain View, CA, USA).

Wild-type 8-week-old female C57BL/6 Jackson National Applied Research Laboratories (JNarl) mice were purchased from National Laboratory Animal Center, (Taipei, Taiwan), and housed under specific pathogen-free conditions with free access to food and water on a 12 h/12 h light–dark cycle at the Laboratory Animal Center, College of Medicine, National Cheng Kung University. Mice were housed for 1 week before starting the investigation. Animal experiments were approved by the Institutional Animal Care and Use Committee, National Cheng Kung University (No. 109034 and No. 112067), and performed according to the guidelines. Mice received intraperitoneal injection of 3% thioglycollate medium (Difco, Detroit, MI, USA), followed by phosphate-buffered saline (PBS) injection 24 h later. Lavage fluid was centrifuged for 10 min at 200*g* at room temperature (RT) to collect cell pellets. Neutrophils were further isolated by Percoll gradient solution (Sigma-Aldrich, St. Louis, MO, USA). The purity of mouse neutrophils was up to 95% by flow cytometric analysis of the surface marker Ly-6G in this study [24].

### Generation of LV vectors carrying SNHG16

SNHG16 was generated from mouse lung tissue complementary DNAs (cDNAs) by PCR amplification, and cloned into pLV-SFFV-PGK-Puro (SFFV, Imanis Life Sciences, Rochester, MN, USA) Acc65I/EcoRI sites to obtain SFFV-SNHG16 (9.3K bp, Additional file 1: Fig. S1a). Recombinant LV vectors were produced by transfecting sub-confluent 293T cells with SFFV-SNHG16 or SFFV, a negative control (NC), along with packaging psPAX2 and envelope pMD2.G plasmids under the calcium phosphate precipitation transfection [34]. The SFFV-SNHG16 was chosen for further experiments based on the results of quantitative real time polymerase chain reaction (qRT-PCR) analyses on the transfectants (Additional file 1: Fig. S1a). SFFV-SNHG16 and SFFV vectors were harvested and concentrated by ultracentrifugation with viral titers determined in transduction unit (TU) [34].

### Construction of LV-based shRNA targeting mouse SNHG16

Four shRNA sequences were designed as follows, including three targeting mouse SNHG16 with a NC targeting luciferase, an enzyme catalyzing insect luciferin.

#1, sense 5′-CCTAAGGTTAAGTCGCCCTCG-3′, antisense 5′-CGAGGGCGACTTAACCTTAGG-3′.

#2, sense 5′-GGATTTGGTTGAATATAAA-3′, antisense 5′-TTTATATTCAACCAAATCC-3′.

#3, sense 5′-CACAAATAAACTTTATAAA-3′, antisense 5′-TTTATAAAGTTTATTTGTG-3′.

NC, sense 5′-CCTAAGGTTAAGTCGCCCTCG-3′, antisense 5′-CGAGGGCGACTTAACCTTAGG-3′.

A 1.9 kb stuffer was removed from pLKO.1-puro (National RNAi Core Facility, Academia Sinica, Taipei, Taiwan) by AgeI and EcoRI for cloning shRNA sequences targeting SNHG16 or NC (7.4K bp, Additional file 1: Fig. S1b). To obtain recombinant LV vectors, the created pLKO.1-sh-SNHG16 #1, #2, #3 and -sh-luciferase vectors were transfected into sub-confluent 293T cells, along with packaging/envelope plasmids psPAX2/pMD2.G by the calcium phosphate precipitation to obtain LV-sh-SNHG16 #1, #2, #3 and LV-sh-luciferase. The LV-sh-SNHG16 #3 was chosen for further experiments based on the results of qRT-PCR analyses on the transfectants (Additional file 1: Fig. S1b). LV-sh-SNHG16 or LV-sh-luciferase vectors were harvested and concentrated by ultracentrifugation with the determined viral titers in TU.

### Generation of clustered regularly interspaced short palindromic repeat (CRISPR) -Cas13d LV vector targeting human SNHG16

Guide RNA sequences targeting human SNHG16 were designed with a NC targeting mCherry, a monomeric red fluorescent protein. A 2.7 kp stuffer was removed from pAll-EF1a-CasRx-2A-EGFP, a RNA-targeting CRISPR-Cas13d LV vector (Biomedical Translation Research Center, Academia Sinica) with a GFP domain [24], by *BsmB*I for cloning of guide sequences and NC after an overnight ligation to create CRISPR-CasRX-SNHG16 and -NC, respectively. Sub-confluent 293T cells were transfected with CRISPR-CasRX-SNHG16 or -NC for 48 h at 37 °C. GFP-positive cells were sorted and examined by qRT-PCR analyses. Guide sequences 5ʹ-TTAGAGGAACAATTAGCAGCAGA-3ʹ targeting SNHG16 with the highest silencing efficacy and 5ʹ-CGCCGCCGTCCTCGAAGTTCATC-3′ targeting mCherry as a NC were chosen for further experiments in this study.

### Construction of LV vectors carrying miR‑146a

Recombinant LV vectors with the pre-miRNA expression construct containing pre-miR-146a or pre-miRNA scramble NC (System Biosciences, Palo Alto, CA, USA) were produced by transfecting sub-confluent 293T cells, along with packaging/envelope plasmids psPAX2/pMD2.G under the calcium phosphate precipitation. The LV-miR-146a was chosen for further experiments based on the results of qRT-PCR analyses on the transfectants. LV-miR-146a or LV-miR-scrambled (LV-miR-scr, as a NC) vectors were harvested and concentrated by ultracentrifugation with the determined viral titers in TU.

### Generation of LV-based shRNA targeting miR-146a

The shRNA sequences targeting miR-146a were designed. A 1.9 kb stuffer was removed from pLKO.1-puro by AgeI and EcoRI for cloning shRNA sequences. To obtain recombinant LV vectors, the created pLKO.1-sh-miR-146a vectors were transfected into sub-confluent 293T cells, along with packaging psPAX2 and envelope pMD2.G plasmids by calcium phosphate precipitation to obtain LV-sh-miR-146a. Based on the results of RT-PCR analyses on miR-146a levels in LV-sh-miR-146a-transfected transfectants, the shRNA sequence sense 5ʹ-AGTGTCAGACCTCTGAAATTA-3ʹ, and antisense 5ʹ-TAATTTCAGAGGTCTGACACTTTTTT-3ʹ was chosen for further experiments. LV-sh-luciferase was used as a NC. Stable LV-sh-miR-146- or LV-sh-luciferase-transfected transfectants were created under puromycin selection process in sorted CRISPR-CasRX-SNHG16-transfected HL-60 cells.

### qRT-PCR analyses

Total RNAs from mouse tissues and mouse or human cells were extracted by TRIzol reagent (Invitrogen, Carlsbad, CA, USA). Total RNAs from formalin-fixed, paraffin-embedded human lung or kidney tissues were purified by RNeasy FFPE Kit (Qiagen, Hilden, Germany). RNAs were reverse transcribed into cDNAs by TaqMan Reverse Transcription Reagent Kit (Applied Biosystems, Foster City, CA, USA). cDNAs were used for qPCR by the SYBR qPCR Mix Kit (Thermo Fisher Scientific, Waltham, MA, USA), and the amplification was performed in a RT-PCR system (Applied Biosystem). The primers sequences and melting temperature (Tm) were as follows.

Human SNHG16 (Tm 57 °C): F: 5′-CAGAATGCCATGGTTTCCCC-3′/R: 5′-TGGCAAGAGACTTCCTGAGG-3′.

Mouse SNHG16 (Tm 61 °C): F: 5′-TGACTCGGAAGGGTGCCTGTG-3′/R: 5′-AATCTGCCACTTAGCACACCCCTC-3′.

Human NEAT1 (Tm 59 °C): F: 5′-GCTGGACCTTTCATGTAACGGG-3′/R: 5′-TGAACTCTGCCGGTACAGGGAA-3.

Mouse NEAT1 (Tm 57 °C): F: 5′-TGGAGATTGAAGGCGCAAGT-3′/R: 5′-ACCACAGAAGAGGAAGCACG-3′.

Human TRAF6 (Tm 55 °C): F: 5′-CCTTTGGCAAATGTCATCTGTG-3′/R: 5′-CTCTGCATCTTTTCATGGCAAC-3′.

Mouse TRAF6 (Tm 57 °C): F: 5′-GCAGTGAAAGATGACAGCGTGA-3′/R: 5′-TCCCGTAAAGCCATCAAGCA-3′.

Human TLR4 (Tm 57 °C): F: 5′-AGCCGAAAGGTGATTGTTGT-3′/R: 5′-AGCAGGGTCTTCTCCACCTT-3′.

Mouse TLR4 (Tm 56 °C): F: 5’-GCTTTCACCTCTGCCTTCAC-3’/R: 5’-CACAATAACCTTCCGGCTCT-3’.

Human p53 (Tm 55 °C): F: 5′-CCAGCCAAAGAAGAAACCAC-3′/R: 5′-CTCTCGGAACATCTCGAAGC-3′.

Mouse p53 (Tm 56 °C), F: 5′-TGAACCGCCGACCTATCCTTA-3′/R: 5′-GGCACAAACACGAACCTCAAA-3′.

Human Bax (Tm 56 °C), F: 5′-ACGGCCTCCTCTCCTACTTT-3′/R: 5′-CTCAGCCCATCTTCTTCCAG-3′.

Mouse Bax (Tm 59 °C), F: 5′-AGGATGCGTCCACCAAGAAGCT-3′/R: 5′-TCCGTGTCCACGTCAGCAATCA-3′.

Human LC3 (Tm 55 °C) F: 5′-AGCGTCTCCACACCAATCTC-3′/R: 5′-CAATTTCATCCCGAACGTCT-3′.

Mouse LC3 (Tm 55 °C) F: 5′-GTCCTGGACAAGACCAAGTTCC-3′/R: 5′-CCATTCACCAGGAGGAAGAAGG-3′.

Human Beclin-1 (Tm 56 °C) F:5′-AGGTTGAGAAAGGCGAGACA-3′/R: 5′-GCTTTTGTCCACTGCTCCTC-3′.

Mouse Beclin-1 (Tm 55 °C) F: 5′-GGCCAATAAGATGGGTCTGA-3′/R: 5′-TGCACACAGTCCAGAAAAGC-3′.

Human IL-6 (Tm 53 °C): F: 5′-ACTCACCTCTTCAGAACGAATTG-3′/R: 5′-CATCTTTGGAAGGTTCAGGTTG-3′.

Mouse IL-6 (Tm 55 °C): F: 5′-CTGCAAGAGACTTCCATCCAG-3′/R: 5′-AAGTGGTATAGACAGGTCTGTTGG-3′.

Human IL-8 (Tm 58 °C): F: 5′-GAGAGTGATTGAGAGTGGACCAC-3′/R: 5′-CACAACCCTCTGCACCCAGTTT-3′.

Mouse IL-8 (Tm 57 °C): F: 5′-TGCATGGACAGTCATCCACC-3′/R: 5′-ATGACAGACCACAGAACGGC-3′.

Human interferon-α (IFN-α) (Tm 62 °C): F: 5′-AATCTCTCCTTCCTCCTGTCTGATG-3′/R: 5′-TCTGACAACCTCCCAGGCACA-3′.

Mouse IFN-α (Tm 57 °C): F: 5′-TGTCTGATGCAGCAGGTGG-3′/R: 5′-AAGACAGGGCTCTCCAGAC-3′.

Human MX-1 (Tm 58 °C): F: 5′-GGACTGCGAGGATGATG-3′/R: 5′-CGCCAGCTCATGTGCATCT-3′.

Mouse MX-1 (Tm 60 °C): F: 5′-TGGACATTGCTACCACAGAGGC-3′/R: 5′-TTGCCTTCAGCACCTCTGTCCA-3′.

Human mammalian target of rapamycin (mTOR) (Tm 56 °C): F: 5ʹ-GGCTAGTGGACCAGTGGAAA-3ʹ/R: 5ʹ-CCATTCCAGCCAGTCATCTT-3′.

Human p62 (Tm 57 °C) F: 5′-TGCCCAGACTACGACTTGTG-3′/R: 5′-GTGTCCGTGTTTCACCTTCC-3′.

Human high-mobility group box 1 (HMGB1) (Tm 58 °C): F: 5ʹ-GCGAAGAAACTGGGAGAGATGTG-3ʹ/R: 5ʹ-GCATCAGGCTTTCCTTTAGCTCG-3′.

Mouse ATG5 (Tm 56 °C) F: 5′-GATGGACAGCTGCACACACT-3′/R: 5′-GCTGGGGGACAATGCTAATA-3′.

Human GAPDH (Tm 54 °C): F: 5′-ACTTCAACAGCGACACCCACT-3′/R: 5′-GCCAAATTCGTTGTCATACCAG-3′.

Mouse GAPDH (Tm 56 °C): F: 5′-GTTGTCTCCTGCGACTTCAACA-3′/R: 5′-TTGCTGTAGCCGTATTCATTGTC-3′.

All mRNA levels were normalized to GAPDH by ΔCt method. For analyzing miR-146a and miR-17 levels, total RNAs were reverse transcribed by a TaqMan MicroRNA reverse transcriptase kit (Applied Biosystems) in Smart Cycler (Cepheid, Sunnyvale, CA, USA). Quantitative levels of miR-146a and miR-17 were analyzed with RNU6B small RNA (Applied Biosystems) as an endogenous control [24]. The average levels of human HC cells or control tissues, neutrophils from healthy individuals, mouse cells or control tissues on day 0, and PBMs, mouse neutrophils, MLE-12 cells or HL-60 cells without stimulation were determined as 100%.

### Dox-induced apoptosis in MLE-12 cells

MLE-12 cells were seeded with 1 × 10^6^ cells/mL in 3.5 cm dish in the presence of different concentrations of Dox (TTY Biopharm, Taipei, Taiwan) for 24 h at 37 °C. After stimulation, cells were stained with PE-Annexin V (BD Pharmingen, San Diego, CA, USA) and 7-amino-actinomycin D (BD Pharmingen). Annexin V-positive and 7-amino-actinomycin D-negative cells were as apoptosis, and average apoptotic percentages without stimulation were as apoptotic cell ratio 1.0 [15, 35]. MLE-12 cells were also stained by terminal deoxynucleotidyl transferase dUTP nick end labeling (TUNEL) detection cocktail (Promega, Madison, WI, USA) with cell nuclei counterstained by DAPI (Sigma-Aldrich), and observed under a confocal microscope. After stimulation, these cells were subjected to qRT-PCR analyses for expression of p53 and Bax, or treated by lysis buffer for immunoblot assay with primary antibodies against p53 (Santa Cruz, Santa Cruz, CA, USA) and Bax (Abcam, Cambridge, UK). Their culture supernatants were assessed for HMGB1 concentrations by ELISA (LSBio, Seattle, WA, USA). In addition, PBMCs were stained with PE-Annexin V and 7-amino-actinomycin D with average apoptotic percentages in HC as apoptotic cell ratio 1.0.

### Apoptosis induction in SNHG16-overexpressed and -silenced MLE-12 cells

MLE-12 cells (1 × 10^6^ cells/mL) in 3.5 cm dish were transfected with SFFV-SNHG16/SFFV or sh-SNHG16/sh-luciferase for 48 h at 37 °C in the presence of polybrene, and further incubated with puromycin (2 µg/mL, Sigma-Aldrich) to select successfully transfected stable cells confirmed by qRT-PCR analyses. Stable transfectants, 1 × 10^6^ cells/mL in 3.5 cm dish, were stimulated with 1 µM Dox for 24 h at 37 °C [15]. After stimulation, cells were stained with PE-Annexin V and 7-amino-actinomycin D with average apoptotic percentages in those without stimulation as apoptotic cell ratio 1.0.

### NETs formation in PBNs, HL-60 cells and mouse neutrophils

PBNs (5 × 10^5^ cells/mL) were allowed to adhere to a poly-l-lysine (Sigma-Aldrich) coated 24-well plate in the presence 3 μg/mL LPS (*Pseudomonas aeruginosa* 10, Sigma-Aldrich), for 4 h at 37 °C. HL-60 cells were cultured with 10^6^ cells/mL in 3.5 cm dish in the presence of 1.25% dimethyl sulfoxide (DMSO, Sigma-Aldrich) for 5 days at 37 °C to induce a differentiated status, dHL-60 cells. These cells were cultured in the presence of 500 ng/mL LPS with serum-free X-VIVO 15 medium (Lonza, Basel, Switzerland) for 4 h at 37 °C. Mouse neutrophils (10^6^ cells/mL) were cultured in a 3.5 cm dish for 30 min at 37 °C, while attached neutrophils were incubated under the same condition with different concentrations of HMGB1 (Atlantis Bioscience, Singapore, Republic of Singapore) or 3 μg/mL LPS for 4 h. After culture, aforementioned cells were stained with Sytox Green (Thermo Fisher Scientific) to detect DNAs under a fluorescence microscope. Their morphology was categorized into lobulated neutrophils, de-lobulated neutrophils, diffused NETs and spread NETs [24]. Furthermore, these cells were subjected to qRT-PCR analyses. Their culture supernatants and cells lysates were measured by enzyme‑linked immunosorbent assay (ELISA) for citrullinated histone 3 (CitH3) and protein arginine deiminase 4 (PAD4) concentrations (Cayman, Ann Arbor, MI, USA), respectively.

### NETs formation in SNHG16‑overexpressed and ‑silenced HL‑60 cells

HL-60 cells (10^6^ cells/mL) in 3.5 cm dish were transfected with SFFV or SFFV-SNHG16 for 48 h at 37 °C in the presence of polybrene. These cells were incubated with puromycin to select SNHG16‑overexpressed transfectants. Furthermore, HL-60 cells were transfected with CRISPR-CasRX-NC or -SNHG16 for 48 h at 37 °C in the presence of polybrene, and GFP-positive cells were sorted to obtain SNHG16‑silenced cells confirmed by qRT-PCR analyses. SNHG16‑overexpressed or ‑silenced cells were cultured in the presence of 1.25% DMSO for 5 days to induce dHL-60 cells, and then stimulated with 500 ng/mL LPS with serum-free X-VIVO 15 medium for 4 h at 37 °C. Such cells were stained with Sytox Green to detect DNA morphology under fluorescence microscopy. The culture supernatants and cells lysates were measured by ELISA for CitH3 and PAD4 concentrations, respectively.

### LPS-induced autophagy in MLE-12 cells

MLE-12 cells were seeded with 1 × 10^6^ cells/mL in 3.5 cm dish in the presence of different concentrations of LPS for 4 h at 37 °C. After stimulation, these cells were subjected to qRT-PCR analyses for LC3 and Beclin-1 expression. Alternatively, after overnight stimulation with 50 μg/mL LPS, MLE-12 cells treated by lysis buffer were subjected to immunoblot assay with primary antibodies against LC3 (Cell Signaling, Danvers, MA, USA) and Beclin-1 (Cell Signaling). MLE-12 cells treated with 1 µM rapamycin (Rapa, Sigma-Aldrich) were served as a PC. In addition, PBMs (5 × 10^5^ cells/mL) were cultured in 24-well plate in the presence 50 μg/mL LPS for 4 h at 37 °C, and subjected to qRT-PCR analyses for LC3/Beclin-1 expression.

### Autophagy formation in SNHG16‑overexpressed and ‑silenced MLE-12 cells

MLE-12 cells were transfected with SFFV/SFFV-SNHG16 or sh-luciferase/sh-SNHG16 for 48 h at 37 °C, and incubated with puromycin to select successfully transfected stable cells confirmed by qRT-PCR analyses. SNHG16-overexpressed and -silenced transfectants, 1 × 10^6^ cells/mL in 3.5 cm dish, were stimulated with 50 μg/mL LPS for 4 h at 37 °C. These cells were subjected to qRT-PCR analyses for LC3/Beclin-1 expression after stimulation.

### TLR4 expression in miR‑146a‑overexpressed MLE‑12 cells

MLE-12 cells (1 × 10^6^ cells/mL) in 3.5 cm dish were transfected with LV-miR-146a or LV-miR-scr for 48 h at 37 °C in the presence of polybrene. GFP-positive cells were sorted to obtain miR‑146a‑overexpressed cells confirmed by qRT-PCR analyses. Stable SFFV-SNHG16- and SFFV-transfected transfectants were created by using puromycin selection process in sorted miR‑146a‑overexpressed MLE-12 cells, and were subjected to qRT-PCR analyses for TLR4 expression.

### NETs formation in miR-146a-silenced HL-60 cells

Sorted CasRX-SNHG16-transfected HL-60 cells were transfected with sh-miR-146a or sh-luciferase and underwent puromycin selection to create stable transfectants. Selected transfectants were cultured in the presence of 1.25% DMSO for 5 days to induce dHL-60 cells, and further received 500 ng/mL LPS stimulation with serum-free X-VIVO 15 medium for 4 h at 37 °C. These cells were stained with Sytox Green to detect DNA morphology, and culture supernatants and cells lysates were measured for CitH3 and PAD4 concentrations, respectively.

### Pristane-induced mouse AH or LN model

Female 8-week-old C57BL/6 JNarl mice received intraperitoneal injection of 0.5 mL pristane to induce AH, while their controls were injected with 0.5 mL PBS [15]. They were sacrificed on day 0, 4, 9 and 14 to obtain the lungs and spleen. Their blood samples on day 14 were measured for RBC numbers, Hb levels and hematocrit (Hct) by a blood cell analyzer. Their serum samples on day 14 were examined for anti-RNP levels with a mouse anti-RNP ELISA kit (Alpha Diagnosis, San Antonio, TX, USA).

Female 8-week-old BALB/c JNarl mice from National Laboratory Animal Center, (Taipei, Taiwan), received intraperitoneal injection of 0.5 mL pristane and PBS to induce LN and serve as controls, respectively [35]. They were sacrificed at month 0, 1, 3, 5 and 6 to obtain the kidneys. Urine specimens were collected for measuring proteinuria by test strips (Arkray, Edina, MN, USA) with results determined by a urine chemistry analyzer at month 0, 1, 3, 5 and 6. The serum samples were examined for anti-dsDNA and anti-RNP ELISA kits (Alpha Diagnosis) at month 0, 1, 3, 5 and 6.

### Intra-pulmonary SFFV-SNHG16 or sh-SNHG16 delivery

Mice received SFFV-SNHG16/SFFV or sh-SNHG16/sh-luciferase of 2 × 10^9^ TU/mL by intra-tracheal prophylactic delivery of fluid bolus into the posterior oropharynx above the tracheal entrance on day − 1, followed by intraperitoneal injection of 0.5 mL pristane on day 0. Furthermore, pristane-injected mice received 2 × 10^9^ TU/mL of sh-SNHG16/sh-luciferase by intra-tracheal therapeutic delivery on day 4 or 8. AH was evaluated according to gross and histopathological findings classified into no, partial and complete hemorrhage on day 14 [15, 24].

### Immunoblotting assay

Cell lysates or mouse tissue homogenates were separated by electrophoresis on 10% SDS-PAGE, transferred on PVDF membranes (Merck Millipore, Burlington, MA, USA), blocked in 5% of non-fat dry milk, and incubated with primary antibodies including anti-Bax (1:2000, Abcam), anti-Beclin-1 (1:1000, Cell Signaling), anti-CitH3 (1:1000, Abcam), anti-LC3 (1:1000, Cell Signaling), anti-mTOR (1:1000, Cell Signaling), anti-p53 (1:1000, Santa Cruz), anti-PAD4 (1:1000, Abcam), anti-TLR4 (1:000, Santa Cruz), anti-TRAF6 (1:1000, Santa Cruz), anti-GAPDH (1:5000, Proteintech, Rosemont, IL, USA) or anti-β-actin antibodies (1:5000, Sigma-Aldrich) at 4 °C for 16 h. After washing, the membranes were incubated with HRP-conjugated secondary antibodies (1:10,000, Jackson Immunoresearch, West Grove, PA, USA) at RT for 2 h. Signal expression of protein-antibody complexes was detected by ECL system (Amersham Pharmacia Biotech, Buckinghamshire, UK) and visualized with Biospectrum imaging system (UVP, Upland, CA, USA). Relative protein expression levels were measured by Image J (National Institute of Health, Bethesda, MD, USA).

### Histopathological, TUNEL and immunofluorescence staining

Removed lung tissues were fixed in 10% buffered formalin overnight, and embedded in paraffin. Lung tissues were cut into 5 µm sections, and stained with hematoxylin and eosin (H&E). Paraffin-embedded sections were de-paraffinized in xylene, dehydrated in ethanol and rehydrated in distilled water. To determine glomerulonephritis (GN), mouse renal tissues were analyzed by Periodic acid-Schiff staining [35]. For TUNEL staining, de-paraffinized mouse or human lung sections were treated by proteinase K to reactivate antigens, re-fixed by 4% formaldehyde, incubated with equilibrate buffer, and finally labelled by TUNEL detection cocktail [15]. Cell nuclei were counterstained with DAPI. For detecting the expression of CitH3 or LC3, de-paraffinized mouse or human lung sections were stained with anti-CitH3 or anti-LC3 antibodies, followed by Alexa Fluor 488-conjugated antibodies (Thermo Fisher Scientific), while cell nuclei were counterstained with Hoechst 33258 [24].

Their fluorescence was detected by a confocal microscope. Cells with positive TUNEL, colocalized CitH3 with DNAs or cytoplasmic LC3 were determined by averaging the number from 3 fields of positively stained cells with the highest density in each section.

### Statistical analyses

Data are expressed as the mean ± standard deviation (SD). Numerical data between patients and HC or between different patient groups were analyzed by Mann–Whitney U test. Correlation analysis was performed by Spearman correlation coefficient test. For comparing SNHG16, TLR4 and TRAF6 levels in PBMC or PBN from SLE and HC, and SNHG16, TLR4 and TRAF6 levels in PBMC or PBN from SLE and SLEDAI-2K, multivariable analysis adjusted for age/sex or plus medications were performed by SAS software 9.4 version (SAS Institute Inc, Cary, NC, USA). Hemorrhage frequencies in the lungs between different mouse groups were compared by Fisher’s exact test. Differences in other analyses were determined by Student’s t test. P values less than 0.05 were considered significant in this study with symbols as * for *p* < 0.05, **p < 0.01 and *** for* p* < 0.001.

## Results

### Up-regulated SNHG16, TLR4 and TRAF6 expression with increased apoptosis, autophagy and NETs formation in SLE-AH lung tissues

**Fig. 1:**
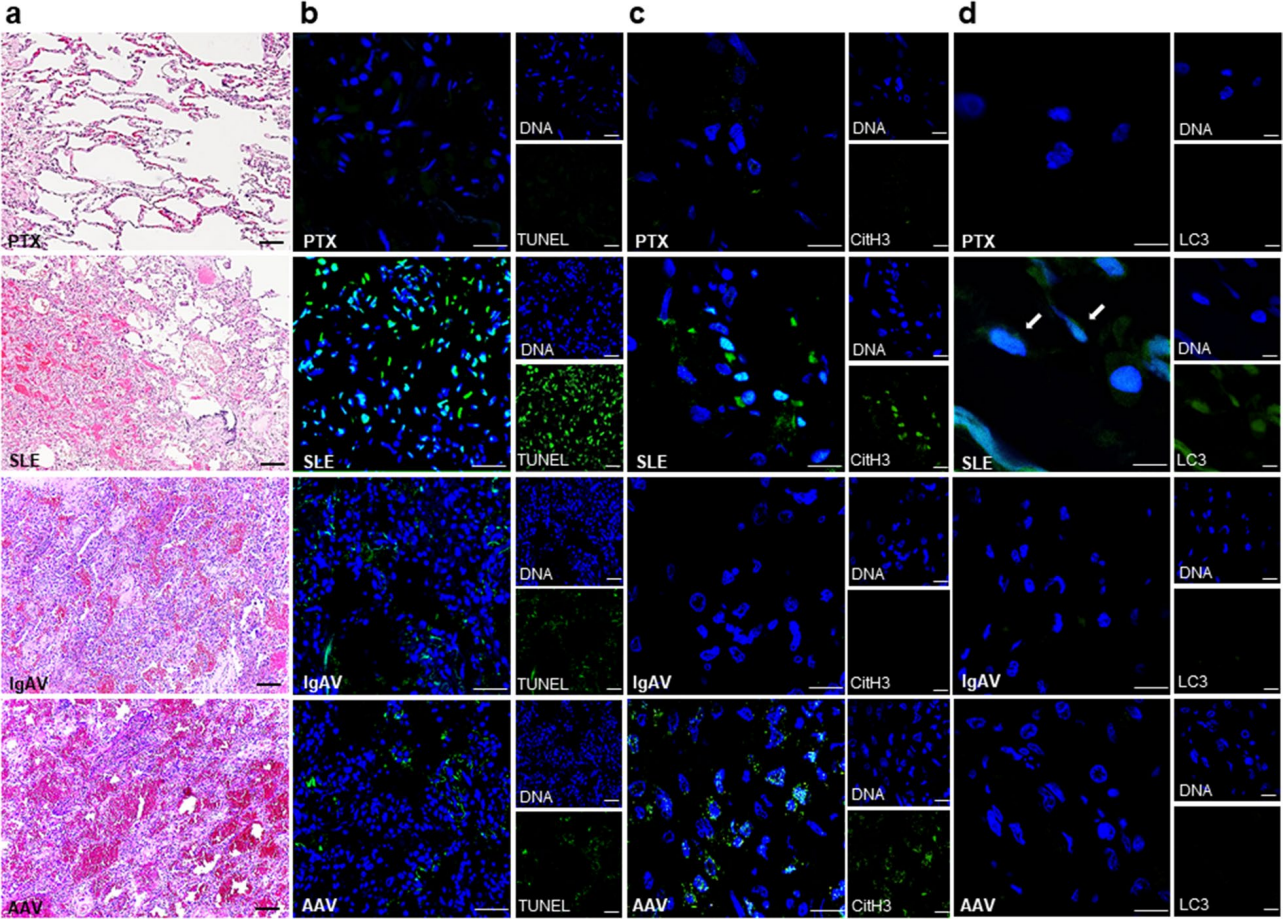
Increased apoptosis, autophagy and NETs formation in SLE-associated AH lung tissues. **a** From top to low, representative histopathology from a PTX control and AH patients from SLE, IgAV and AAV. In PTX, normal alveoli. In SLE, AH with blood in alveoli. In IgAV, AH with interstitial lymphoplasmatic cells and fibrosis. In AAV, AH with fibroblastic plugs in alveoli and alveolar ducts. H&E staining, scale bar = 100 µm and magnification ×100. **b** From top to low, representative TUNEL IF staining (green) from a PTX control and AH patients from SLE, IgAV and AAV. Cell nuclei counterstained with DAPI (blue). Scale bar = 25 µm, magnification ×400. **c** From top to low, representative CitH3 IF staining (green) from a PTX control and AH patients from SLE, IgAV and AAV. Cell nuclei counterstained with Hoechst 33258 (blue). Scale bar = 12.5 µm, magnification ×800.** d** From top to low, representative LC3 IF staining (green) from a PTX control and AH patients from SLE, IgAV and AAV. Arrows pointing cells with positive cytoplasmic LC3 staining. Cell nuclei counterstained with Hoechst 33258 (blue). Scale bar = 10 µm, magnification ×1000 for SLE. Scale bar = 12.5 µm, magnification ×800 for PTX, IgAV and AAV

**Fig. 2:**
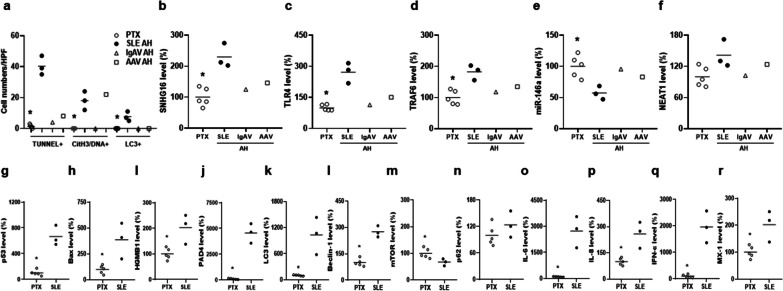
Up-regulated cell death processes and pulmonary expression of SNHG16, TLR4 and TRAF6 in SLE-associated AH lung tissues. **a** Quantification of cell numbers with positive TUNEL, colocalized CitH3 and DNAs, and cytoplasmic LC3 in lung tissues from PTX controls and AH patients from SLE, IgAV and AAV. Expression levels of **b** SNHG16, **c** TLR4, **d** TRAF6, **e** miR-146a and **f** NEAT1 in lung tissues from PTX controls and AH patients from SLE, IgAV and AAV. Expression levels of **g** p53, **h** Bax, **i** HGMB1, **j** PAD4, **k** LC3, **l** Beclin-1, **m** mTOR, **n** p62, **o** IL-6, **p** IL-8, **q** IFN-α and **r** MX-1 in lung tissues from PTX controls and SLE-AH patients. Lung sample numbers, n = 5 for PTX, n = 3 for SLE, n = 1 for IgAV, n = 1 for AAV. Values are mean ± SD. Horizontal lines are mean values. *p < 0.05

Representative H&E-stained lung tissues from a PTX control patient and 3 AH patients with underlying SLE, IgAV or AAV were shown in Fig. 1a. The AH lungs were examined for the expression of apoptosis, autophagy and NETs formation. In Figs. 1b, 2a, higher numbers of TUNEL-positive cells were found in lung tissues from SLE-AH patients than from PTX controls (40.3 ± 6.1 versus 1.4 ± 1.1, *p* = 0.036). In Fig. 1c, 2a, colocalized expression of CitH3 with DNAs, in favor of NETosis, was identified in the SLE-AH lungs but not in the PTX (18.0 ± 6.0 versus 0 ± 0, *p* = 0.018), or IgAV-AH lungs. In particular, binding of ANCA to myeloperoxidase expressed on the membrane of pulmonary neutrophils could be responsible for distinct NETosis in AAV-associated AH lung tissues (Figs. 1c, 2a) [36]. In Fig. 1d, 2a, cytoplasmic LC3-positive cells, suggesting autophagy formation, were identified in SLE-AH but not in PTX (Fig. 2a, 7.7 ± 3.1 versus 0 ± 0, *p* = 0.018), IgAV-AH or AAV-AH lung tissues.

There were up-regulated SNHG16, TLR4 and TRAF6 expression (Fig. 2b–d, *p*  = 0.036), down-regulated miR-146a expression (Fig. 2e, *p* = 0.036), and no differences in NEAT1 expression (Fig. 2f) in SLE-AH lung tissues. Up-regulated levels of cell death-related markers p53, Bax, HMGB1, PAD4, LC3 and Beclin-1 were found in lung tissues from SLE-AH patients (Fig. 2g–l, *p* = 0.036). For other autophagy-related markers, there was down-regulated expression of mTOR (Fig. 2m, *p* = 0.036), an autophagy initiation inhibitor by reducing the activity of autophagy regulatory complexes [23], whereas no differences were found in p62 levels (Fig. 2n). In addition, there were increased levels of cytokines IL-6, IL-8 and IFN-α as well as IFN-inducible gene MX-1 in SLE-AH lung tissues (Fig. 2o–r, *p* = 0.036).

Collectively, these findings suggested up-regulated SNHG16 expression with a synchronously increased TLR4 and TRAF6 levels to enhance apoptosis, autophagy and NETs formation in the SLE-associated AH lungs.

### Up-regulated SNHG16, TLR4 and TRAF6 levels in PBMCs from SLE-AH patients

**Fig. 3:**
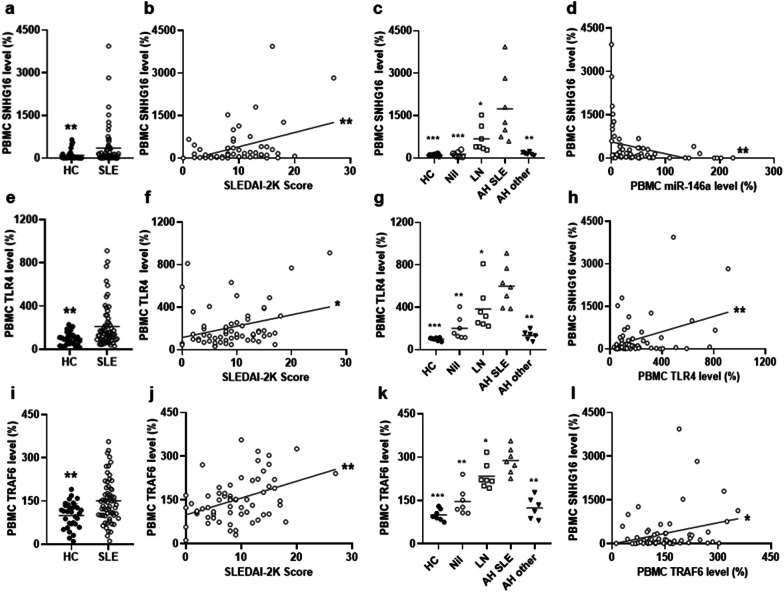
Up-regulated SNHG16, TLR4 and TRAF6 expression in PBMCs from SLE-AH patients. **a** SNHG16, **e** TLR4 and **i** TRAF6 levels in PBMCs from HC and SLE patients. A positive correlation between PBMC **b** SNHG16, **f** TLR4 and **j** TRAF6 levels and SLEDAI-2K activity scores. **c** SNHG16, **g** TLR4 and **k** TRAF6 levels in PBMCs from HC, Nil, LN, SLE-AH and other AH. A positive correlation between SNHG16 and **d** miR-146a, **h** TLR4 or **l** TRAF6 levels in PBMCs from SLE patients. Values are mean ± SD. Horizontal lines are mean values. Patient numbers, n = 62 for SLE, n = 7 for Nil, LN, SLE-AH, n = 6 for other AH. *p < 0.05, **p < 0.01, ***p < 0.001

PBMCs from SLE patients were examined for the expression of SNHG16. Higher levels were found in SLE patients than in HC (Fig. 3a, 352.1 ± 682.4% versus 100.0 ± 174.6%, *p* = 0.006). A positive correlation was found between SNHG16 levels and activity scores (Fig. 3b, r = 0.409, *p* = 0.001). For SNHG16 expression in different patient groups and HC, SLE-AH had higher levels than AH from other autoimmune diseases, LN, Nil or HC (Fig. 3c, *p* = 0.001 for other AH, *p* = 0.038 for LN, *p* < 0.001 for Nil or HC). A negative correlation was found between miR-146a and SNHG16 levels (Fig. 3d, r = − 0.334, *p* = 0.008),

TLR4 levels in PBMCs was higher in SLE patients than in HC (Fig. 3e, 211.5 ± 194.8% versus 100.0 ± 61.3%, *p* = 0.001). A positive correlation was found between TLR4 levels and activity scores (Fig. 3f, r = 0.304, *p* = 0.017). For TLR4 expression, SLE-AH had higher levels than other AH, LN, Nil or HC (Fig. 3g, *p* = 0.001 for other AH, *p* = 0.038 for LN, *p* = 0.002 for Nil, *p* < 0.001 for HC). A positive correlation was found between TLR4 and SNHG16 levels (Fig. 3h, r = 0.384, *p* = 0.002), but not between TLR4 and NEAT1 levels (Additional file 1: Fig. S4a).

The expression of TRAF6, a TLR4 downstream signaling molecule, was higher in SLE patients than in HC (Fig. 3i, 151.1 ± 76.3% versus 100.0 ± 45.3%, *p* = 0.002). A positive correlation was found between TRAF6 levels and activity scores (Fig. 3j, r = 0.365, *p* = 0.004). For TRAF6 expression, SLE-AH had higher levels than other AH, LN, Nil or HC (Fig. 3k,* p* = 0.001 for other AH,* p* = 0.026 for LN, *p* = 0.001 for Nil, *p* < 0.001 for HC). A positive correlation was found between TRAF6 and SNHG16 levels (Fig. 3l, r = 0.272, *p* = 0.033).

Furthermore, there were significant differences by using multivariable analyses adjusted for age and sex for the comparison of SNHG16, TLR4 or TRAF6 levels between SLE and HC, while a significant positive correlation was found by using multivariable analyses adjusted for age, sex and medications for the comparison of SNHG16, TLR4 or TRAF6 levels with activity scores (Additional file 2: Table S2).

In addition, PBMC subpopulations were examined for the expression of SNHG16 in sorted CD3-positive T cells, CD19-positive B cells and CD14-positive monocytes from each healthy individual (Additional file 1: Fig. S2b). In comparison with the average expression levels of SNHG16 in neutrophils from 3 healthy individuals, there were no differences in T cells, whereas lower levels were found in monocytes and B cells (For neutrophils, 100.0 ± 2.0%, for T cells, 85.1 ± 14.0%, for monocytes, 36.3.0 ± 16.3%, *p* = 0.002, for B cells, 30.1 ± 6.4%, *p* = 0.002). We also examined the actual cell number of PB neutrophils, T cells, monocytes and B cells in each healthy individual (Additional file 1: Fig. S2b). There were higher circulating numbers of neutrophils, followed by T cells, monocytes and B cells in each healthy individual.

PBMCs from SLE patients were also examined for the expression of miR-146a. Lower miR-146a levels were found in SLE patients than in HC (Additional file 1: Fig. S3a,* p* < 0.001). A negative correlation was found between miR-146a levels and activity scores (Additional file 1: Fig. S3b, r = − 0.366, *p* = 0.003). For PBMC miR-146a expression, SLE-AH had lower levels than other AH, LN, Nil or HC (Additional file 1: Fig. S3c, *p* = 0.001 for other AH, *p* = 0.026 for LN, *p* < 0.001 for Nil or HC). Furthermore, a negative correlation was found between miR-146a and TLR4 (Additional file 1: Fig. S3d, r = − 0.290, *p* = 0.022), TRAF6 (Additional file 1: Fig. S3e, r = − 0.404, *p* = 0.001), or NEAT1 levels (Additional file 1: Fig. S3f, r = − 0.256, *p* = 0.045).

We further examined the expression of NEAT1, another lncRNA involved in the SLE activity and also a ceRNA targeting miR-146a [30], in PBMCs from SLE patients. Higher levels were found in SLE patients than in HC (Additional file 1: Fig. S4b, 253.8 ± 428.8% versus 100.0 ± 96.9%, *p* = 0.011). A positive correlation was found between NEAT1 levels and activity scores (Additional file 1: Fig. S4c, r = 0.306, *p* = 0.016). For NEAT1 expression in different patient groups and HC, AH had higher levels than HC (Additional file 1: Fig. S4d, *p* = 0.038), whereas no differences were found between SLE-AH and other AH, LN or Nil. Although both lncRNAs had up-regulated expression in SLE patients and acted as ceRNAs targeting miR-146a, SNHG16 rather than NEAT1 appeared to be involved in the pathogenesis of AH manifestation.

Taken together, these results implicated that up-regulated SNHG16 levels with a concurrent increase in the expression of TLR4 and TRAF6 in PBMCs can participate in SLE activity, resulting in the AH manifestation.

### Increased cell death processes in PB leukocytes from SLE-AH patients

In SLE, accelerated apoptosis has been identified in PB lymphocytes and monocytes [3], while increased p53 levels and cell apoptosis in circulating lymphocytes are correlated with the disease activity [35]. The excessive activation of autophagy leads to autophagic cell death, and the expression of autophagy flux-related molecules LC3 and Beclin-1 has been found to be increased in PBMCs from SLE patients [3, 37]. There are overactivated neutrophils with increased NETs formation in PMA-stimulated PBNs from SLE patients [3, 24].

SLE patients had higher p53 levels (Additional file 1: Fig. S5a) and apoptotic cell ratios (Additional file 1: Fig. S5b) in PBMCs than those from HC (For p53, *p* = 0.004; for apoptotic cell, *p* = 0.012). Furthermore, SLE-AH had higher p53 levels (Additional file 1: Fig. S5a) and apoptotic cell ratios (Additional file 1: Fig. S5b) than those from LN, Nil or HC (For p53, *p* = 0.038 for LN, *p* = 0.004 for Nil, *p* < 0.001 for HC; for apoptotic cell, *p* = 0.036 for LN, Nil or HC).

**Fig. 4:**
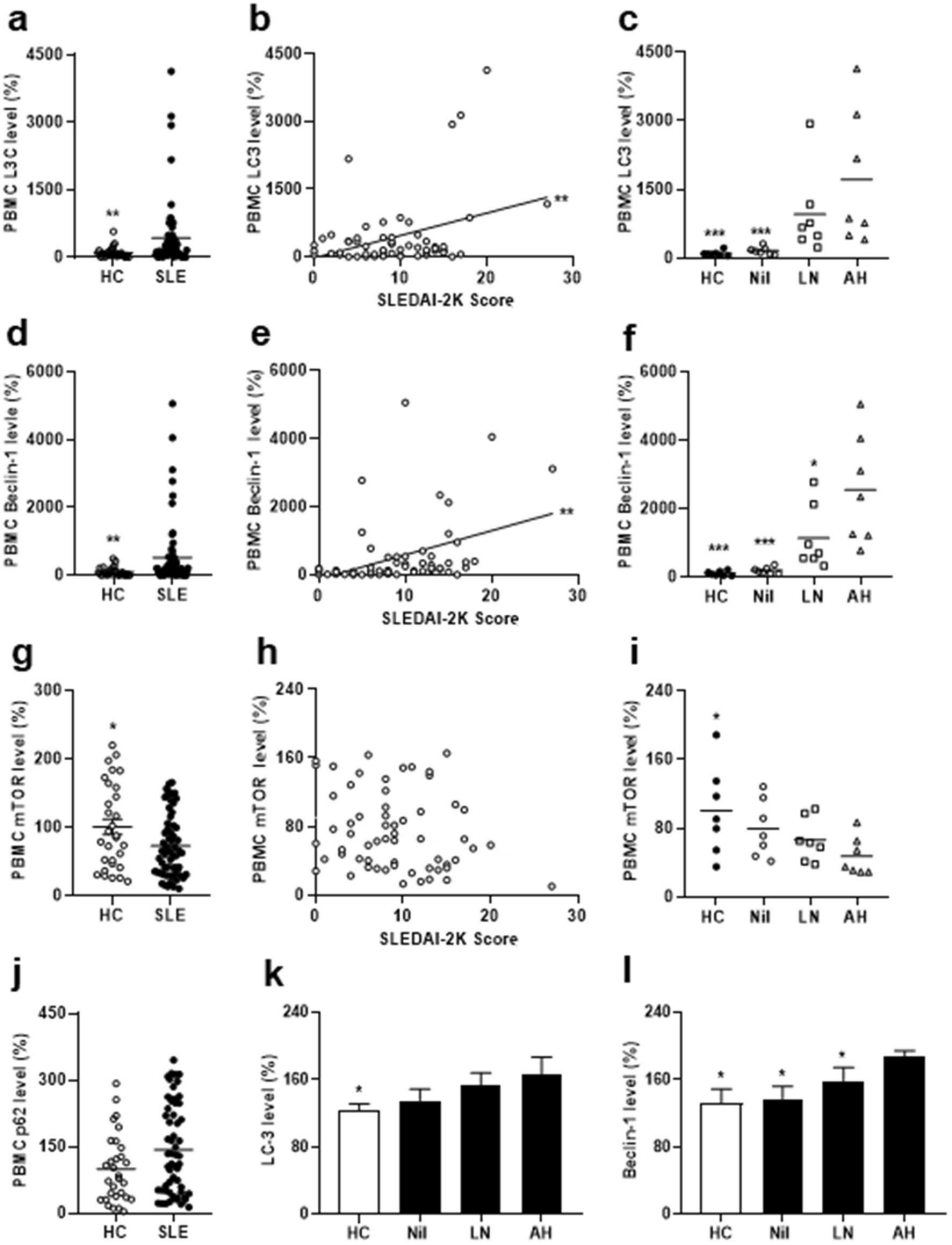
Increased autophagy formation in PBMCs from SLE-AH patients. **a** LC3, **d** Beclin-1, **g** mTOR and **j** p62 levels in PBMCs from SLE patients and HC. A positive correlation between **b** LC3 or **e** Beclin-1 levels in PBMCs from SLE patients and SLEDAI-2K activity scores. No correlation between** h** mTOR levels in PBMCs from SLE patients and SLEDAI-2K activity scores. **c** LC3, **f** Beclin-1 and **i** mTOR levels in PBMCs from HC, Nil, LN and AH patients. **k** LC3 and **l** Beclin-1 levels in LPS-stimulated PBMs from HC, Nil, LN and AH patients. Values are mean ± SD. Horizontal lines are mean values. Patient numbers, PBMCs, n = 62 for SLE, n = 7 for Nil, LN, AH. Patient numbers, PBMs, n = 5 for Nil, LN, n = 3 for AH. *p < 0.05, **p < 0.01, ***p < 0.001

There were higher LC3 and Beclin-1 levels and lower mTOR levels in PBMCs from SLE patients than those from HC (Fig. 4a, for LC3, *p* = 0.002, Fig. 4d, for Beclin-1, *p* = 0.004, Fig. 4g, for mTOR, *p* = 0.047), while LC3 and Beclin-1 levels were positively correlated with activity scores (Fig. 4b, for LC3, r = 0.359, *p* = 0.004; Fig. 4e, for Beclin-1, r = 0.399, *p* = 0.001). No differences in p62 levels were found between SLE patients and HC (Fig. 4j). For LC3, AH had higher levels than Nil or HC (Fig. 4b,* p* < 0.001 for Nil or HC). For Beclin-1, AH had higher levels than LN, Nil or HC (Fig. 4c,* p* = 0.038 for LN, *p* < 0.001 for Nil or HC). For mTOR, AH only had lower levels than HC (Fig. 4i,* p* = 0.018). In LPS-stimulated PBMs, AH only had higher LC3 levels than HC but not other patient groups (Fig. 4k, *p* = 0.036), whereas AH had higher Beclin-1 levels than LN, Nil or HC (Fig. 4l, *p* = 0.036 for LN, Nil or HC). In addition, we performed immunoblotting assay to examine the protein expression of LC3, Beclin-1 and mTOR in PBMCs from SLE patients and HC. SLE patients had higher LC3-II and Beclin-1 but lower mTOR expression than those from HC (Additional file 1: Fig. S6a, representative immunoblot assay for Beclin-1, LC and mTOR in PBMCs from SLE patients and HC; Fig. S6b, *p* = 0.019 for LC-II, *p* = 0.030 for Becline-1,* p* = 0.043 for mTOR).

**Fig. 5:**
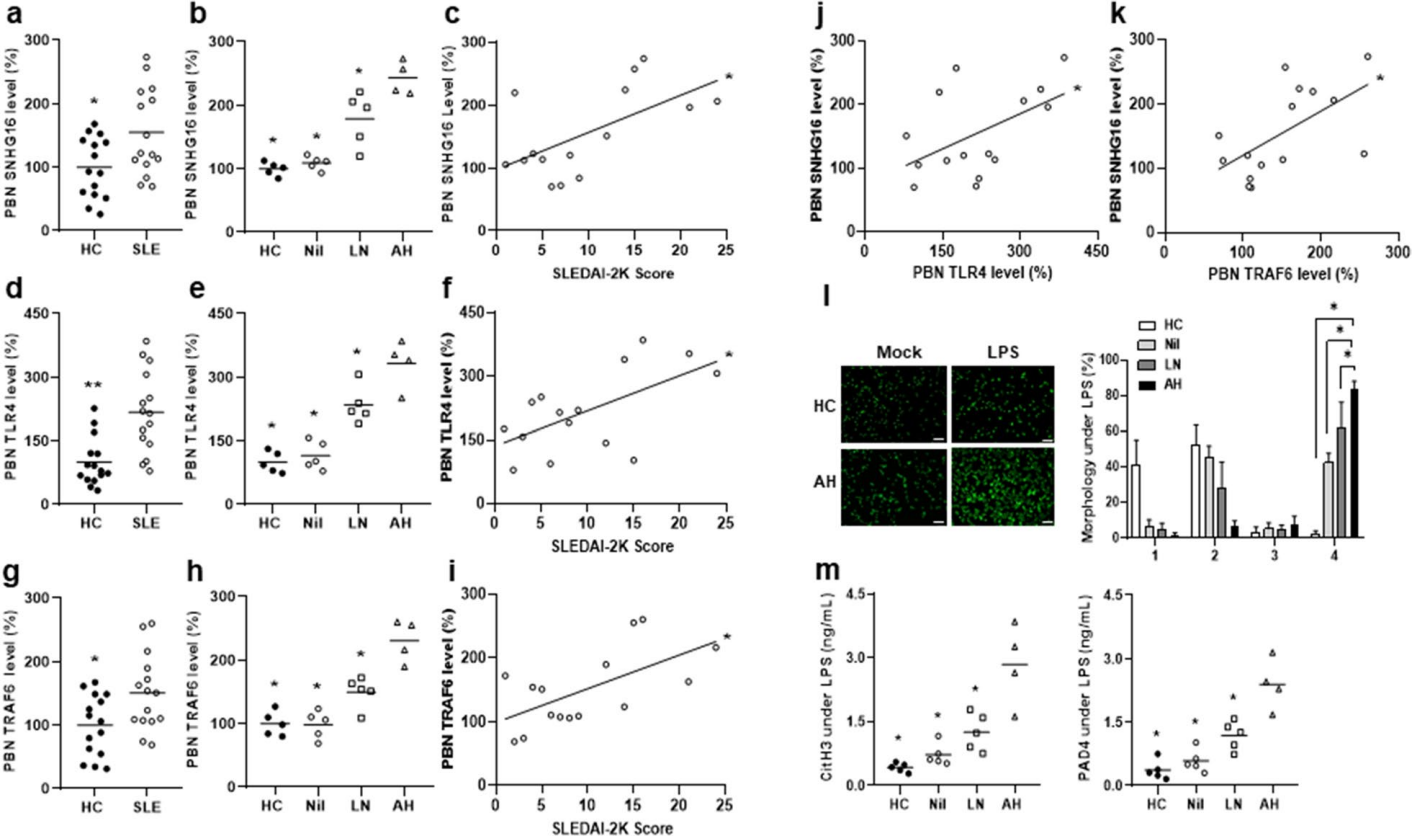
Up-regulated SNHG16, TLR4 and TRAF6 expression and increased NETs formation in PBNs from SLE-AH patients. **a** SNHG16, **d** TLR4 and **g** TRAF6 levels in PBNs from SLE patients and HC. **b** SNHG16, **e** TLR4 and **h** TRAF6 levels in PBNs from HC, Nil, LN and AH patients. A positive correlation between **c** SNHG16, **f** TLR4 and **i** TRAF6 levels in PBNs from SLE patients and SLEDAI-2K activity scores. A positive correlation between SNHG16 and **j** TLR4 or **k** TRAF6 levels in PBNs from SLE patients. PBNs from HC, Nil, LN and AH patients stimulated with LPS to detect DNAs morphology and measure CitH3/PAD4 levels. **l**, Left, representative photographs from a HC and an AH patient. Scale bar = 50 µm, magnification ×200. Right, quantification of NETs formation in HC, Nil, LN and AH patients. **m** Quantification of CitH3 (left) and PAD4 (right) levels in HC, Nil, LN and AH patients. Values are mean ± SD. Horizontal lines are mean values. Patient numbers, n = 15 for SLE, n = 5 for Nil, n = 5 for LN, n = 4 for AH. *p < 0.05, **p < 0.01

Up-regulated expression of SNHG16, TLR4 and TRAF6, but not NEAT1, was identified in PBNs from SLE patients in comparison with those from HC (Fig. 5a, d, g, Additional file 1: S4e, for SNHG16, *p* = 0.045, for TLR4, *p* = 0.002, for TRAF6, *p* = 0.026). A positive correlation was found between activity scores and SNHG16 (Fig. 5c), TLR4 (Fig. 5f) or TRAF6 levels (Fig. 5i), but not NEAT1 levels in PBNs (Additional file 1: Fig. S4f) (For SNHG16, r = 0.566, *p* = 0.028; for TLR4, r = 0.588, *p* = 0.021; for TRAF6, r = 0.617, *p* = 0.014). A positive correlation was found between SNHG16 and TLR4 levels (Fig. 5j, r = 0.518, *p* = 0.048), or TRAF6 levels (Fig. 5k, r = 0.558, *p* = 0.031). For SNHG16, TLR4 or TRAF6 expression in PBNs, AH had higher levels than LN, Nil or HC (Fig. 5b, 5e, for SNHG16 or TLR4,* p* = 0.032 for LN, *p* = 0.016 for Nil or HC; Fig. 5h, for TRAF6, *p* = 0.016 for LN, Nil or HC). For NEAT1 PBN expression, AH only had higher levels than HC (Additional file 1: Fig. S4g, *p* = 0.032). In addition, there was lower miR-146a expression in PBNs from SLE patients than those from HC (Additional file 1: Fig. S3g, *p* = 0.012), while a negative correlation was found between PBN miR-146a and activity scores (Additional file 1: Fig. S3h, r = − 0.539, *p* = 0.038). AH had lower PBN miR-146a levels than LN, Nil or HC (Additional file 1: Fig. S3i,* p* = 0.032 for LN, *p* = 0.016 for Nil or HC).

In addition, there were significant differences by using multivariable analyses adjusted for age and sex for the comparison of SNHG16, TLR4 or TRAF6 levels between SLE and HC, while a significant positive correlation was found by using multivariable analyses adjusted for age, sex and medications for the comparison of SNHG16, TLR4 or TRAF6 levels with activity scores (Additional file 2: Table S3).

Furthermore, purified PBNs were stimulated with LPS to induce NETosis by observing DNA morphology with NETs formation and measuring CitH3 and PAD4 levels. For spread NETs formation, AH had higher percentages than LN, Nil or HC (Fig. 5l, *p* = 0.016 for LN, Nil or HC). For CitH3 or PAD4 production, AH had higher levels than LN, Nil or HC (Fig. 5m, for CitH3, *p* = 0.032 for LN, *p* = 0.016 for Nil or HC; for PAD4, *p* = 0.016 for LN, Nil or HC).

Altogether, increased cell death processes including apoptosis, autophagy and NETosis were found in PB leukocytes from SLE patients, particularly in those with the AH manifestation.

### Increased Dox-induced apoptosis in SNHG16-overexpressed and reduced apoptosis in SNHG16-silenced MLE-12 cells

Since SLE-AH patients had upregulated SNHG16 expression, increased pro-inflammatory cytokine levels and increased apoptosis formation in their lung tissues, we further investigated whether, in alveolar cells, the presence of pro-inflammatory cytokine could regulate the expression of SNHG16, and the apoptotic status could be altered by overexpressing or silencing the expression of SNHG16. SNHG16 levels in the cultures of MLE-12 cells were upregulated in the presence of exogenous IL-6 with a dose-dependent manner (Additional file 1: Fig. S7a, for 31.3 ng/mL, *p* = 0.034, for 62.5 ng/mL, *p* = 0.032, for 125 ng/mL, *p* = 0.007). Furthermore, in Dox-stimulated MLE-12 cells, there were increased TUNEL-positive apoptotic cell percentages and a dose-dependent increase in apoptotic cell ratios (Additional file 1: Fig. S7b), concentrations of HMGB1, a damage-associated molecular pattern (DAMP) molecule released by apoptotic cells [24] (Additional file 1: Fig. S7c), and levels of SNHG16, TRAF6, p53 and Bax (Additional file 1: Fig. S7d, S7e). In addition, we examined the expression of miR-17 and miR-146a, ceRNA targets of SNHG16 [38], in SNHG16-overexpressed and -silenced MLE-12 cells (Additional file 1: Fig. S7f). Furthermore, SNHG16-overexpressed cells had up-regulated p53, Bax levels and apoptotic cell ratios (Additional file 1: Fig. S7g, for p53 or Bax, *p* < 0.001, for apoptotic ratio, *p* = 0.005), whereas SNHG16-silenced cells had down-regulated p53, Bax levels and apoptotic cell ratios (Additional file 1: Fig. S7h, for p53 or Bax,* p* < 0.001, for apoptotic ratio, *p* = 0.006).

As a whole, these results suggested that modulating SNHG16 expression could control the Dox-induced-apoptosis via damaging DNAs to trigger a p53-dependent process in alveolar cells.

### Increased LPS-induced autophagy in SNHG16-overexpressed and decreased autophagy in SNHG16-silenced MLE-12 cells

**Fig. 6:**
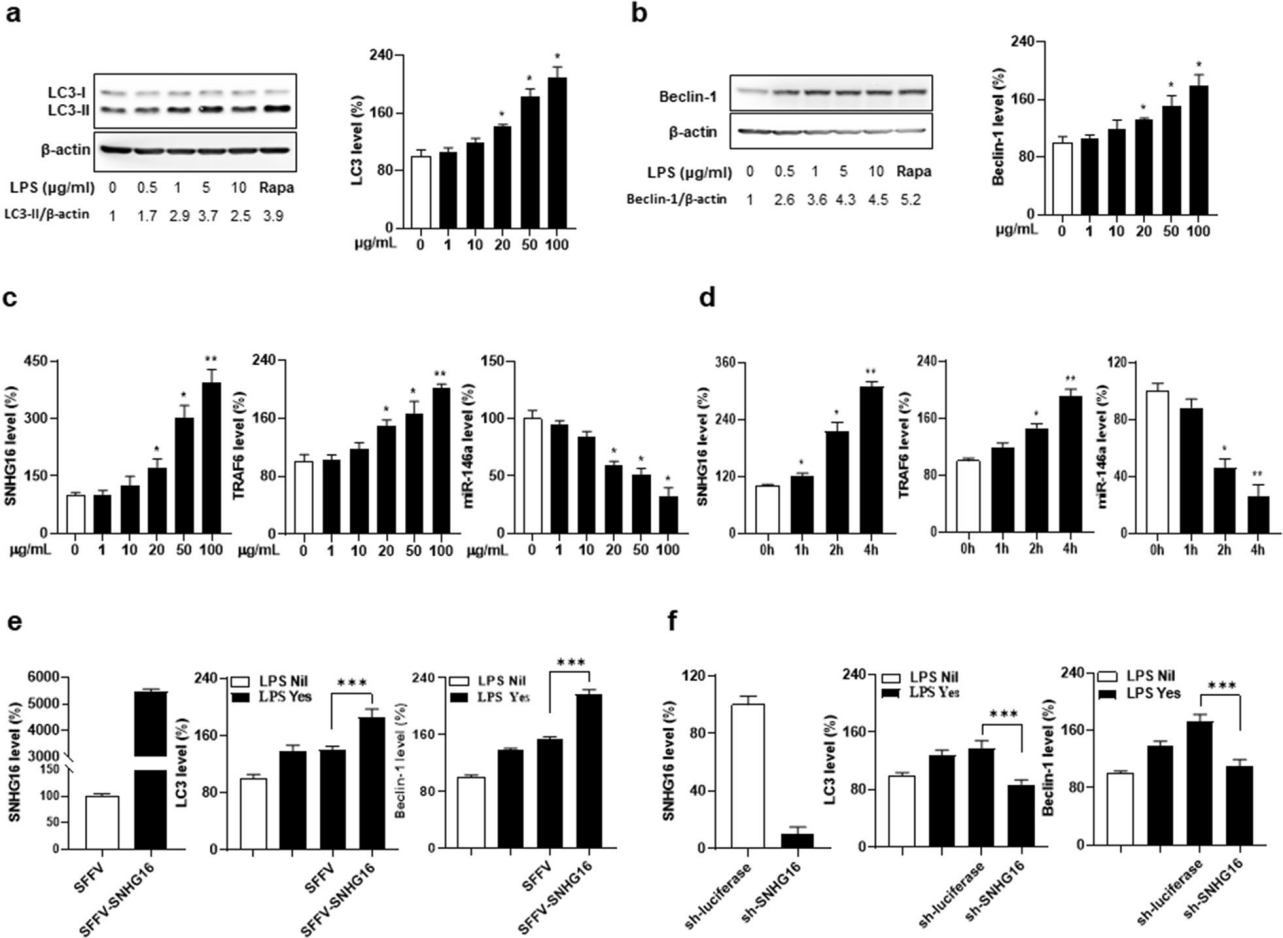
Autophagy formation in LPS-stimulated MLE-12 cells regulated by SNHG16. **a** LC3 and **b** Beclin-1 levels in LPS-stimulated MLE-12 cells. Left, immunoblot assay. Right, qRT-PCR analysis. MLE-12 cells treated with 1 µM rapamycin (Rapa) as a PC. SNHG16, miR-146a and TRAF6 levels in MLE-12 cells stimulated with **c** different LPS concentrations for 4 h and with **d** 50 µg/mL LPS for different times. **e** SNHG16 levels (left) in SNHG16-overexpressed MLE-12 transfectants. LC3 (middle) and Beclin-1 (right) levels in SNHG16-overexpressed transfectants stimulated with 50 µg/mL LPS for 4 h. **f** SNHG16 levels (left) in SNHG16-silenced MLE-12 transfectants. LC3 (middle) and Beclin-1 (right) levels in SNHG16-silenced transfectants stimulated with 50 µg/mL LPS for 4 h. Values mean ± SD. Results in **a**–**d** were representative of 3 independent experiments, and in **e** and **f** were representative of 2 independent experiments with similar findings. *p < 0.05, **p < 0.01, ***p < 0.001

Based on the findings of up-regulated LC3/Beclin-1 levels and increased autophagy formation in PB leukocytes and lung tissues from SLE-AH patients, we further modulated the expression of SNHG16 in alveolar cells to examine its influence on the autophagy formation. In LPS-stimulated MLE-12 cells, by using immunoblot and qRT-PCR analyses, there was a dose-dependent increase in LC3-II and LC3 (Fig. 6a) and in Beclin-1 expression (Fig. 6b). In addition, a dose/time-dependent up-regulated SNHG16 and TRAF6 expression and down-regulated miR-146a levels were identified in such cells (Fig. 6c, 6d). Furthermore, SNHG16-overexpressed cells had increased LC3 and Beclin-1 levels (Fig. 6e, *p* < 0.001), whereas SNHG16-silenced cells had decreased LC3 and Beclin-1 levels (Fig. 6f, *p* < 0.001). In addition, SNHG16-overexpressed and SNHG16-silenced cells had up-regulated and down-regulated TLR4 expression, respectively, by qRT-PCR analysis and immunoblot assay (Additional file 1: Fig. S8a, TLR4 mRNA, for overexpressed cells, *p* = 0.002, for silenced cells, *p* = 0.001; Fig. S8b, representative TLR4 immunoblot assay). These findings indicated that SNHG16 expression could regulate a TLR4/TRAF6 axis-mediated autophagy formation in alveolar cells.

### TLR4-mediated NETs formation in mouse neutrophils by LPS or HMGB1 stimulation

In PBNs from SLE-AH patients, there was up-regulated SNHG16 expression, while LPS stimulation could enhance NETosis as shown by increased spread NETs formation and CitH3/PAD4 production. On day 4, 9 and 14 after the pristane induction, neutrophils were isolated 24 h later from thioglycolate-injected mice. There was up-regulated expression of SNHG16, TLR4 and TRAF6 as well as down-regulated miR-146a levels since day 4 (Additional file 1: Fig. S9a, on day 4, for SNHG16, *p* < 0.001, for TLR4, *p* = 0.002, for TRAF6, *p* = 0.034, for miR-146a, *p* = 0.004), but no differences in the NEAT1 expression (Additional file 1: Fig. S4h).

Neutrophils from naïve mice were stimulated with IL-6, LPS or HMGB1 in the cell culture. SNHG16 levels were upregulated under the stimulation of IL-6 in a dose-dependent manner (Additional file 1: Fig. S9b, for 62.5 ng/mL, *p* = 0.043, for 125 ng/mL, *p* = 0.018, for 250 ng/mL, *p* = 0.019). There were increased diffused/spread NETs morphology and CitH3 production, favoring NETs formation, with up-regulated SNHG16 and TRAF6 as well as down-regulated miR-146a expression in HMGB1-stimulated neutrophils in a dose-dependent manner (Additional file 1: Fig. S9c, HMGB1 300 ng/mL, for morphology, *p* < 0.001, for CitH3, *p* = 0.035, for SNHG16, *p* = 0.040, for TRAF6, *p* = 0.025, for miR-146a, *p* = 0.036; LPS 3 μg/mL, for morphology, *p* < 0.001, for CitH3, *p* = 0.005, for SNHG16, *p* = 0.003, for TRAF6, *p* = 0.031, for miR-146a, *p* = 0.006).

### Increased LPS-induced NETosis in SNHG16-overexpressed and reduced NETosis in SNHG16-silenced dHL-60 cells

**Fig. 7:**
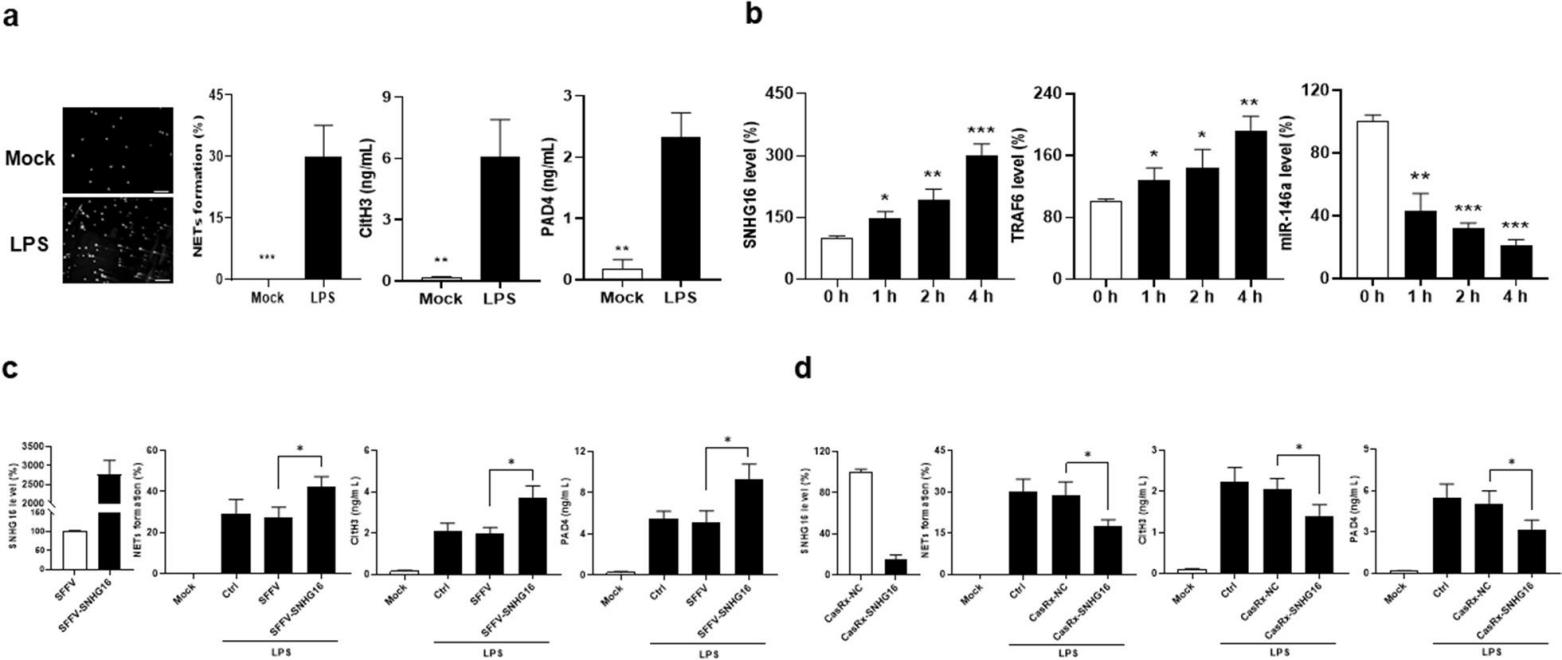
NETs formation in LPS-stimulated dHL-60 cells regulated by SNHG16. **a** LPS-stimulated dHL-60 cells to detect DNAs morphology and measure CitH3/PAD4 levels. Left, representative photographs from mock and LPS stimulation. Scale bar = 60 µm, magnification ×200. Middle left, quantification of diffused/spread NETs morphology percentage. Middle right, CitH3 levels. Right, PAD4 levels. **b** SNHG16, TRAF6 and miR-146a levels in dHL-60 cells stimulated with 500 ng/mL LPS for different times. **c** Left, SNHG16 levels in SNHG16-overexpressed HL-60 transfectants. Middle left diffused/spread NETs morphology quantification. Middle right, CitH3 levels. Right, PAD4 levels in SNHG16-overexpressed dHL-60 transfectants stimulated with 50 µg/mL LPS for 4 h. **d** Left, SNHG16 levels in SNHG16-silenced HL-60 transfectants Middle left, diffused/spread NETs morphology quantification. Middle right, CitH3 levels. Right, PAD4 levels in SNHG16-silenced dHL-60 transfectants stimulated with 500 ng/mL LPS for 4 h. Values are mean ± SD. Results in **a** and **b** were representative of 3 independent experiments, and in **c** and **d** were representative of 2 independent experiments with similar findings. *p < 0.05, **p < 0.01, ***p < 0.001

Differentiated HL-60 cells were stimulated with LPS to induce NETosis as shown by increased diffused/spread NETs morphology and CitH3/PAD4 production (Fig. 7a, for morphology, *p* < 0.001, for CitH3, *p* = 0.005, for PAD4, *p* = 0.001). There were a time-dependent up-regulation of SNHG16 and TRAF6 expression as well as down-regulated miR-146a levels (Fig. 7b, at 1 h, for SNHG16, *p* = 0.010, for TRAF6, *p* = 0.042, for miR-146a, *p* = 0.001). SNHG16-overexpressed dHL-60 cells had higher diffused/spread NETs percentages and CitH3/PAD4 levels (Fig. 7c, for morphology, *p* = 0.022, for CitH3, *p* = 0.011, for PAD4, *p* = 0.016), whereas SNHG16-silenced dHL-60 cells had lower diffused/spread NETs percentages and CitH3/PAD4 levels (Fig. 7d, for morphology, *p* = 0.024, for CitH3, *p* = 0.041, for PAD4, *p* = 0.047).

SNHG16-overexpressed and SNHG16-silenced HL-60 cells had up-regulated and down-regulated TLR4 expression, respectively (Additional file 1: Fig. S10a, TLR4 mRNA, for overexpressed cells, *p* = 0.016, for silenced cells, *p* = 0.001; Fig. S10b, representative TLR4 immunoblot assay). In addition, expression of miR-17 and miR-146a, ceRNA targets of SNHG16 [38], was shown in SNHG16-overexpressed and SNHG16-silenced HL-60 cells (Additional file 1: Fig. S10c). Furthermore, miR-146a expression in SNHG16-silenced HL-60 cells were silenced to elucidate the putative mechanism that up-regulated miR-146a expression is involved in decreased NETosis in LPS-stimulated SNHG16-silenced dHL-60 cells. Sorted CasRX-SNHG16-transfected HL-60 cells were transfected with sh-miR-146a or sh-luciferase to create stable transfectants. After stimulating DMSO-induced dHL-60 cells with LPS, there were higher NETs percentages and CitH3/PAD4 levels in sh-miR-146a-transfected than sh-luciferase-transfected cells (Additional file 1: Fig. S10d, for NETs morphology, *p* = 0.043, for CitH3, *p* = 0.030, for PAD4, *p* = 0.029), implicating that miR-146a expression participates in the regulation of TLR4-mediated NETs formation by SNHG16.

In sum, our experimental data revealed that, in myelocytic cells, a TLR4/TRAF6 axis-mediated NETs formation could be regulated by SNHG16.

### Up-regulated pulmonary SNHG16, TLR4 and TRAF6 expression with increased apoptosis, autophagy and NETs formation in a pristane-induced mouse AH model

**Fig. 8:**
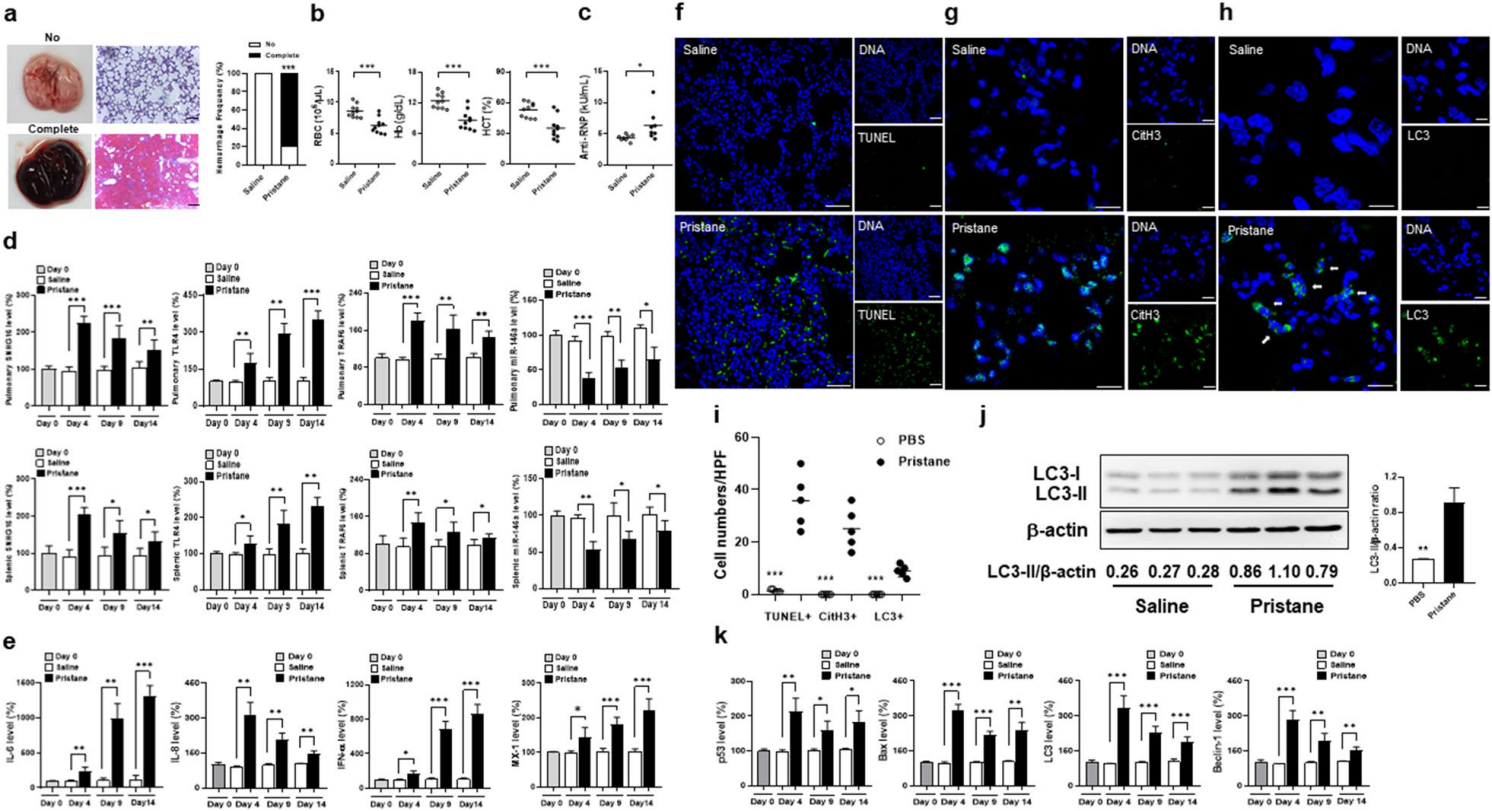
Up-regulated SNHG16, TLR4 and TRAF6 expression with increased apoptosis, autophagy and NETs formation in the mouse AH lungs. **a** Left, representative gross and histopathological photographs in the mouse lungs with no and complete hemorrhage. Right, hemorrhagic frequencies in saline- and pristane-injected mice on day 14. Scale bar = 100 µm, magnification ×100. **b** RBC numbers, Hb levels and Hct on day 14 in saline- and pristane-injected mice. **c** Anti-RNP titers on day 14 in serum samples from saline- and pristane-injected mice. **d** Pulmonary (top) and splenic (low) SNHG16, TLR4, TRAF6 and miR-146a levels on day 0, 4, 9 and 14 from saline- and pristane-injected mice. **e** IL-6, IL-8, IFN-α and MX-1 pulmonary levels on day 0, 4, 9 and 14 from saline- and pristane-injected mice. **f** Representative TUNEL IF staining (green) with cell nuclei counterstained with DAPI (blue). Scale bar = 25 µm, magnification ×400. **g** Representative CitH3 IF staining (green) with cell nuclei counterstained with Hoechst 33258 (blue). Scale bar = 12.5 µm, magnification ×800. **h** Representative LC3 IF staining (green) with cell nuclei counterstained with Hoechst 33258 (blue). Scale bar = 10 µm, magnification ×1000. Arrows pointing cells with positive cytoplasmic LC3 staining. **i** Quantification of cell numbers with positive TUNEL, colocalized CitH3/DNAs, and cytoplasmic LC3 in lung tissues. **j** Immunoblot assay (left) with signal intensity quantitation analysis (right) of pulmonary LC3-II expression from saline- and pristane-injected mice. **k** Pulmonary levels of p53, Bax, LC3 and Beclin-1 on day 0, 4, 9 and 14 from saline- and pristane-injected mice. Values are mean ± SD. Horizontal lines are mean values. Mouse numbers per group, 10 in **a**, **b**, 8 in **c**, 5 in **d**, **i**, 4 in **e**, **k**, 3 in** j**. All results in figure were representative of 2 independent experiments with similar findings. *p < 0.05, **p < 0.01, ***p < 0.001

Figure 8 demonstrates an AH mouse model with complete hemorrhage in 80% of pristane-injected mice and no hemorrhage in PBS-injected controls (Fig. 8a, *p* < 0.001). Lower RBC numbers, Hb levels and Hct were identified in pristane- than PBS-injected mice (Fig. 8b, *p* < 0.001). Higher levels of anti-RNP antibody, favoring an IgM isotype, were found in pristane- than PBS-injected mice on day 14 (Fig. 8c, *p* = 0.038). Up-regulated pulmonary and splenic SNHG16 levels were identified in pristane-injected mice since day 4 (Fig. 8d, day 4, *p* < 0.001), while upregulated TLR4 and TRAF6 expression and down-regulated miR-146a levels were also found in pristane-injected mice (Fig. 8d, day 4, for pulmonary TLR4, *p* = 0.006, TRAF6 and miR-146a, *p* < 0.001, for splenic TLR4, *p* = 0.043, TRAF6, *p* = 0.004, miR-146a, *p* = 0.005). There were no differences between pulmonary and splenic NEAT1 levels in pristane- and PBS-injected mice (Additional file 1: Fig. S4i, S4j). In addition, upregulated expression of IL-6, IL-8, IFN-α and MX-1 was found in pristane-injected mice since day 4 (Fig. 8e, day 4, for IL-6, *p* = 0.006, IL-8, *p* < 0.001, IFN-α, *p* = 0.012, MX-1, *p* = 0.025).

Parallelly increased expression of SNHG16 and TLR4 was found in human and mouse AH lung tissues. Down-regulated TLR4 levels were shown in miR-146a-overexpressed MLE-12 cells as compared with control cells (Additional file 1: Fig. S8c, *p* < 0.001), while LV-mediated transfection of SNHG16 in such cells could reverse the targeting effect on TLR4 expression (Additional file 1: Fig. S8c, *p* = 0.001). These results indicated that SNHG16 could act as a ceRNA to upregulate the expression of TLR4, a proven target mRNA of miR-146a [29], in alveolar cells.

We further analyzed in situ apoptosis, autophagy and NETs formation in the pristane-induced AH lungs on day 14. TUNEL-positive apoptotic cells were found in pristane-injected but barely detected in PBS-injected lung tissues (Fig. 8f, i, pristane versus saline, 36.0 ± 10.4 versus 1.4 ± 0.6, *p* < 0.001). Cells expressing CitH3 colocalized with DNAs, in favor of NETs formation, were identified in pristane-injected but not in PBS-injected lung tissues (Fig. 8g, i, 25.2 ± 8.0 versus 0 ± 0, *p* < 0.001). Cytoplasmic LC3-positive cells, suggesting autophagy formation, was identified in pristane-injected but not in PBS-injected lung tissues (Fig. 8h, i, 9.0 ± 2.2 versus 0 ± 0, *p* < 0.001). Interestingly, by using immunoblot assay, increased levels of LC3-II were demonstrated in muscle tissues from pristine-injected mice [39], while there was an increase in LC3-II expression in lung tissues (Fig. 8j, *p* = 0.002). p53, Bax, LC3 and Beclin-1 levels were increased since day 4 by qRT-PCR analyses (Fig. 8k, day 4, for p53, *p* = 0.006, for Bax, *p* < 0.001, for LC3, *p* < 0.001, for Beclin-1, *p* < 0.001) in pristane-injected lung tissues.

### AH enhanced by intra-pulmonary LV-SNHG16 delivery through increasin cell death processes

**Fig. 9:**
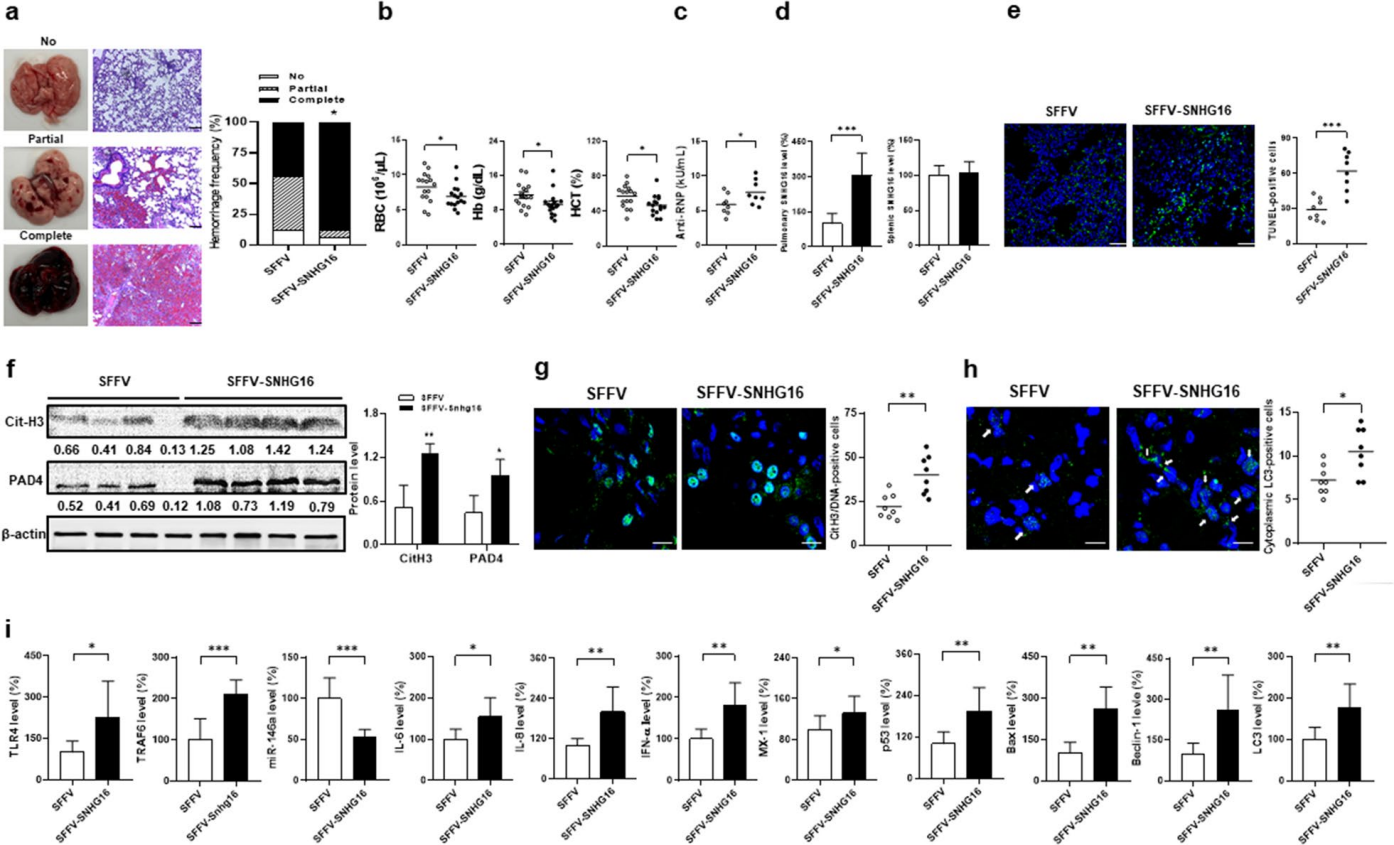
AH enhanced by intra-pulmonary SNHG16 delivery through increasing cell death processes in pristane-injected mice. **a** Left, representative gross and histopathological photographs in the mouse lungs with no, partial and complete hemorrhage. Scale bar = 100 µm, magnification ×100. Right, hemorrhagic frequencies in pristane-injected mice receiving intra-pulmonary delivery of SFFV or SFFV-SNHG16 on day 14. **b** RBC numbers, Hb levels and Hct on day 14 in mice receiving delivery of SFFV or SFFV-SNHG16. **c** Anti-RNP titers on day 14 in serum samples from SFFV- or SFFV-SNHG16-treated mice. **d** Left, pulmonary and right, splenic SNHG16 levels on day 14 from mice receiving delivery of SFFV or SFFV-SNHG16.** e** Left, representative TUNEL IF staining (green) in lung tissues from SFFV‑ or SFFV-SNHG16‑treated mice. Scale bar = 20 µm, magnification ×400. Right, quantification of TUNEL‑positive cell numbers in lung tissues. **f** Immunoblot assay (left) with signal intensity quantitation analysis (right) of pulmonary CitH3 and PAD4 expression from mice receiving delivery of SFFV or SFFV-SNHG16 on day 14. **g** Left, representative CitH3 IF staining (green) with cell nuclei counterstained with Hoechst 33258 (blue). Scale bar = 12.5 µm, magnification ×800. Right, quantification of cell numbers with positive colocalized CitH3 and DNAs in lung tissues.** h** Representative LC3 IF staining (green) with cell nuclei counterstained with Hoechst 33258 (blue). Scale bar = 10 µm, magnification ×1000. Right, quantification of cell numbers with positive cytoplasmic LC3 in lung tissues. **i** From left to right, pulmonary TLR4, TRAF6, miR-146a, IL-6, IL-8, IFN-α, MX-1, p53, Bax, LC3 and Beclin-1 levels on day 0, 4, 9 and 14 from mice receiving delivery of SFFV or SFFV-SNHG16. Values are mean ± SD. Horizontal lines are mean values. Mouse numbers per group, 16 in **a**, **b**, 8 in **c**, **d, e**, **g**, **h**, **i**, 4 in **f**. All results in figure were representative of 2 independent experiments with similar findings. *p < 0.05, **p < 0.01, ***p < 0.001

Since up-regulated pulmonary SNHG16 levels were identified in pristane-injected mice, we examined whether overexpressing this lncRNA by intra-pulmonary delivery can enhance AH. In comparison with control mice, pristane-injected mice receiving SFFV-SNHG16 delivery had higher complete hemorrhage frequencies (Fig. 9a, 87.5% versus 43.8%, *p* = 0.023), and lower RBC numbers, Hb levels and Hct (Fig. 9b, for RBC, *p* = 0.048, for Hb, *p* = 0.045, for Hct, *p* = 0.023). Higher anti-RNP titers were identified in mice receiving the SFFV-SNHG16 delivery (Fig. 9c, *p* = 0.049). In SFFV-SNHG16-treated mice, there were higher pulmonary SNHG16 levels (Fig. 9d, *p* < 0.001), while no differences were found in splenic expression. Higher TUNEL-positive apoptotic cell numbers (Fig. 9e, 62.0 ± 16.3 versus 29.0 ± 9.4, *p* < 0.001) and increased p53 and Bax levels (Fig. 9h, for p53 *p* = 0.004, for Bax, *p* = 0.006) were found in SNHG16-overexpressed AH lung tissues. SFFV-SNHG16-treated AH lung tissues had higher CitH3/PAD4 protein levels (Fig. 9f, for CitH3, *p* = 0.005, for PAD4, *p* = 0.020) and numbers of cells expressing CitH3 colocalized with DNAs (Fig. 9g, 40.0 ± 11.0 versus 22.0 ± 7.0, *p* = 0.002), indicating enhanced in situ NETs formation. Furthermore, there were higher numbers of cytoplasmic LC3-positive cells (Fig. 9g, 10.5 ± 2.7 versus 7.3 ± 1.7, *p* = 0.012), and LC3 and Beclin-1 levels in SFFV-SNHG16-treated AH lung tissues (Fig. 9i, for LC3, *p* = 0.009, for Beclin-1, *p* = 0.005), suggesting an increase in autophagy formation. In addition, pulmonary TLR4, TRAF6, IL-6, IL-8, IFN-α and MX-1 levels were higher, whereas miR-146a levels were lower in SFFV-SNHG16-treated mice (Fig. 9i, for TLR4, *p* = 0.021, for miR-146a, *p* < 0.001, for TRAF6, *p* < 0.001, for IL-6,* p* = 0.011, for IL-8, *p* = 0.003, for IFN-α, *p* = 0.002, for MX-1, *p* = 0.044).

### AH suppressed by intra-pulmonary prophylactic and early therapeutic sh-SNHG16 delivery through reducing cell death processes

**Fig. 10:**
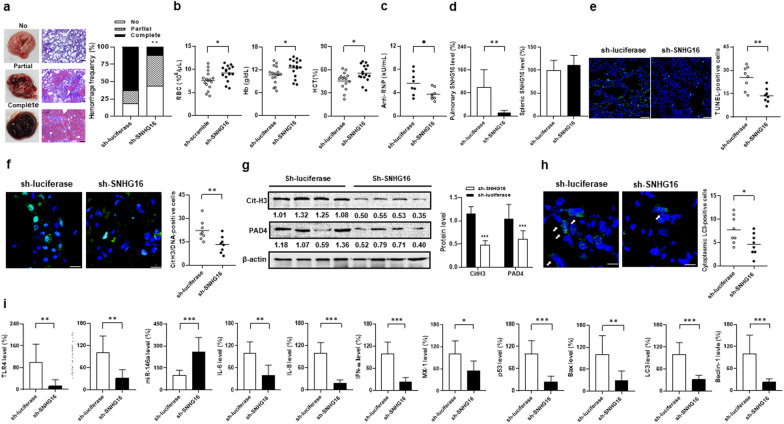
AH suppressed by intra-pulmonary prophylactic sh-SNHG16 delivery through reducing cell death processes in pristane-injected mice. **a** Left, representative gross and histopathological photographs in the mouse lungs with no, partial and complete hemorrhage. Scale bar = 100 µm, magnification ×100. Right, hemorrhagic frequencies on day 14 in pristane-injected mice receiving intra-pulmonary delivery of sh-luciferase or sh-SNHG16. **b** RBC numbers, Hb levels and Hct on day 14 in mice receiving delivery of sh-luciferase or sh-SNHG16. **c** Anti-RNP titers on day 14 in serum samples from mice receiving delivery of sh-luciferase or sh-SNHG16. **d** Pulmonary (left) and splenic (right) SNHG16 levels on day 14 from mice receiving delivery of sh-luciferase or sh-SNHG16.** e** Left, representative TUNEL IF staining (green) in lung tissues from sh‑luciferase- and sh-SNHG16‑treated mice. Scale bar = 25 µm, magnification ×400. Right, quantification of TUNEL‑positive cell numbers in lung tissues. **f** Left, representative CitH3 IF staining (green) with cell nuclei counterstained with Hoechst 33258 (blue). Scale bar = 12.5 µm, magnification ×800. Right, quantification of cell numbers with positive colocalized CitH3 and DNAs in lung tissues. **g** Immunoblot assay (left) with signal intensity quantitation analysis (right) of pulmonary CitH3 and PAD4 expression on day 14 from mice receiving delivery of sh-luciferase or sh-SNHG16. **h** Left, representative LC3 IF staining (green) with cell nuclei counterstained with Hoechst 33258 (blue). Scale bar = 10 µm, magnification ×1000. Right, quantification of cell numbers with positive cytoplasmic LC3 in lung tissues. **i** From left to right, pulmonary TLR4, TRAF6, miR-146a, IL-6, IL-8, IFN-α, MX-1, p53. Bax, LC3 and Beclin-1 levels on day 0, 4, 9 and 14 from mice receiving delivery of sh-luciferase or sh-SNHG16. Values are mean ± SD. Horizontal lines are mean values. Mouse numbers per group, 16 in **a**, **b**, 8 in **c**, **d**, **e**, **f, h**,** i**, 4 in **g**. All results in figure were representative of 2 independent experiments with similar findings. *p < 0.05, **p < 0.01, ***p < 0.001

We further evaluated whether silencing the expression of pulmonary SNHG16 can suppress the AH lungs. Pristane-injected mice receiving intra-pulmonary sh-SNHG16 delivery on day − 1, i.e. prophylactic intervention, had lower complete hemorrhage frequencies than controls under sh-luciferase therapy (Fig. 10a, 12.5% versus 62.5%, *p* = 0.009). There were higher RBC numbers, Hb levels and Hct in sh-SNHG16- than sh-luciferase-treated mice (Fig. 10b, for RBC, *p* = 0.015, for Hb, *p* = 0.034, for Hct, *p* = 0.014). Lower anti-RNP titers on day 14 were identified in mice receiving the sh-SNHG16 than sh-luciferase delivery (Fig. 10c, *p* = 0.028). There were lower pulmonary SNHG16 levels in the sh-SNHG16-treated mice (Fig. 10d, *p* = 0.001), but no differences in the splenic expression. Lower TUNEL-positive apoptotic cell numbers and p53/Bax levels (Fig. 10e, for apoptotic cell, 25.4 ± 7.4 versus 13.8 ± 4.7, *p* = 0.002; Fig. 10i, for p53 *p* < 0.001, for Bax, *p* = 0.004) were found in SNHG16-silenced lung tissues. For NETosis-related CitH3 and PAD4 expression, the sh-SNHG16-treated lungs had lower CitH3/DNA-positive cell numbers and decreased pulmonary CitH3 and PAD4 protein levels (Fig. 10g, for CitH3/DNA-positive cell, 22.3 ± 6.4 versus 13.3 ± 5.3, *p* = 0.008; for CitH3 and PAD4, *p* < 0.001), indicating reduced in situ NETs formation. Moreover, there were lower cytoplasmic LC3-positive cell number (Fig. 10h, *p* = 0.009) and decreased LC3 and Beclin-1 levels (Fig. 10h, for LC3-positive cell, 7.8 ± 3.0 versus 4.6 ± 2.3, *p* = 0.034; Fig. 10i, for LC3 and Beclin-1, *p* < 0.001), suggesting an decrease in autophagy formation. In addition, TLR4, TRAF6, IL-6, IL-8, IFN-α and MX-1 levels were lower, whereas miR-146a levels were higher in sh-SNHG16-treated lung tissues (Fig. 10i, for TLR4, *p* = 0.003, for TRAF6, *p* = 0.002, for miR-146a, *p* < 0.001, for IL-6,* p* = 0.001, for IL-8, *p* < 0.001, for IFN-α, *p* < 0.001, for MX-1, *p* = 0.012).

Interestingly, SNHG16 has been reported to facilitate cell autophagy via sponging miR-542-3p to upregulate ATG5, a molecule participating in the formation of autophagosome [40]. A dose/time-dependent increase in ATG5 expression was shown in alveolar cell culture under LPS stimulation (Additional file 1: Fig. S11a), while up-regulated ATG5 levels were found in pristane-induced AH lung tissues since day 4 (Additional file 1: Fig. S11b, day 4, *p* < 0.001). There were increased and decreased pulmonary ATG5 expression in SNHG16-overexpressed and -silenced pristine-injected mice on day 14, respectively (Additional file 1: Fig. S11c, for overexpressed, *p* = 0.046, for silenced, *p* = 0.043), suggesting that, in addition to LC3 and Beclin-1, SNHG16 could regulate the autophagic process through modulating the ATG5 expression in the AH lungs.

**Fig. 11:**
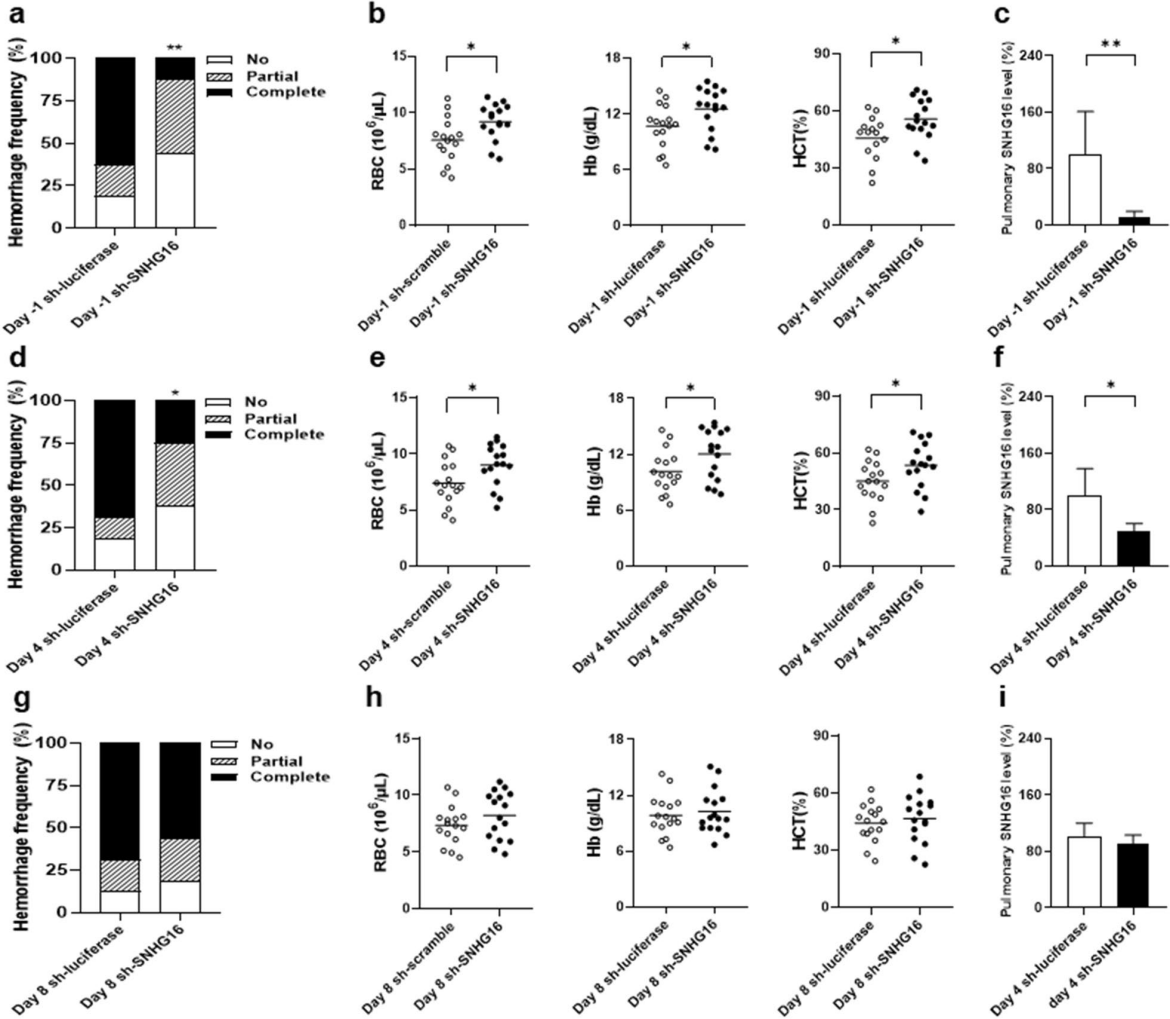
AH in pristane-injected mice receiving intra-pulmonary sh-SNHG16 delivery on different days. Hemorrhagic frequencies on day 14 in pristane-injected mice receiving **a** day − 1 prophylactic, **d** day 4 early therapeutic and **g** day 8 late therapeutic intra-pulmonary delivery of sh-luciferase or sh-SNHG16. RBC numbers, Hb levels and Hct on day 14 in mice receiving **b** day − 1 prophylactic, **e** day 4 early therapeutic and **h** day 8 late therapeutic intra-pulmonary delivery of sh-luciferase or sh-SNHG16. Pulmonary SNHG16 levels on day 14 in mice receiving **c** day − 1 prophylactic, **f** day 4 early therapeutic and **i** day 8 late therapeutic intra-pulmonary delivery of sh-luciferase or sh-SNHG16. Values are mean ± SD. Horizontal lines are mean values. Mouse numbers per group, 16 in **a**, **b**, **d**, **e**, **g**, **h**, 8 in **c**, **f**, **i**. All results in figure were representative of 2 independent experiments with similar findings. *p < 0.05, **p < 0.01, ***p < 0.001

Pristane-induced AH mice further received intra-pulmonary sh-SNHG16 delivery on day 4 and 8, i.e. an early and a late therapeutic intervention. Lower complete hemorrhage frequencies were found in mice receiving sh-SNHG16 delivery than controls under sh-luciferase therapy on day 4 (Fig. 11d, 25% versus 68.8%,* p* = 0.032), but not on day 8 (Fig. 11g, 56.3% versus 68.8%). There were higher RBC numbers, Hb levels and Hct in mice treated with sh-SNHG16 delivery than controls receiving sh-luciferase treatment on day 4 (Fig. 11e, for RBC, *p* = 0.026, for Hb, *p* = 0.044, for Hct, *p* = 0.046), but no differences were identified on day 8 (Fig. 11h). In addition, pulmonary SNHG16 levels were lower in sh-SNHG16-infused than sh-luciferase-treated mice on day 4 (Fig. 11f, 100.0 ± 38.2% versus 49.2 ± 30.8%, *p* = 0.011), but not on day 8 (Fig. 11i, 100.0 ± 20.0% versus 90.4 ± 13.0%). These findings indicated the better efficacy in AH suppression by an earlier than a later therapeutic silence of SNHG16 expression in the lungs. For comparison with therapeutic intervention, the results of complete hemorrhage frequencies (Fig. 10a), RBC numbers, Hb levels and Hct (Fig. 10b), and pulmonary SNHG16 levels (Fig. 10d) in mice receiving prophylactic sh-SNHG16/sh-luciferase therapy on day − 1 were also demonstrated in Fig. 11a–c respectively.

Notably, except the lungs, the examinations after scarification in pristane-injected mice receiving intra-tracheal delivery of LV-SNHG16, sh-SNHG16 or their control vectors on day 14 revealed no morphological abnormalities in other visceral organs, indicating no evident extra-pulmonary global impact related to the intra-pulmonary administration of these LV vectors.

**Fig. 12:**
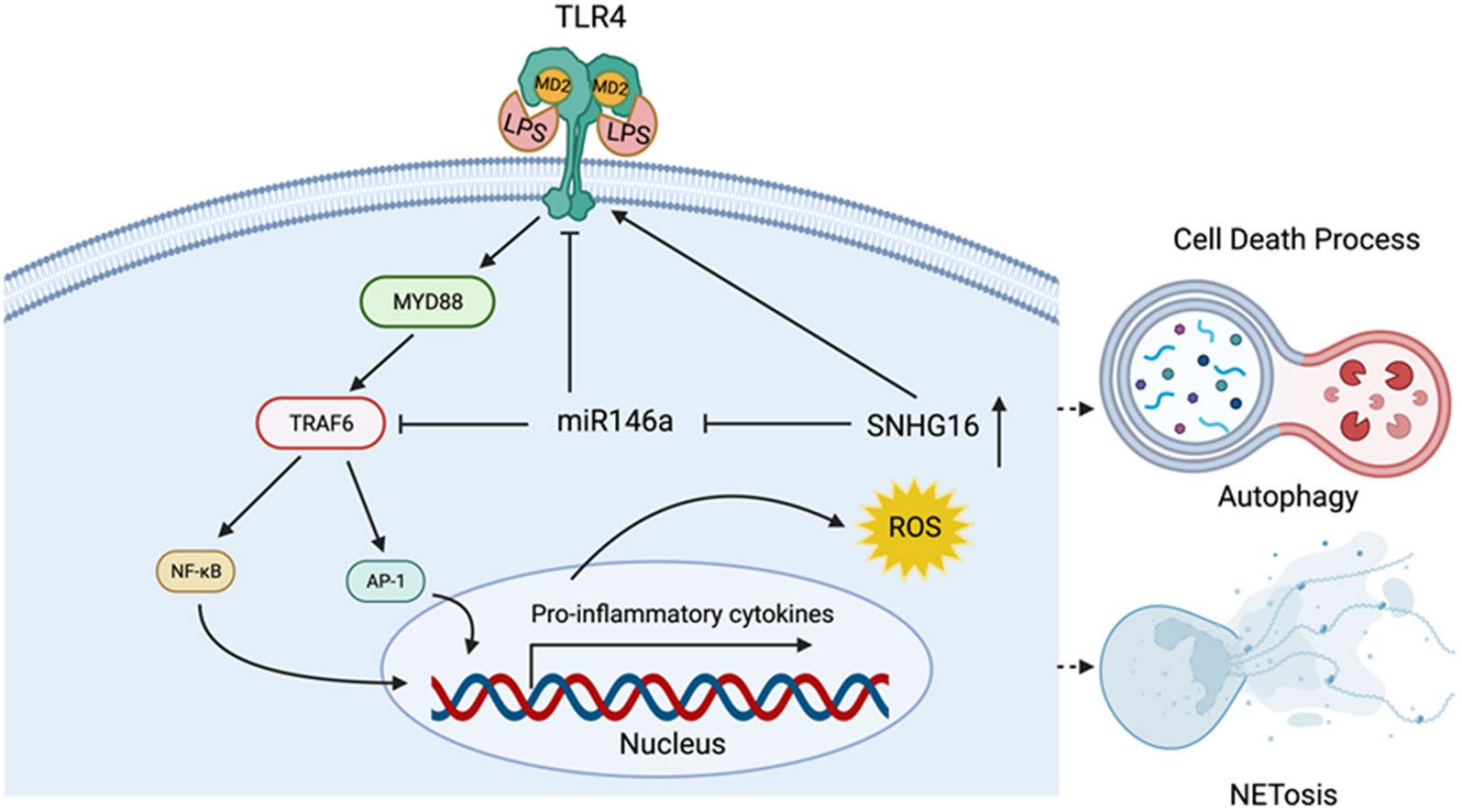
A schematic summary of the latest evidence and experimental results in this study. Under the specific intra-pulmonary cytokine milieu with increased IL-6 expression in SLE-AH lung tissues, upregulated SNHG16 levels in alveolar cells and resident neutrophils can enhance TLR4 expression via stabilizing its mRNA expression and sponging miRNAs that can target it, e.g. miR-146a. TRAF6, a miR-146a target molecule, is a potent inducer of autophagy and NETosis. TRAF6, a miR-146a target molecule, is an inducer of autophagy and NETosis. SNHG16 can regulate the TLR4/TRAF6 axis and activate downstream genes to generate pro-inflammatory cytokines like IL-8, a potent inducer of NETosis. Under high ROS levels, the autophagic process is initiated through the inactivation of mTOR pathway. Figure is created with BioRender.com

Figure 12 is a schematic summary of the latest evidence and aforementioned experimental results in this study. Under the specific intra-pulmonary cytokine milieu with increased IL-6 expression in the SLE-AH lungs, upregulated SNHG16 levels in alveolar cells and resident neutrophils can enhance TLR4 expression via stabilizing its mRNA expression and sponging miRNAs that can target it, e.g. miR-146a [27–29]. TRAF6, a miR-146a target molecule, is a potent inducer of autophagy and NETosis [23, 24]. SNHG16 can regulate the TLR4/TRAF6 axis and activate downstream genes to generate pro-inflammatory cytokines like IL-8, a potent inducer of NETosis [24]. Under higher levels of reactive oxygen species (ROS), the autophagic process is initiated through the inactivation of mTOR pathway [41].

### Down-regulated renal SNHG16 expression in LN patients and a pristane-induced mouse LN model

LN patients had higher SNHG16 levels in their PBMCs than those without renal involvement or HC (Additional file 1: Fig. S12a, LN versus Nil, 709.9 ± 992.2 versus 231.0 ± 285.3%, *p* = 0.024, or versus HC, 100.0 ± 158.4%, *p* < 0.001). Nevertheless, SNHG16 levels in USCs were lower in LN patients than those without renal involvement or HC (Additional file 1: Fig, S12b, LN versus Nil, 47.7 ± 33.7 versus 78.6 ± 40.7%, *p* = 0.034, or versus HC, 100.0 ± 66.0%, *p* = 0.022), while renal SNHG16 levels were also lower in LN than control specimens (Additional file 1: Fig, S12c, LN versus control, 10.4 ± 8.8 versus 100.0 ± 57.4%, *p* = 0.016). No differences were identified in PBMC and USC NEAT1 levels between patients with LN and without renal involvement (Additional file 1: Fig. S12d, S12e), or in renal SNHG16 levels between LN and control specimens (Additional file 1: Fig. S12f).

BALB/c mice had significant proteinuria and elevated levels of anti-RNP and anti-dsDNA at month 5 and 6 after the pristane injection (Additional file 1: Fig. S13a, for proteinuria, month 5, *p* = 0.028, month 6, *p* < 0.001; Additional file 1: Fig. S13b, for anti-RNP, month 5, *p* = 0.016, month 6, *p* = 0.007; Additional file 1: Fig. S13c, for anti-dsDNA, month 5, *p* = 0.001, month 6, *p* < 0.001). There was GN formation with hypercellularity and mesangial expansion in glomeruli at month 6 after the pristane injection (Additional file 1: Fig. S13d, representative histopathology analysis). Furthermore, SNHG16 expression was examined in the kidneys at month 1, 3, 5 and 6. At an earlier phase after pristane injection, similar to AH lung tissues on day 14, there was up-regulated SNHG16 expression at month 1, whereas down-regulated expression occurred since month 5 (Additional file 1: Fig. S13e, pristane versus saline, for month 1, 139.1 ± 21.0 versus 99.1 ± 18.2%, *p* = 0.011; for month 5, 74.0 ± 11.0 versus 101.2 ± 18.1%, *p* = 0.021; for month 6, 45.2 ± 9.5 versus 102.6 ± 25.5%, *p* = 0.002). These results demonstrated down-regulated SNHG16 levels in renal tissues of full-brown LN, contrary to up-regulated levels in the AH lungs. Since the expression of lncRNAs is closely regulated by cytokine milieu, such inconsistent findings might be related to distinct cytokine profiles in different SLE target organs, proinflammatory cytokines IL-6/IL-8 in the lungs versus Th1 cytokines IFN-γ/IL-2 in the kidneys [24].

## Discussion

Based on the morphology, triggers and involved pathways, cell death processes can be classified into various categories such as apoptosis, autophagy and NETosis [42]. Apoptosis, a programmed cell death causing cellular self-destruction, controls cell turnover, embryonic development and body growth to maintain homeostasis [43]. Autophagy characterized by autophagosomes to digest unused components and damaged organelles through fusing with lysosomes, allows for recycling to provide nutrients to promote cell survival [44]. NETosis, a regulated necrotic death pathway in neutrophils, is a specialized process with the formation of NETs [45]. All of above death processes have been shown to be involved in the disease onset and flare of SLE [3]. In particular, by using genome-wide association studies, autophagy-related genes like ATG5 were found to be associated with disease susceptibility in SLE, while risk alleles of variants locating in PRDM1-ATG5 gene region were positively correlated with ATG5 expression in B cells from lupus patients [46]. In this study, AH patients had higher p53 levels, apoptotic cell ratios and Beclin-1 levels in their PBMCs than those from other patient groups. By LPS stimulation, in comparison with other patient groups, AH patients had higher Beclin-1 levels in PBMs and greater NETs percentages and CitH3/PAD4 concentrations in PBNs. These results indicated that such patients have more severe cell death in PB leukocytes than other patient groups in SLE. Furthermore, in the SLE-AH lungs, there were higher TUNEL-positive apoptotic cell numbers and up-regulated levels of p53/Bax, increased NETs formation with colocalized CitH3/DNAs and elevated PAD4 levels, and the presence of autophagy with cytoplasmic LC3-positive cells and up-regulated LC3/Beclin-1 expression. Altogether, the aforementioned findings implicate that, in active lupus patients, apoptosis, autophagy and NETosis take place in their lungs, providing nuclear autoantigens for IC formation with deposition in pulmonary capillary wall, ultimately resulting in capillaritis and AH.

Interestingly, distinct NETs formation was also observed in AAV-related AH lung tissues in this study. Unlike SLE, AAV is an autoimmune disease lacking IC formation, while ANCA binding to the membrane-bound myeloperoxidase on resident neutrophils can bring about intra-pulmonary NETosis in such patients [36]. Similar to SLE, IgAV is another IC-mediated autoimmune disorder with pulmonary capillaritis and AH [47]. Nevertheless, cell death processes were not identified in IgAV-AH lung tissues. Exogenous antigens derived from microorganisms or medications rather than endogenous nuclear autoantigens released from cell death are responsible for the IC formation in IgAV. Despite no available APS-AH lung tissues for evaluating cell death processes in this investigation, such a respiratory manifestation is known as a non-thrombotic inflammatory process unrelated to the IC-mediated pathogenesis [33]. Moreover, there were no differences in SNHG16, TLR4 or TRAF6 levels in PBMCs between control subjects and non-SLE AH from AAV, IgAV and APS patients. Altogether, these results suggest complex molecular mechanisms involving in the immune-mediated AH with distinct dissimilarities among different underlying autoimmune diseases.

In SLE patients, elevated circulating levels of pro-inflammatory cytokines like TNF-α, IL-6 and IL-8 are positively correlated with the activity scores [48]. Upregulated expression of specific lncRNAs has been demonstrated to be in parallel with SLE disease activity [49], while the expression of cellular lncRNAs is highly responsive to the surrounding cytokine milieu [50]. Our earlier experiments revealed increased lincRNA-p21 expression in PBMCs positively correlating with the activity scores, and a dose-dependent up-regulated lincRNA-p21 expression in the culture of T cells under the stimulation of TNF-α [35]. Furthermore, increased lincRNA-p21 levels with in situ apoptosis were identified in SLE-AH lung tissues [15]. In this study, we identified higher levels of SNHG16 in PBMCs or PBNs with a positive correlation with disease activity in SLE, while active lupus patients had increased cell death processes in their PB leukocytes. Since a crucial pathogenic mechanism in SLE is accelerated cell death to release nuclear autoantigens for IC formation, upregulated SNHG16 expression in circulating leukocytes during the active disease appears to be involved in the death processes. In the presence of IL-6, there was a dose-dependent increase in SNHG16 expression in the cultured alveolar cells and neutrophils. Furthermore, up-regulated IL-6 and SNHG16 levels with increased in situ apoptosis, autophagy and NETosis were identified in both human and mouse AH lung tissues. Taken together, these data propose that, during the active disease in SLE patients, increased circulating levels of IL-6 can upregulate the expression of SNHG16 to enhance cell death processes.

The SLE pathogenesis-related innate immune receptors include endosomal TLR7/9 binding nucleic acids and cell membrane-bound TLR2/4 binding DAMP molecules like HMGB1, further contributing to the enhanced autoimmune responses [51, 52]. The contribution of membrane-bound TLR4 to the development of autoimmunity has been consistently demonstrated in transgenic lupus mouse models [53]. Mice overexpressed with TLR4 were shown to generate the SLE-like autoimmunity [54], while upregulated TLR4 levels were found in pulmonary and splenic tissues from pristane-induced lupus mice in this study. In active SLE patients, there were increased circulating HMGB1 concentrations and upregulated TLR4 levels in PBMCs [52, 55]. In our investigation, SNHG16 and TLR4 expression in PB leukocytes were in a positive correlation, while their levels were higher in AH patients than other patient groups. Furthermore, there were increased pulmonary HGMB1 expression and up-regulated SNHG16 and TLR4 levels in lung tissues from SLE-AH patients. A positive correlation existed between TLR4 and SNHG16 expression in PB leukocytes from SLE patients. Up-regulated SNHG16 expression and TLR4-mediated NETs formation were shown in mouse neutrophils under HMGB1 or LPS stimulation. There was enhanced LPS-induced autophagy in SNHG16-overexpressed and reduced autophagy in SNHG16-silenced alveolar cells. Increased LPS-induced NETosis was found in SNHG16-overexpressed myelocytic cells, while there was reduced NETosis in SNHG16-silenced cells. LV-mediated transfection of SNHG16 in miR-146a overexpressed alveolar cells could reverse the targeting effect of miR-146a on TLR4 expression, indicating a ceRNA role of SNHG16 to upregulate TLR4 expression via targeting miR-146a. Intra-pulmonary overexpressing and silencing the expression of SNHG16 in pristane-induced AH mice could up-regulate and down-regulate TLR4 expression with enhanced and reduced cell death processes, respectively. Collectively, these discoveries display that, up-regulated pulmonary SNHG16 expression in active SLE can enhance TLR4-mediated autophagy and NETs formation, leading to the AH manifestation.

In vitro pristane-induced apoptosis in T cells could be blocked by inhibiting the activity of caspase 9 rather than caspase 8, implying the involvement of a p53-related intrinsic apoptosis pathway [56]. Furthermore, in pristane-induced C57BL/6 strain lpr mice with a Fas mutation, there were no reduced AH frequencies with preserved sensitivity of in vitro pristane-induced cell apoptosis, suggesting the independence of Fas-related extrinsic apoptosis pathway in this model [57]. In this study, up-regulated SNHG16 and p53/Bax and down-regulated miR-146a expression were demonstrated in alveolar cells stimulated with Dox, a DNA damage inducer to trigger the p53-dependent apoptosis [15], and in lung tissues of pristane-induced AH mice with enhanced in situ apoptosis. There were increased Dox-induced apoptosis in SNHG16-overexpressed cells with up-regulated p53/Bax and down-regulated miR-146a expression, and reduced apoptosis in SNHG16-silenced cells with down-regulated p53/Bax and up-regulated miR-146a expression. Furthermore, AH was enhanced by intra-pulmonary LV-SNHG16 delivery through increasing p53/Bax and down-regulating miR-146a expression to enhance apoptosis, while it was suppressed by sh-SNHG16 delivery through decreasing p53/Bax and up-regulating miR-146a levels to reduce apoptosis. These experimental results revealed that SNHG16 can regulate the p53-related intrinsic apoptosis pathway by sponging miR-146a to reverse its targeting effects on TAF9b, a p53 co-activator/stabilizer [24]. Interestingly, TRAF6, a miR-146a target molecule, is able to induce the extrinsic apoptosis pathway by interacting and activating caspase 8 [22]. Although the pristane-induced AH mouse model is independent of extrinsic apoptosis pathway, such an apoptotic process has been demonstrated to be involved in the pathogenesis of disease activity in SLE patients [3]. Further studies should examine whether SNHG16 can modulate the TLR4-mediated apoptosis by using LPS to induce the extrinsic apoptotic process.

Clinically, AH can be classified into non-immune and immune-mediated etiologies, while the latter category includes post-transplantation, drug-induced vasculitis and autoimmune diseases [5]. In particular, despite intensive respiratory support, SLE-associated AH has an average mortality rate around 40% based on accumulated statistics from large-scale series since 2010s [5, 15]. In addition to the routine use of high-dose corticosteroids or plus pulse methylprednisolone, current medications such as cyclophosphamide and intravenous immunoglobulin are associated with unsatisfying responses in SLE-AH patients [58]. In spite of an extensive application in refractory cases, therapeutic plasmapheresis lacks a beneficent impact on the overall fatality [59]. An improved survival has been observed in applying rituximab (RTX), a B-cell depleting antibody, to reduce the formation of IC [60]. Nevertheless, this biologic agent can harm antiviral humoral immunity and cause a difficulty in cleaning the invasion of severe acute respiratory syndrome-coronavirus-2 (SARS-CoV-2) [61]. RTX use in SLE has been reported to be associated with poor vaccination efficacy, prolong hospitalization and death outcomes during the pandemic SARS-CoV-2 infection [62]. Indeed, there is an imperative need to develop effective and secure therapeutics in controlling the disease activity to avoid mortality in SLE-associated AH.

Current investigation on the inhibition of NETosis pathway or NETs degrading drugs can provide potential therapeutic approach for life-threatening complications in autoimmune diseases, such as the AH manifestation in SLE patients [63]. Available SLE medications including cyclosporin, hydroxychloroquine and tacrolimus can target NETs components or interfere with the formation of NETs; however, there are no recently approved anti-NETs drugs to treat lupus patients [64]. The anti-NETs approach has demonstrated therapeutic efficacy in animal models of SLE. In addition to sh-SNHG16 intra-tracheal delivery in our investigation, DNase-1 inhalation to reduce NETs could suppress the AH manifestation in the pristane-induced mouse model, while the injection of Cl-amidine, a PADs inhibitor, protected MRL/lpr lupus mice from NETs-mediated visceral injury [63]. Nevertheless, considering the risk of systemic infections in NETs-depleted individuals, further studies are necessary for the development of effective clinical compounds able to safely treat such patients [63, 64].

In this study, intra-pulmonary delivery of shRNA targeting SNHG16 could reduce AH through inhibiting TLR4-mediated autophagy and NETs formation in the lungs of a mouse model. By intra-tracheal infusion of LV-sh-SNHG16, lower pulmonary SNHG16 levels were observed without differences in splenic expression, indicating no extra-pulmonary leakage of infused LV vectors. Besides, there were no undesired effects outside the lungs as shown by no morphological abnormalities in other visceral organs after the scarification of mice. Although safety always is a crucial issue for applying LV vectors into clinical practice, the intra-pulmonary delivery route can evade the adverse impacts on non-target organs through the systemic administration. Infusion of activated recombinant factor VII via an intra-pulmonary route by nebulizer or bronchoscopy has been shown to complete hemostasis and improve AH without adverse effects in SLE-AH patients [65, 66]. Interestingly, RNA therapeutics are emerging treatment modalities with 17 approved clinical products, including 2 macromolecular mRNA drugs and 15 oligonucleotide drugs with one aptamer, 4 siRNAs and 10 antisense oligonucleotides [67]. In particular, despite not through the intra-pulmonary administration, there are ongoing siRNA therapeutic trials focusing on lung malignancies with favorable development prospects [67, 68]. Notably, our experimental results implicated a therapeutic potential in the SLE-associated AH lungs by intra-pulmonary infusion of shRNA targeting SNHG16 to reduce in situ cell death processes.

## Conclusions

Our experimental results demonstrate firstly that lncRNA SNHG16 regulates TLR4-mediated autophagy and NETs formation in the human and mouse AH lungs, and provide a potential of intra-pulmonary delivery of shRNA targeting SNHG16 to treat such a lethal manifestation in SLE patients.

### Supplementary Information

**Additional file 1:** **Fig. S1.** SNHG16 expression in LV-SNHG16‑ and sh-SNHG16-transfected cells. **a** Left, map of pLV-SFFV-SNHG16-PGK-puro, a total of 9.3 kb in length. Right, SNHG16 levels in LV-SNHG16‑transfected 293T cells.** b** Left, map of pLKO.1-sh-SNHG16-puro, a total of 7.4 kb in length. Right, SNHG16 levels in sh-SNHG16‑transfected MLE-12 cells. Values are mean ± SD. Results in Fig. S1a were representative of 3 independent experiments, and in Fig. S1b were representative of 2 independent experiments with similar findings. **Fig. S2.** Expression of SNHG16 in purified neutrophils and sorted T cells, monocytes and B cells from PB of healthy individuals.** a** Flow cytometric graphs of sorted CD3-positive T cells, CD14-positive monocytes and CD19-positive B cells from healthy individual No. 1, No. 2 and No. 3. **b** PB cell numbers/µL (left) and SNHG16 levels/10^5^ cells (right) in neutrophils, T cells, monocytes and B cells from healthy individual No. 1, No. 2 and No. 3. Values are mean ± SD. **Fig. S3.** Expression of miR-146a in PBMCs and PBNs from SLE patients. **a** MiR-146a levels in PBMCs from SLE patients and HC. **b** A negative correlation between miR-146a levels in PBMCs and SLEDAI-2K activity scores. **c** MiR-146a levels in PBMCs from HC, Nil. LN, SLE-AH and other AH patients. A negative correlation between miR-146a and **d** TLR4, **e** TRAF6 and **f** NEAT1 levels in PBMCs from SLE patients. **g** MiR-146a levels in PBNs from SLE patients and HC. **h** A negative correlation between miR-146a levels in PBNs and SLEDAI-2K activity scores. **i** MiR-146a levels in PBNs from HC, Nil. LN and AH patients. Values are mean ± SD. Horizontal lines are mean values. Patient numbers, n = 62 for PBMCs from SLE, 15 for PBNs from SLE, 7 for PBMCs from Nil, LN and SLE-AH, 6 for PBMCs from other AH, 5 for PBNs from Nil, LN, 4 for PBN from AH. *p < 0.05. **p < 0.01, ***p < 0.001. **Fig. S4.** Expression of NEAT1 in SLE patients and pristane-injected mice. NEAT1 levels in **a** PBMCs and **e** PBNs from SLE patients and HC. **b** A positive correlation and **f** no correlation between NEAT1 levels in PBMCs and PBNs from SLE patients, respectively, and SLEDAI-2K activity scores. **d** No correlation between NEAT1 and TLR4 levels in PBMCs from SLE patients. NEAT1 levels in **c** PBMCs and **g** PBNs from HC, Nil, LN and AH patients. **h** NEAT1 levels in thioglycolate-induced mouse neutrophils from pristane-induced AH mice. NEAT1 levels in **i** lung and **j** spleen tissues from pristane-induced AH mice. Values are mean ± SD. Horizontal lines are mean values. Patient numbers, n = 62 for PBMCs from SLE, 15 for PBNs from SLE, 7 for PBMCs from Nil, LN, SLE-AH, 6 for PBMCs from other AH, 5 for PBNS from Nil, LN, 4 for PBNs from AH. 4 mice per group in **h**. 5 mice per group in **i**, **j**. All results in Fig. S4**h,** S4**i** and S4**j** were representative of 2 independent experiments with similar findings. * *p* < 0.05. **Fig. S5.** p53 levels and apoptotic cell ratios in PBMCs from SLE patients. **a** Left, p53 levels in SLE patients and HC. Right, p53 levels in HC, Nil, LN and AH. **b** Left, apoptotic cell ratios in SLE patients and HC. Right, apoptotic cell ratios in HC, Nil, LN and AH. Values are mean ± SD. Horizontal lines are mean values. Patient numbers, n = 30 for SLE, n = 5 for Nil, n = 5 for LN, n = 3 for AH. *p < 0.05, **p < 0.01, ***p < 0.001. **Fig. S6.** Increased autophagy formation by immunoblot assay in PBMCs from SLE patients. **a** Representative immunoblot assay for Beclin-1, LC and mTOR in PBMCs from SLE patients and HC. **b** Signal intensity quantitation analysis for Beclin-1, LC and mTOR in PBMCs from SLE patients and HC. Values are mean ± SD. Patient numbers, n = 3 for representative immunoblot assay, n = 10 for signal intensity quantitation analysis. * *p* < 0.05. **Fig. S7.** Apoptosis formation in Dox-stimulated MLE-12 cells regulated by SNHG16. **a** SNHG16 expression in MLE‑12 cells stimulated with various concentrations of IL‑6. **b** Left, representative photographs of TUNEL IF staining (green) in MLE‑12 cells stimulated with 1 μM Dox. Cell nuclei counterstained with DAPI (blue). Scale bar = 10 µm, magnification ×1000. Middle, quantification of TUNEL‑positive cell percentages in mock and stimulation with 1 μM Dox. Right, apoptotic cell ratios in MLE-12 cells stimulated with various concentration of Dox. **c** HMGB1 levels in culture supernatants of MLE-12 cells stimulated with various concentration of Dox. **d** From left to right, expression of SNHG16, TRAF6, p53, Bax and miR-146a levels by qRT-PCR analyses in MLE-12 cells stimulated with various concentration of Dox. **e** Representative immunoblot assay of p53/Bax (top) and TRAF6 (low) expression in MLE-12 cells stimulated with various concentration of Dox. **f** MiR-17 and miR-146a levels in SNHG16-overexpressed and -silenced MLE-12 cells. **g** Left, SNHG16 expression in SNHG16-overexpressed MLE-12 transfectants. Right, from left to right, levels of p53, Bax and SNHG16 and apoptotic cell ratios in 1 μM Dox-stimulated SNHG16-overexpressed MLE-12 transfectants. **h** Left, SNHG16 expression in SNHG16-silenced MLE-12 transfectants. Right, from left to right, levels of p53, Bax and SNHG16 and apoptotic cell ratios in 1 μM Dox-stimulated SNHG16-silenced MLE-12 transfectants. Values are mean ± SD. Results in Fig. S7**a** to S7**f** were representative of 3 independent experiments, and in Fig. S7**g** and S7**h** were representative of 2 independent experiments with similar findings. *p < 0.05, **p < 0.01, ***p < 0.001. **Fig. S8.** TLR4 expression in SNHG16-overexpressed/silenced and miR‑146a‑ overexpressed MLE‑12 cells. **a** TLR4 mRNA levels in SNHG16-overexpressed (left) and -silenced MLE-12 transfectants (right).** b** Representative immunoblot assay of TLR4 levels in SNHG16-overexpressed (left) and -silenced MLE-12 transfectants (right). **c** Left, miR-146a levels in miR‑146a‑overexpressed MLE‑12 transfectants. Right, TLR4 levels in miR‑146a‑overexpressed MLE‑12 transfectants and in such cells overexpressed with SNHG16. Values are mean ± SD. Results in Fig. S8**a** and S8**b** were representative of 3 independent experiments, and in Fig. S8**c** were representative of 2 independent experiments with similar findings. **p < 0.01, ***p < 0.001. **Fig. S9.** Involvement of SNHG16 in TLR4-mediated NETs formation in mouse neutrophils. **a** Expression of SNHG16, TLR4, TRAF6 and miR-146a in thioglycolate-induced neutrophils from pristane-injected mice on day 4, day 9 and day 14. **b** SNHG16 expression in naïve mouse neutrophils stimulated with various concentrations of IL‑6 or 3 μg/mL LPS. **c** Naïve mouse neutrophils stimulated with various concentrations of HMGB1 or LPS. Left, representative photographs of NETs morphology from naïve mouse neutrophils under 300 ng/mL HMGB1 or 3 μg/mL LPS stimulation. Scale bar = 30 µm, magnification ×400. Middle, quantification of NETs morphology with diffuse/spread NETs percentages, Right, CitH3 concentrations, SNHG16, TRAF6 and miR-146a levels in naïve mouse neutrophils stimulated with various concentrations of HMGB1 or 3 μg/mL LPS. Values are mean ± SD. 4 mice per group in **a**. All results in Fig. S9 were representative of 2 independent experiments with similar findings. **p < 0.01, ***p < 0.001. **Fig. S10.** TLR4 expression in SNHG16-overexpressed/silenced HL-60 cells and reversed reduction in NETosis in miR‑146a‑silenced CasRX-SNHG16-transfected HL‑60 cells. **a** TLR4 mRNA levels in SNHG16-overexpressed (left) and -silenced HL-60 cells (right). **b** Representative immunoblot assay of TLR4 levels in SNHG16-overexpressed (left) and -silenced HL-60 cells (right). **c** MiR-17 and miR-146a levels in SNHG16-overexpressed and -silenced HL-60 cells. **d** Left, miR-146a levels in miR‑146a‑silenced CasRX-SNHG16-transfected HL‑60 cells. Right, quantification of NETs formation percentages (left), CitH3 levels (middle), and PAD4 levels (right) in miR‑146a-silenced CasRx‑SNGH16‑transfected dHL‑60 transfectants stimulated with 500 ng/mL LPS for 4 h. Values are mean ± SD. Results in Fig. S10**a** to S10**c** were representative of 3 independent experiments, and in Fig. S10**d** were representative of 2 independent experiments with similar findings. *p < 0.05, **p < 0.01, ***p < 0.001. **Fig. S11.** ATG5 expression in LPS-stimulated MLE-12 cells and lung tissues from pristane-induced AH mice.** a** ATG5 levels in MLE-12 cells under the stimulation of different LPS concentrations for 4 h (left) and 50 µg/mL LPS for different time (right). **b** ATG5 levels in lung tissues from pristane-induced or saline-injected mice. **c** ATG5 levels in lung tissues from pristane-induced mice receiving SFFV/SFFV-SNHG16 or sh-luciferase/sh-SNHG16 intra-pulmonary delivery. Values are mean ± SD. 5 mice per group in **b**. 8 mice per group in **c**. Results in Fig. S11**a** were representative of 3 independent experiments, and in Fig. S11**b** and S11**c** were representative of 2 independent experiments with similar findings. *p < 0.05, **p < 0.01, ***p < 0.001. **Fig. S12.** SNHG16 and NEAT1 expression in PBMCs, USCs and biopsied renal tissues from LN patients. **a** SNHG16 and **d** NEAT1 levels in PBMCs from HC, Nil and LN patients. **b** SNHG16 and **e** NEAT1 levels in USCs from HC, Nil and LN patients. **c** SNHG16 and **f** NEAT1 levels in kidney tissues from control and LN. Values are mean ± SD. Horizontal lines are mean values. Patient numbers, for PBMC and USC, n = 15 for Nil, n = 15 for LN, for renal tissue, n = 5 for control, n = 5 for LN. *p < 0.05, ***p < 0.001. **Fig. S13.** SNHG16 expression in a mouse LN model. **a** Serial measurement of proteinuria levels in mice at month 0, 1, 3, 5 and 6. Serial measurement of anti‑RNP. **b** and anti-dsDNA titers. **c** at month 0, 1, 3, 5 and 6.** d** Periodic acid-Schiff staining of renal glomeruli at month 6 after saline (left) or pristane injection (right). Arrows indicating normal glomeruli (left) or a glomerulus with GN formation (right). Scale bar = 10 µm, magnification ×400.** e** Kinetic expression of SNHG16 in the kidneys from saline- and pristane-injected mice at month 0, 1, 3, 5 and 6. Values are mean ± SD. All results in Fig. S13 were representative of 2 independent experiments with similar findings. 5 mice per group in Fig. S11. *p < 0.05, **p < 0.01, ***p < 0.001.

**Additional file 2: Table S1.** Clinical profiles and therapeutic modality of AH in different patient groups. **Table S2.** Linear regression for SNHG16, TLR4 and TRAF6 levels in PBMCs from SLE and HC. **Table S3.** Linear regression for SNHG16, TLR4 and TRAF6 levels in PBNs from SLE and HC.

## Data Availability

The datasets used and/or analyzed in the current study are available from the corresponding author on reasonable request.

## Abbreviations

AAV: ANCA-associated vasculitis
AH: Alveolar hemorrhage
ANCA: Anti-neutrophil cytoplasmic antibody
APS: Anti-phospholipid syndrome
ATCC: American Type Culture Collection
cDNA: Complementary DNA
ceRNA: Competing endogenous RNA
CitH3: Citrullinated Histone 3
CRISPR: Clustered regularly interspaced short palindromic repeat
DAMP: Damage-associated molecular pattern
DMSO: Dimethyl sulfoxide
dHL-60: Differentiated HL-60
Dox: Doxorubicin
ELISA: Enzyme-linked immunosorbent assay
GFP: Green fluorescence protein
GN: Glomerulonephritis
Ig: Immunoglobulin
HC: Healthy control
Hb: Hemoglobulin, Hct: Hematocrit
H&E: Hematoxylin and eosin
HMGB1: High-mobility group box 1
IC: Immune complex
IFN: Interferon
IgAV: IgA vasculitis
LN: Lupus nephritis
LncRNA: Long noncoding RNA
LPS: Lipopolysaccharide
LV: Lentivirus
MiRNA: MicroRNA
MRE: MiRNA recognition element
mTOR: Mammalian target of rapamycin
NC: Negative control
NET: Neutrophil extracellular trap
PAD4: Protein arginine deiminase 4
PB: Peripheral blood
PBM: Peripheral blood monocyte
PBN: Peripheral blood neutrophil
PBMC: Peripheral blood mononuclear cells
PBS: Phosphate-buffered saline
PC: Positive control
PTX: Pneumothorax
qRT-PCR: Quantitative real time polymerase chain reaction
RBC: Red blood cells
RTX: Rituximab
ROS: Reactive oxygen species
RT: Room temperature
SARS-CoV-2: Severe acute respiratory syndrome-coronavirus-2
SD: Standard deviation
shRNA: Short hairpin RNA
siRNA: Small interfering RNA
SLE: Systemic lupus erythematosus
SLEDAI-2 K: SLE disease activity index 2000
SNHG: Small nucleolar RNA host gene
TLR4: Toll-like receptor 4
Tm: Melting temperature
TU: Transduction unit
TUNEL: Terminal deoxynucleotidyl transferase dUTP nick end labeling
USC: Urine sediment cell

## References

[1] Tsokos GC, Lo MS, Costa Reis P, Sullivan KE (2016). New insights into the immunopathogenesis of systemic lupus erythematosus. Nat Rev Rheumatol.

[2] Liu MF, Wang CR, Fung LL (2004). Decreased CD4-positive CD25-positive T cells in peripheral blood of patients with systemic lupus erythematosus. Scand J Immunol.

[3] Mistry P, Kaplan MJ (2017). Cell death in the pathogenesis of systemic lupus erythematosus and lupus nephritis. Clin Immunol.

[4] Yu F, Haas M, Glassock R, Zhao MH (2017). Redefining lupus nephritis: clinical implications of pathophysiologic subtypes. Nat Rev Nephrol.

[5] Wang CR, Lin WC, Liu MF, Brown RM (2021). Pulmonary capillaritis in systemic lupus erythematosus. Vasculitis from diagnosis to treatment.

[6] Zhang P, Wu W, Chen Q, Chen M (2019). Non-coding RNAs and their integrated networks. J Integr Bioinform.

[7] Pu M, Chen J, Tao Z, Miao L, Qi X, Wang Y (2019). Regulatory network of miRNA on its target: coordination between transcriptional and post-transcriptional regulation of gene expression. Cell Mol Life Sci.

[8] Jovanovic M, Hengartner MO (2006). MiRNAs and apoptosis: RNAs to die for. Oncogene.

[9] Águila S, de los Reyes-García AM, Fernández-Pérez MP, Reguilón-Gallego L, Zapata-Martínez L, Ruiz-Lorente I (2021). MicroRNAs as new regulators of neutrophil extracellular trap formation. Int J Mol Sci.

[10] Ghafouri-Fard S, Shoorei H, Mohaqiq M, Majidpoor J, Moosavi MA, Taheri M (2022). Exploring the role of non-coding RNAs in autophagy. Autophagy.

[11] Ransohoff JD, Wei Y, Khavari PA (2018). The functions and unique features of long intergenic non-coding RNA. Nat Rev Mol Cell Biol.

[12] Yang C, Wu D, Gao L, Liu X, Jin Y, Wang D (2016). Competing endogenous RNA networks in human cancer: hypothesis, validation, and perspectives. Oncotarget.

[13] Lin W, Liu H, Tang Y, Wei Y, Wei W, Zhang L (2021). The development and controversy of competitive endogenous RNA hypothesis in non-coding genes. Mol Cell Biochem.

[14] Winkle M, El-Daly SM, Fabbri M, Calin GA (2021). Noncoding RNA therapeutics- challenges and potential solutions. Nat Rev Drug Discov.

[15] Chen YC, Chou YC, Hsieh YT, Kuo PY, Yang ML, Chong HE (2021). Targeting intra-pulmonary p53-dependent long non-coding RNA expression as a therapeutic intervention for systemic lupus erythematosus-associated diffuse alveolar hemorrhage. Int J Mol Sci.

[16] Williams GT, Farzaneh F (2012). Are snoRNAs and snoRNA host genes new players in cancer?. Nat Rev Cancer.

[17] Zimta A-A, Tigu AB, Braicu C, Stefan C, Ionescu C, Berindan-Neagoe I (2020). An emerging class of long non-coding RNA with oncogenic role arises from the snoRNA host genes. Front Oncol.

[18] Mishra S, Shah MI, Sarkar M, Rout C (2018). Integrated analysis of non-coding RNAs for the identification of promising biomarkers in interstitial lung diseases. Gene Reports.

[19] Zhou Z, Zhu Y, Gao G, Zhang Y (2019). Long noncoding RNA SNHG16 targets miR-146a-5p/CCL5 to regulate LPS-induced WI-38 cell apoptosis and inflammation in acute pneumonia. Life Sci.

[20] Zhang J, Mao F, Zhao G, Wang H, Yan X, Zhang Q (2020). Long non-coding RNA SNHG16 promotes lipopolysaccharides-induced acute pneumonia in A549 cells via targeting miR-370-3p/IGF2 axis. Int Immunopharmacol.

[21] Liu P, Zhao L, Gu Y, Zhang M, Gao H, Meng Y (2021). LncRNA SNHG16 promotes pulmonary fibrosis by targeting miR-455-3p to regulate the Notch2 pathway. Respir Res.

[22] He L, Wu X, Siegel R, Lipsky PE (2006). TRAF6 regulates cell fate decisions by inducing caspase 8-dependent apoptosis and the activation of NF-kappaB. J Biol Chem.

[23] Zhang K, Huang Q, Deng S, Yang Y, Li J, Wang S (2021). Mechanisms of TLR4-mediated autophagy and nitroxidative stress. Front Cell Infect Microbiol.

[24] Hsieh YT, Chou YC, Kuo PY, Tsai HW, Yen YT, Shiau AL (2022). Down-regulated miR-146a expression with increased neutrophil extracellular traps and apoptosis formation in autoimmune-mediated diffuse alveolar hemorrhage. J Biomed Sci.

[25] Alexander C, Rietschel ET (2001). Bacterial lipopolysaccharides and innate immunity. J Endotoxin Res.

[26] Xia L, Zhu G, Huang H, He Y, Liu X (2021). LncRNA small nucleolar RNA host gene 16 (SNHG16) silencing protects lipopolysaccharide (LPS)-induced cell injury in human lung fibroblasts WI-38 through acting as miR-141-3p sponge. Biosci Biotechnol Biochem.

[27] Wang W, Lou C, Gao J, Zhang X, Du Y (2018). LncRNA SNHG16 reverses the effects of miR-15a/16 on LPS-induced inflammatory pathway. Biomed Pharmacother.

[28] Liu Y, Zhang M, Zhong H, Xie N, Wang Y, Ding S (2023). LncRNA SNHG16 regulates RAS and NF-κB pathway-mediated NLRP3 inflammasome activation to aggravate diabetes nephropathy through stabilizing TLR4. Acta Diabetol.

[29] Yang K, He YS, Wang XQ, Lu L, Chen QJ, Liu J (2011). MiR-146a inhibits oxidized low-density lipoprotein-induced lipid accumulation and inflammatory response via targeting toll-like receptor 4. FEBS Lett.

[30] Zhang F, Wu L, Qian J, Qu B, Xia S, La T (2016). Identification of the long noncoding RNA NEAT1 as a novel inflammatory regulator acting through MAPK pathway in human lupus. J Autoimmun.

[31] Hochberg MC (1997). Updating the American College of Rheumatology revised criteria for the classification of systemic lupus erythematosus. Arthritis Rheum.

[32] Gladman DD, Ibañez D, Urowitz MB (2002). Systemic lupus erythematosus disease activity index 2000. J Rheumatol.

[33] Wang CR, Liu MF, Weng CT, Lin WC, Li WT, Tsai HW (2018). Systemic lupus erythematosus-associated diffuse alveolar hemorrhage: A monocentric experience in Han Chinese patients. Scand J Rheumatol.

[34] Peng JS, Chen SY, Wu CL, Chong HE, Ding YC, Shiau AL (2016). Amelioration of experimental autoimmune arthritis through targeting synovial fibroblasts by the intra-articular delivery of microRNA-140-3p and -5p. Arthritis Rheumatol.

[35] Chen YC, Kuo PY, Chou YC, Chong HE, Hsieh YT, Yang ML (2021). Up-Regulated expression of pro-apoptotic long noncoding RNA lincRNA-p21 with enhanced cell apoptosis in lupus nephritis. Int J Mol Sci.

[36] Kessenbrock K, Krumbholz M, Schönermarck U, Back W, Gross WL, Werb Z (2009). Netting neutrophils in autoimmune small-vessel vasculitis. Nat Med.

[37] Wu ZZ, Zhang JJ, Gao CC, Zhao M, Liu SY, Gao GM (2017). Expression of autophagy related genes mTOR, Beclin-1, LC3 and p62 in the peripheral blood mononuclear cells of systemic lupus erythematosus. Am J Clin Exp Immunol.

[38] Ghafouri-Fard S, Khoshbakht T, Taheri M, Shojaei S (2021). A review on the role of small nucleolar RNA host gene 16 long non-coding RNAs in the carcinogenic processes. Front Cell Dev Biol.

[39] Dos Santos M, de Souza Silva JM, Bartikoski BJ, Freitas EC, Busatto A, do EspíritoSanto RC (2022). Vitamin D supplementation modulates autophagy in the pristane-induced lupus model. Adv Rheumatol.

[40] Wen Y, Gong X, Dong Y, Tang C (2020). Long noncoding RNA SNHG16 facilitates proliferation, migration, invasion and autophagy of neuroblastoma cells via sponging miR-542-3p and upregulating ATG5 expression. Onco Targets Ther.

[41] Kma L, Baruah TJ (2022). The interplay of ROS and the PI3K/Akt pathway in autophagy regulation. Biotechnol Appl Biochem.

[42] Tang D, Kang R, Berghe TV, Vandenabeele P, Kroemer G (2019). The molecular machinery of regulated cell death. Cell Res.

[43] Bedoui S, Herold MJ, Strasser A (2020). Emerging connectivity of programmed cell death pathways and its physiological implications. Nat Rev Mol Cell Biol.

[44] Chen T, Tu S, Ding L, Jin M, Chen H, Zhou H (2023). The role of autophagy in viral infections. J Biomed Sci.

[45] Lee KH, Kronbichler A, Park DD, Park Y, Moon H, Kim H (2017). Neutrophil extracellular traps (NETs) in autoimmune diseases: a comprehensive review. Autoimmun Rev.

[46] Qi YY, Zhou XJ, Zhang H (2019). Autophagy and immunological aberrations in systemic lupus erythematosus. Eur J Immunol.

[47] Audemard-Verger A, Pillebout E, Guillevin L, Thervet E, Terrier B (2015). IgA vasculitis (Henoch-Shönlein purpura) in adults: diagnostic and therapeutic aspects. Autoimmun Rev.

[48] Idborg H, Eketjäll S, Pettersson S, Gustafsson JT, Zickert A, Kvarnström M (2018). TNF-α and plasma albumin as biomarkers of disease activity in systemic lupus erythematosus. Lupus Sci Med.

[49] Taheri M, Eghtedarian R, Dinger ME, Ghafouri-Fard S (2020). Exploring the role of non-coding RNAs in the pathophysiology of systemic lupus erythematosus. Biomolecules.

[50] Hadjicharalambous MR, Lindsay MA (2019). Long non-coding RNAs and the innate immune response. Noncoding RNA.

[51] Devarapu SK, Anders HJ (2018). Toll-like receptors in lupus nephritis. J Biomed Sci.

[52] Liu T, Son M, Diamond B (2020). HMGB1 in systemic lupus erythematosus. Front Immunol.

[53] Summers SA, Hoi A, Steinmetz OM, O’Sullivan KM, Ooi JD, Odobasic D (2010). TLR9 and TLR4 are required for the development of autoimmunity and lupus nephritis in pristane nephropathy. J Autoimmun.

[54] Liu B, Yang Y, Dai J, Medzhitov R, Freudenberg MA, Zhang PL (2006). TLR4 up-regulation at protein or gene level is pathogenic for lupus-like autoimmune disease. J Immunol.

[55] Huang Q, Shen S, Qu H, Huang Y, Wu D, Jiang H (2020). Expression of HMGB1 and TLR4 in neuropsychiatric systemic lupus erythematosus patients with seizure disorders. Ann Transl Med.

[56] Calvani N, Caricchio R, Tucci M, Sobel ES, Silvestris F, Tartaglia P (2005). Induction of apoptosis by the hydrocarbon oil pristane: Implications for pristane-induced lupus. J Immunol.

[57] Barker TT, Lee PY, Kelly-Scumpia KM, Weinstein JS, Nacionales DC, Kumagai Y (2011). Pathogenic role of B cells in the development of diffuse alveolar hemorrhage induced by pristane. Lab Invest.

[58] Al-Adhoubi NK, Bystrom J (2020). Systemic lupus erythematosus and diffuse alveolar hemorrhage, etiology and novel treatment strategies. Lupus.

[59] Mokrzycki MH, Balogun RA (2011). Therapeutic apheresis: a review of complications and recommendations for prevention and management. J Clin Apher.

[60] Park JA (2021). Treatment of diffuse alveolar hemorrhage: controlling inflammation and obtaining rapid and effective hemostasis. Int J Mol Sci.

[61] Wang CR, Lin WC (2023). Severe COVID-19 pneumonia in patients with rheumatoid arthritis under B-cell depletion therapy. J Formos Med Assoc.

[62] Mehta P, Gasparyan AY, Zimba O, Kitas GD (2022). Systemic lupus erythematosus in the light of the COVID-19 pandemic: infection, vaccination, and impact on disease management. Clin Rheumatol.

[63] Mutua V, Gershwin LJ (2021). A review of neutrophil extracellular traps (NETs) in disease: potential anti-NETs therapeutics. Clin Rev Allergy Immunol.

[64] Demkow U (2023). Molecular mechanisms of neutrophil extracellular trap (NETs) degradation. Int J Mol Sci.

[65] Heslet L, Nielsen JD, Nepper-Christensen S (2012). Local pulmonary administration of factor VIIa (rFVIIa) in diffuse alveolar hemorrhage (DAH)- A review of a new treatment paradigm. Biologics.

[66] Diaz R, Almeida P, Alvarez M, Ferrer G, Hernandez F (2019). Life-threatening pulmonary hemorrhage responds to recombinant factor VIIa: a case series in south Florida hospitals. Cureus.

[67] Curreri A, Sankholkar D, Mitragotri S, Zhao Z (2022). RNA therapeutics in the clinic. Bioeng Transl Med.

[68] Yang H, Liu Y, Chen L, Zhao J, Guo M, Zhao X (2023). MiRNA-based therapies for lung cancer: opportunities and challenges?. Biomolecules.

