# Supplementary figures and images for: Long noncoding RNA SNHG16 regulates TLR4-mediated autophagy and NETosis formation in alveolar hemorrhage associated with systemic lupus erythematosus
Source: J Biomed Sci. 2023 Sep 12;30:78. doi: 10.1186/s12929-023-00969-5 (PMC10496234; doi:10.1186/s12929-023-00969-5)

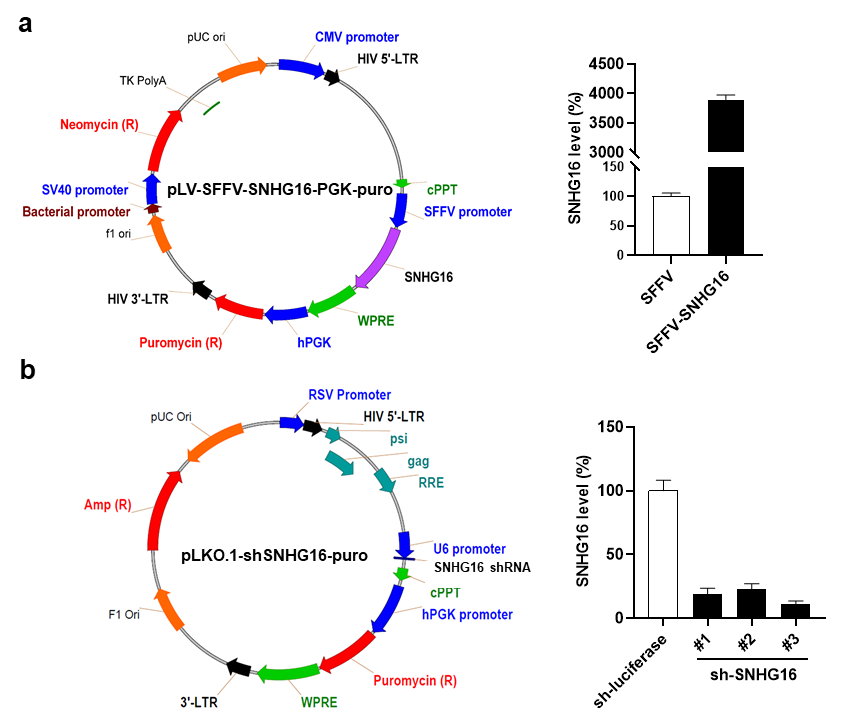

Supplement: Supplementary file 1 — Additional file 1: Fig. S1. SNHG16 expression in LV-SNHG16‑ and sh-SNHG16-transfected cells. a Left, map of pLV-SFFV-SNHG16-PGK-puro, a total of 9.3 kb in length. Right, SNHG16 levels in LV-SNHG16‑transfected 293T cells. b Left, map of pLKO.1-sh-SNHG16-puro, a total of 7.4 kb in length. Right, SNHG16 levels in sh-SNHG16‑transfected MLE-12 cells. Values are mean ± SD. Results in Fig. S1a were representative of 3 independent experiments, and in Fig. S1b were representative of 2 independent experiments with similar findings. Fig. S2. Expression of SNHG16 in purified neutrophils and sorted T cells, monocytes and B cells from PB of healthy individuals. a Flow cytometric graphs of sorted CD3-positive T cells, CD14-positive monocytes and CD19-positive B cells from healthy individual No. 1, No. 2 and No. 3. b PB cell numbers/µL (left) and SNHG16 levels/105 cells (right) in neutrophils, T cells, monocytes and B cells from healthy individual No. 1, No. 2 and No. 3. Values are mean ± SD. Fig. S3. Expression of miR-146a in PBMCs and PBNs from SLE patients. a MiR-146a levels in PBMCs from SLE patients and HC. b A negative correlation between miR-146a levels in PBMCs and SLEDAI-2K activity scores. c MiR-146a levels in PBMCs from HC, Nil. LN, SLE-AH and other AH patients. A negative correlation between miR-146a and d TLR4, e TRAF6 and f NEAT1 levels in PBMCs from SLE patients. g MiR-146a levels in PBNs from SLE patients and HC. h A negative correlation between miR-146a levels in PBNs and SLEDAI-2K activity scores. i MiR-146a levels in PBNs from HC, Nil. LN and AH patients. Values are mean ± SD. Horizontal lines are mean values. Patient numbers, n = 62 for PBMCs from SLE, 15 for PBNs from SLE, 7 for PBMCs from Nil, LN and SLE-AH, 6 for PBMCs from other AH, 5 for PBNs from Nil, LN, 4 for PBN from AH. *p < 0.05. **p < 0.01, ***p < 0.001. Fig. S4. Expression of NEAT1 in SLE patients and pristane-injected mice. NEAT1 levels in a PBMCs and e PBNs from SLE patients and HC. b A positive c [file 12929_2023_969_MOESM1_ESM.zip › New Revised Suppl Fig 1.tif]

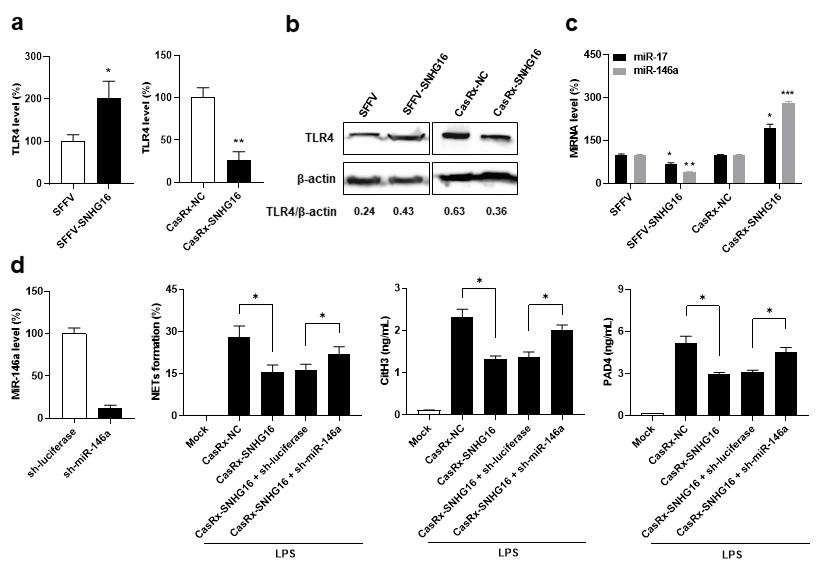

Supplement: Supplementary file 1 — Additional file 1: Fig. S1. SNHG16 expression in LV-SNHG16‑ and sh-SNHG16-transfected cells. a Left, map of pLV-SFFV-SNHG16-PGK-puro, a total of 9.3 kb in length. Right, SNHG16 levels in LV-SNHG16‑transfected 293T cells. b Left, map of pLKO.1-sh-SNHG16-puro, a total of 7.4 kb in length. Right, SNHG16 levels in sh-SNHG16‑transfected MLE-12 cells. Values are mean ± SD. Results in Fig. S1a were representative of 3 independent experiments, and in Fig. S1b were representative of 2 independent experiments with similar findings. Fig. S2. Expression of SNHG16 in purified neutrophils and sorted T cells, monocytes and B cells from PB of healthy individuals. a Flow cytometric graphs of sorted CD3-positive T cells, CD14-positive monocytes and CD19-positive B cells from healthy individual No. 1, No. 2 and No. 3. b PB cell numbers/µL (left) and SNHG16 levels/105 cells (right) in neutrophils, T cells, monocytes and B cells from healthy individual No. 1, No. 2 and No. 3. Values are mean ± SD. Fig. S3. Expression of miR-146a in PBMCs and PBNs from SLE patients. a MiR-146a levels in PBMCs from SLE patients and HC. b A negative correlation between miR-146a levels in PBMCs and SLEDAI-2K activity scores. c MiR-146a levels in PBMCs from HC, Nil. LN, SLE-AH and other AH patients. A negative correlation between miR-146a and d TLR4, e TRAF6 and f NEAT1 levels in PBMCs from SLE patients. g MiR-146a levels in PBNs from SLE patients and HC. h A negative correlation between miR-146a levels in PBNs and SLEDAI-2K activity scores. i MiR-146a levels in PBNs from HC, Nil. LN and AH patients. Values are mean ± SD. Horizontal lines are mean values. Patient numbers, n = 62 for PBMCs from SLE, 15 for PBNs from SLE, 7 for PBMCs from Nil, LN and SLE-AH, 6 for PBMCs from other AH, 5 for PBNs from Nil, LN, 4 for PBN from AH. *p < 0.05. **p < 0.01, ***p < 0.001. Fig. S4. Expression of NEAT1 in SLE patients and pristane-injected mice. NEAT1 levels in a PBMCs and e PBNs from SLE patients and HC. b A positive c [file 12929_2023_969_MOESM1_ESM.zip › New Revised Suppl Fig 10.tif]

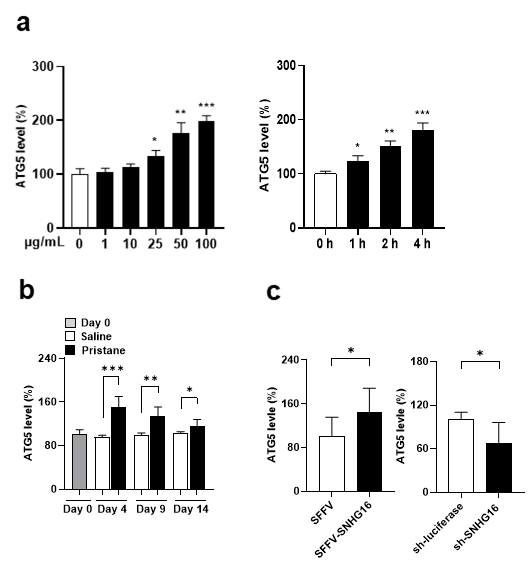

Supplement: Supplementary file 1 — Additional file 1: Fig. S1. SNHG16 expression in LV-SNHG16‑ and sh-SNHG16-transfected cells. a Left, map of pLV-SFFV-SNHG16-PGK-puro, a total of 9.3 kb in length. Right, SNHG16 levels in LV-SNHG16‑transfected 293T cells. b Left, map of pLKO.1-sh-SNHG16-puro, a total of 7.4 kb in length. Right, SNHG16 levels in sh-SNHG16‑transfected MLE-12 cells. Values are mean ± SD. Results in Fig. S1a were representative of 3 independent experiments, and in Fig. S1b were representative of 2 independent experiments with similar findings. Fig. S2. Expression of SNHG16 in purified neutrophils and sorted T cells, monocytes and B cells from PB of healthy individuals. a Flow cytometric graphs of sorted CD3-positive T cells, CD14-positive monocytes and CD19-positive B cells from healthy individual No. 1, No. 2 and No. 3. b PB cell numbers/µL (left) and SNHG16 levels/105 cells (right) in neutrophils, T cells, monocytes and B cells from healthy individual No. 1, No. 2 and No. 3. Values are mean ± SD. Fig. S3. Expression of miR-146a in PBMCs and PBNs from SLE patients. a MiR-146a levels in PBMCs from SLE patients and HC. b A negative correlation between miR-146a levels in PBMCs and SLEDAI-2K activity scores. c MiR-146a levels in PBMCs from HC, Nil. LN, SLE-AH and other AH patients. A negative correlation between miR-146a and d TLR4, e TRAF6 and f NEAT1 levels in PBMCs from SLE patients. g MiR-146a levels in PBNs from SLE patients and HC. h A negative correlation between miR-146a levels in PBNs and SLEDAI-2K activity scores. i MiR-146a levels in PBNs from HC, Nil. LN and AH patients. Values are mean ± SD. Horizontal lines are mean values. Patient numbers, n = 62 for PBMCs from SLE, 15 for PBNs from SLE, 7 for PBMCs from Nil, LN and SLE-AH, 6 for PBMCs from other AH, 5 for PBNs from Nil, LN, 4 for PBN from AH. *p < 0.05. **p < 0.01, ***p < 0.001. Fig. S4. Expression of NEAT1 in SLE patients and pristane-injected mice. NEAT1 levels in a PBMCs and e PBNs from SLE patients and HC. b A positive c [file 12929_2023_969_MOESM1_ESM.zip › New Revised Suppl Fig 11.tif]

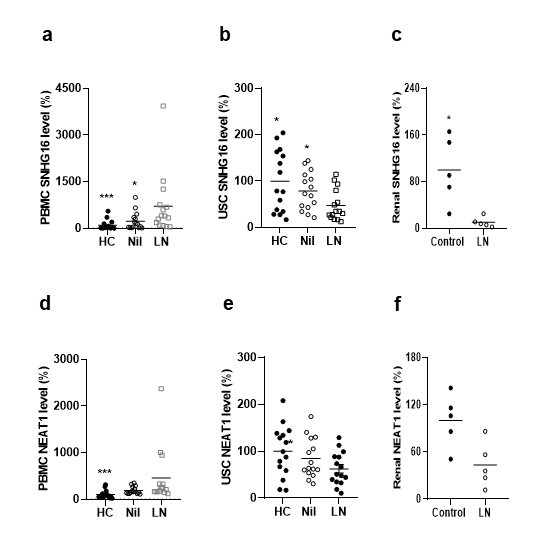

Supplement: Supplementary file 1 — Additional file 1: Fig. S1. SNHG16 expression in LV-SNHG16‑ and sh-SNHG16-transfected cells. a Left, map of pLV-SFFV-SNHG16-PGK-puro, a total of 9.3 kb in length. Right, SNHG16 levels in LV-SNHG16‑transfected 293T cells. b Left, map of pLKO.1-sh-SNHG16-puro, a total of 7.4 kb in length. Right, SNHG16 levels in sh-SNHG16‑transfected MLE-12 cells. Values are mean ± SD. Results in Fig. S1a were representative of 3 independent experiments, and in Fig. S1b were representative of 2 independent experiments with similar findings. Fig. S2. Expression of SNHG16 in purified neutrophils and sorted T cells, monocytes and B cells from PB of healthy individuals. a Flow cytometric graphs of sorted CD3-positive T cells, CD14-positive monocytes and CD19-positive B cells from healthy individual No. 1, No. 2 and No. 3. b PB cell numbers/µL (left) and SNHG16 levels/105 cells (right) in neutrophils, T cells, monocytes and B cells from healthy individual No. 1, No. 2 and No. 3. Values are mean ± SD. Fig. S3. Expression of miR-146a in PBMCs and PBNs from SLE patients. a MiR-146a levels in PBMCs from SLE patients and HC. b A negative correlation between miR-146a levels in PBMCs and SLEDAI-2K activity scores. c MiR-146a levels in PBMCs from HC, Nil. LN, SLE-AH and other AH patients. A negative correlation between miR-146a and d TLR4, e TRAF6 and f NEAT1 levels in PBMCs from SLE patients. g MiR-146a levels in PBNs from SLE patients and HC. h A negative correlation between miR-146a levels in PBNs and SLEDAI-2K activity scores. i MiR-146a levels in PBNs from HC, Nil. LN and AH patients. Values are mean ± SD. Horizontal lines are mean values. Patient numbers, n = 62 for PBMCs from SLE, 15 for PBNs from SLE, 7 for PBMCs from Nil, LN and SLE-AH, 6 for PBMCs from other AH, 5 for PBNs from Nil, LN, 4 for PBN from AH. *p < 0.05. **p < 0.01, ***p < 0.001. Fig. S4. Expression of NEAT1 in SLE patients and pristane-injected mice. NEAT1 levels in a PBMCs and e PBNs from SLE patients and HC. b A positive c [file 12929_2023_969_MOESM1_ESM.zip › New Revised Suppl Fig 12.tif]

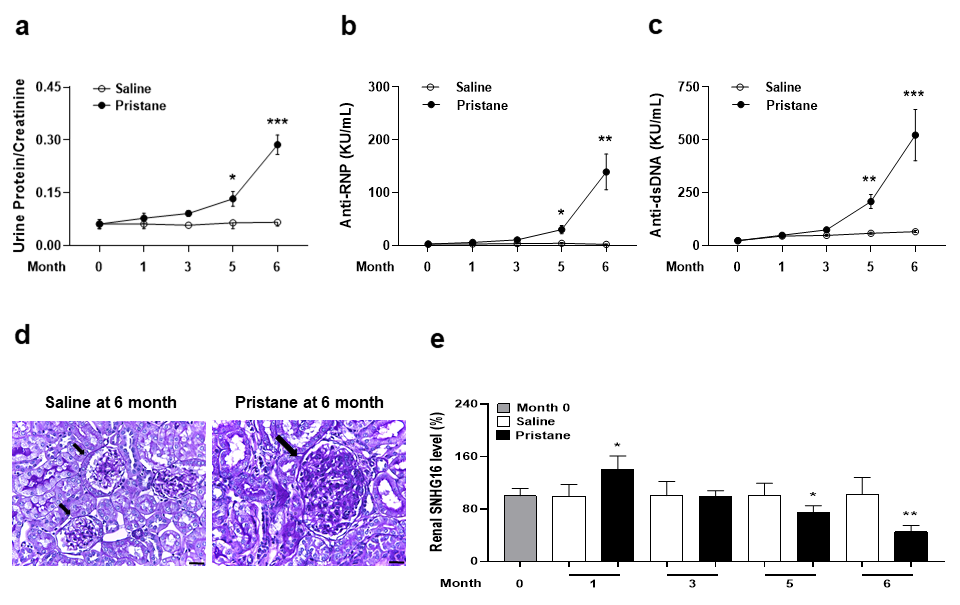

Supplement: Supplementary file 1 — Additional file 1: Fig. S1. SNHG16 expression in LV-SNHG16‑ and sh-SNHG16-transfected cells. a Left, map of pLV-SFFV-SNHG16-PGK-puro, a total of 9.3 kb in length. Right, SNHG16 levels in LV-SNHG16‑transfected 293T cells. b Left, map of pLKO.1-sh-SNHG16-puro, a total of 7.4 kb in length. Right, SNHG16 levels in sh-SNHG16‑transfected MLE-12 cells. Values are mean ± SD. Results in Fig. S1a were representative of 3 independent experiments, and in Fig. S1b were representative of 2 independent experiments with similar findings. Fig. S2. Expression of SNHG16 in purified neutrophils and sorted T cells, monocytes and B cells from PB of healthy individuals. a Flow cytometric graphs of sorted CD3-positive T cells, CD14-positive monocytes and CD19-positive B cells from healthy individual No. 1, No. 2 and No. 3. b PB cell numbers/µL (left) and SNHG16 levels/105 cells (right) in neutrophils, T cells, monocytes and B cells from healthy individual No. 1, No. 2 and No. 3. Values are mean ± SD. Fig. S3. Expression of miR-146a in PBMCs and PBNs from SLE patients. a MiR-146a levels in PBMCs from SLE patients and HC. b A negative correlation between miR-146a levels in PBMCs and SLEDAI-2K activity scores. c MiR-146a levels in PBMCs from HC, Nil. LN, SLE-AH and other AH patients. A negative correlation between miR-146a and d TLR4, e TRAF6 and f NEAT1 levels in PBMCs from SLE patients. g MiR-146a levels in PBNs from SLE patients and HC. h A negative correlation between miR-146a levels in PBNs and SLEDAI-2K activity scores. i MiR-146a levels in PBNs from HC, Nil. LN and AH patients. Values are mean ± SD. Horizontal lines are mean values. Patient numbers, n = 62 for PBMCs from SLE, 15 for PBNs from SLE, 7 for PBMCs from Nil, LN and SLE-AH, 6 for PBMCs from other AH, 5 for PBNs from Nil, LN, 4 for PBN from AH. *p < 0.05. **p < 0.01, ***p < 0.001. Fig. S4. Expression of NEAT1 in SLE patients and pristane-injected mice. NEAT1 levels in a PBMCs and e PBNs from SLE patients and HC. b A positive c [file 12929_2023_969_MOESM1_ESM.zip › New Revised Suppl Fig 13.tif]

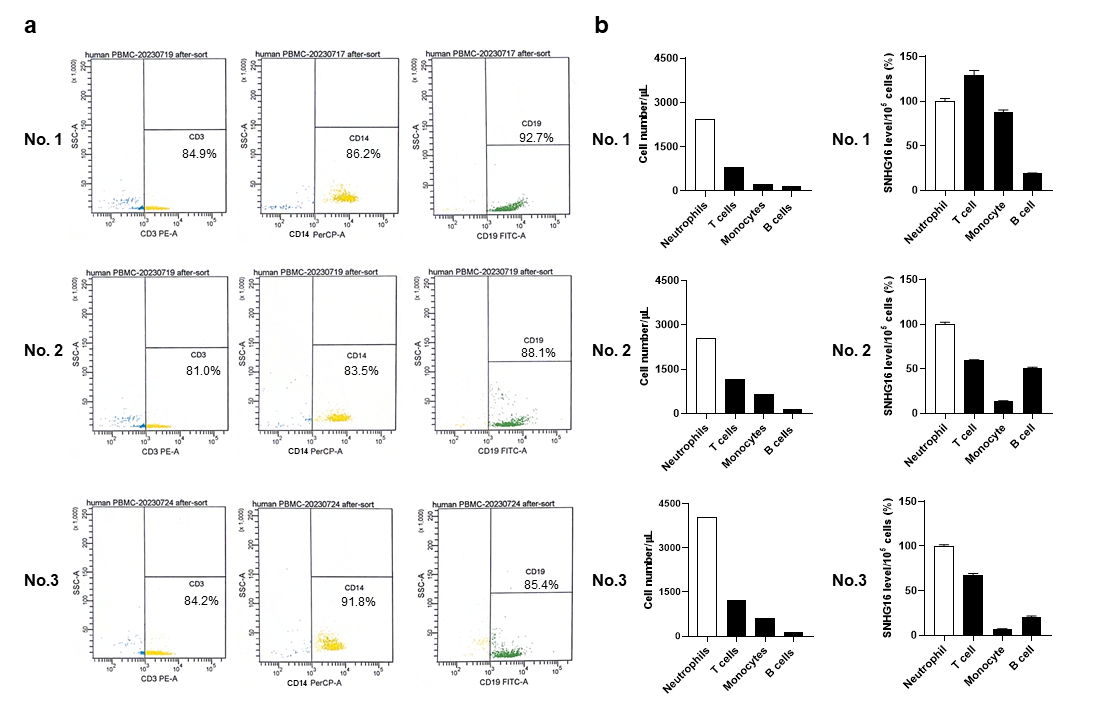

Supplement: Supplementary file 1 — Additional file 1: Fig. S1. SNHG16 expression in LV-SNHG16‑ and sh-SNHG16-transfected cells. a Left, map of pLV-SFFV-SNHG16-PGK-puro, a total of 9.3 kb in length. Right, SNHG16 levels in LV-SNHG16‑transfected 293T cells. b Left, map of pLKO.1-sh-SNHG16-puro, a total of 7.4 kb in length. Right, SNHG16 levels in sh-SNHG16‑transfected MLE-12 cells. Values are mean ± SD. Results in Fig. S1a were representative of 3 independent experiments, and in Fig. S1b were representative of 2 independent experiments with similar findings. Fig. S2. Expression of SNHG16 in purified neutrophils and sorted T cells, monocytes and B cells from PB of healthy individuals. a Flow cytometric graphs of sorted CD3-positive T cells, CD14-positive monocytes and CD19-positive B cells from healthy individual No. 1, No. 2 and No. 3. b PB cell numbers/µL (left) and SNHG16 levels/105 cells (right) in neutrophils, T cells, monocytes and B cells from healthy individual No. 1, No. 2 and No. 3. Values are mean ± SD. Fig. S3. Expression of miR-146a in PBMCs and PBNs from SLE patients. a MiR-146a levels in PBMCs from SLE patients and HC. b A negative correlation between miR-146a levels in PBMCs and SLEDAI-2K activity scores. c MiR-146a levels in PBMCs from HC, Nil. LN, SLE-AH and other AH patients. A negative correlation between miR-146a and d TLR4, e TRAF6 and f NEAT1 levels in PBMCs from SLE patients. g MiR-146a levels in PBNs from SLE patients and HC. h A negative correlation between miR-146a levels in PBNs and SLEDAI-2K activity scores. i MiR-146a levels in PBNs from HC, Nil. LN and AH patients. Values are mean ± SD. Horizontal lines are mean values. Patient numbers, n = 62 for PBMCs from SLE, 15 for PBNs from SLE, 7 for PBMCs from Nil, LN and SLE-AH, 6 for PBMCs from other AH, 5 for PBNs from Nil, LN, 4 for PBN from AH. *p < 0.05. **p < 0.01, ***p < 0.001. Fig. S4. Expression of NEAT1 in SLE patients and pristane-injected mice. NEAT1 levels in a PBMCs and e PBNs from SLE patients and HC. b A positive c [file 12929_2023_969_MOESM1_ESM.zip › New Revised Suppl Fig 2.tif]

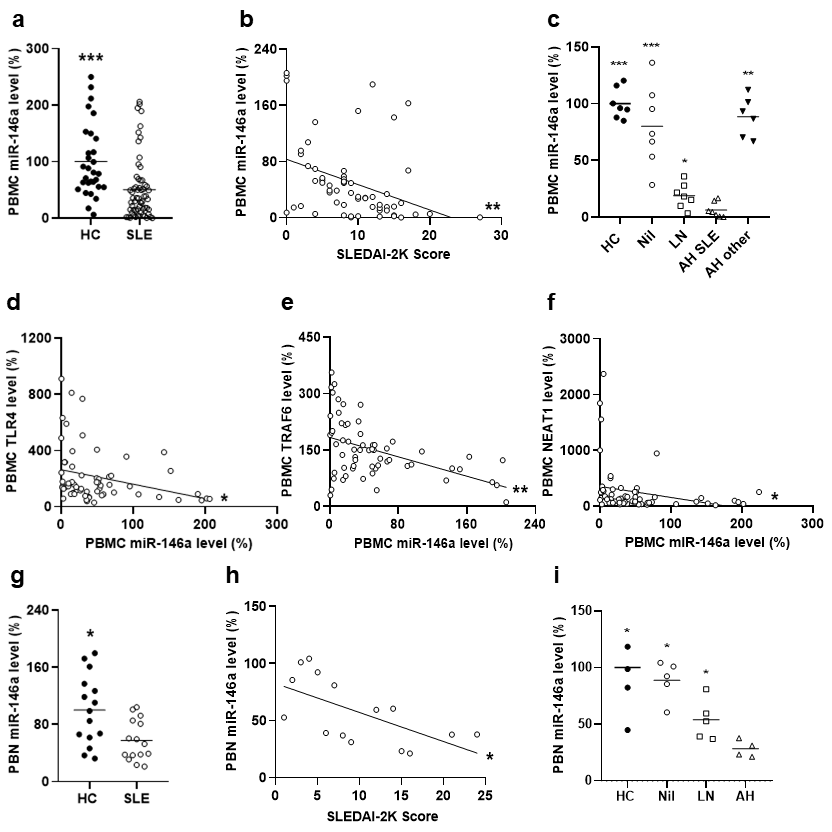

Supplement: Supplementary file 1 — Additional file 1: Fig. S1. SNHG16 expression in LV-SNHG16‑ and sh-SNHG16-transfected cells. a Left, map of pLV-SFFV-SNHG16-PGK-puro, a total of 9.3 kb in length. Right, SNHG16 levels in LV-SNHG16‑transfected 293T cells. b Left, map of pLKO.1-sh-SNHG16-puro, a total of 7.4 kb in length. Right, SNHG16 levels in sh-SNHG16‑transfected MLE-12 cells. Values are mean ± SD. Results in Fig. S1a were representative of 3 independent experiments, and in Fig. S1b were representative of 2 independent experiments with similar findings. Fig. S2. Expression of SNHG16 in purified neutrophils and sorted T cells, monocytes and B cells from PB of healthy individuals. a Flow cytometric graphs of sorted CD3-positive T cells, CD14-positive monocytes and CD19-positive B cells from healthy individual No. 1, No. 2 and No. 3. b PB cell numbers/µL (left) and SNHG16 levels/105 cells (right) in neutrophils, T cells, monocytes and B cells from healthy individual No. 1, No. 2 and No. 3. Values are mean ± SD. Fig. S3. Expression of miR-146a in PBMCs and PBNs from SLE patients. a MiR-146a levels in PBMCs from SLE patients and HC. b A negative correlation between miR-146a levels in PBMCs and SLEDAI-2K activity scores. c MiR-146a levels in PBMCs from HC, Nil. LN, SLE-AH and other AH patients. A negative correlation between miR-146a and d TLR4, e TRAF6 and f NEAT1 levels in PBMCs from SLE patients. g MiR-146a levels in PBNs from SLE patients and HC. h A negative correlation between miR-146a levels in PBNs and SLEDAI-2K activity scores. i MiR-146a levels in PBNs from HC, Nil. LN and AH patients. Values are mean ± SD. Horizontal lines are mean values. Patient numbers, n = 62 for PBMCs from SLE, 15 for PBNs from SLE, 7 for PBMCs from Nil, LN and SLE-AH, 6 for PBMCs from other AH, 5 for PBNs from Nil, LN, 4 for PBN from AH. *p < 0.05. **p < 0.01, ***p < 0.001. Fig. S4. Expression of NEAT1 in SLE patients and pristane-injected mice. NEAT1 levels in a PBMCs and e PBNs from SLE patients and HC. b A positive c [file 12929_2023_969_MOESM1_ESM.zip › New Revised Suppl Fig 3.tif]

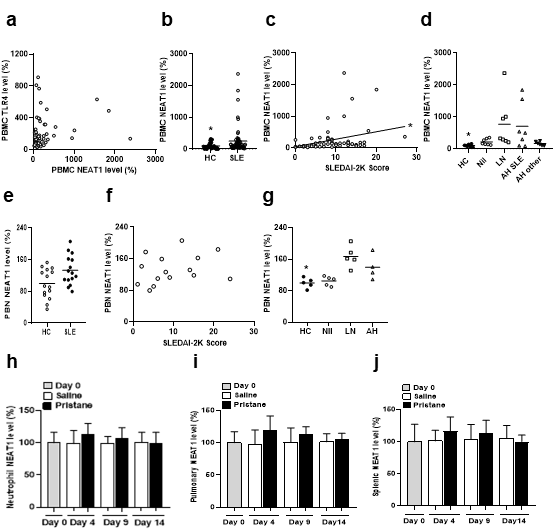

Supplement: Supplementary file 1 — Additional file 1: Fig. S1. SNHG16 expression in LV-SNHG16‑ and sh-SNHG16-transfected cells. a Left, map of pLV-SFFV-SNHG16-PGK-puro, a total of 9.3 kb in length. Right, SNHG16 levels in LV-SNHG16‑transfected 293T cells. b Left, map of pLKO.1-sh-SNHG16-puro, a total of 7.4 kb in length. Right, SNHG16 levels in sh-SNHG16‑transfected MLE-12 cells. Values are mean ± SD. Results in Fig. S1a were representative of 3 independent experiments, and in Fig. S1b were representative of 2 independent experiments with similar findings. Fig. S2. Expression of SNHG16 in purified neutrophils and sorted T cells, monocytes and B cells from PB of healthy individuals. a Flow cytometric graphs of sorted CD3-positive T cells, CD14-positive monocytes and CD19-positive B cells from healthy individual No. 1, No. 2 and No. 3. b PB cell numbers/µL (left) and SNHG16 levels/105 cells (right) in neutrophils, T cells, monocytes and B cells from healthy individual No. 1, No. 2 and No. 3. Values are mean ± SD. Fig. S3. Expression of miR-146a in PBMCs and PBNs from SLE patients. a MiR-146a levels in PBMCs from SLE patients and HC. b A negative correlation between miR-146a levels in PBMCs and SLEDAI-2K activity scores. c MiR-146a levels in PBMCs from HC, Nil. LN, SLE-AH and other AH patients. A negative correlation between miR-146a and d TLR4, e TRAF6 and f NEAT1 levels in PBMCs from SLE patients. g MiR-146a levels in PBNs from SLE patients and HC. h A negative correlation between miR-146a levels in PBNs and SLEDAI-2K activity scores. i MiR-146a levels in PBNs from HC, Nil. LN and AH patients. Values are mean ± SD. Horizontal lines are mean values. Patient numbers, n = 62 for PBMCs from SLE, 15 for PBNs from SLE, 7 for PBMCs from Nil, LN and SLE-AH, 6 for PBMCs from other AH, 5 for PBNs from Nil, LN, 4 for PBN from AH. *p < 0.05. **p < 0.01, ***p < 0.001. Fig. S4. Expression of NEAT1 in SLE patients and pristane-injected mice. NEAT1 levels in a PBMCs and e PBNs from SLE patients and HC. b A positive c [file 12929_2023_969_MOESM1_ESM.zip › New Revised Suppl Fig 4.tif]

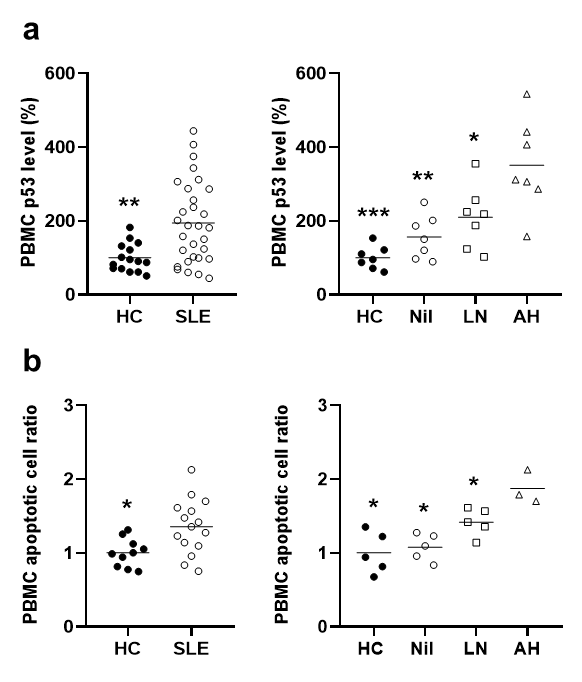

Supplement: Supplementary file 1 — Additional file 1: Fig. S1. SNHG16 expression in LV-SNHG16‑ and sh-SNHG16-transfected cells. a Left, map of pLV-SFFV-SNHG16-PGK-puro, a total of 9.3 kb in length. Right, SNHG16 levels in LV-SNHG16‑transfected 293T cells. b Left, map of pLKO.1-sh-SNHG16-puro, a total of 7.4 kb in length. Right, SNHG16 levels in sh-SNHG16‑transfected MLE-12 cells. Values are mean ± SD. Results in Fig. S1a were representative of 3 independent experiments, and in Fig. S1b were representative of 2 independent experiments with similar findings. Fig. S2. Expression of SNHG16 in purified neutrophils and sorted T cells, monocytes and B cells from PB of healthy individuals. a Flow cytometric graphs of sorted CD3-positive T cells, CD14-positive monocytes and CD19-positive B cells from healthy individual No. 1, No. 2 and No. 3. b PB cell numbers/µL (left) and SNHG16 levels/105 cells (right) in neutrophils, T cells, monocytes and B cells from healthy individual No. 1, No. 2 and No. 3. Values are mean ± SD. Fig. S3. Expression of miR-146a in PBMCs and PBNs from SLE patients. a MiR-146a levels in PBMCs from SLE patients and HC. b A negative correlation between miR-146a levels in PBMCs and SLEDAI-2K activity scores. c MiR-146a levels in PBMCs from HC, Nil. LN, SLE-AH and other AH patients. A negative correlation between miR-146a and d TLR4, e TRAF6 and f NEAT1 levels in PBMCs from SLE patients. g MiR-146a levels in PBNs from SLE patients and HC. h A negative correlation between miR-146a levels in PBNs and SLEDAI-2K activity scores. i MiR-146a levels in PBNs from HC, Nil. LN and AH patients. Values are mean ± SD. Horizontal lines are mean values. Patient numbers, n = 62 for PBMCs from SLE, 15 for PBNs from SLE, 7 for PBMCs from Nil, LN and SLE-AH, 6 for PBMCs from other AH, 5 for PBNs from Nil, LN, 4 for PBN from AH. *p < 0.05. **p < 0.01, ***p < 0.001. Fig. S4. Expression of NEAT1 in SLE patients and pristane-injected mice. NEAT1 levels in a PBMCs and e PBNs from SLE patients and HC. b A positive c [file 12929_2023_969_MOESM1_ESM.zip › New Revised Suppl Fig 5.tif]

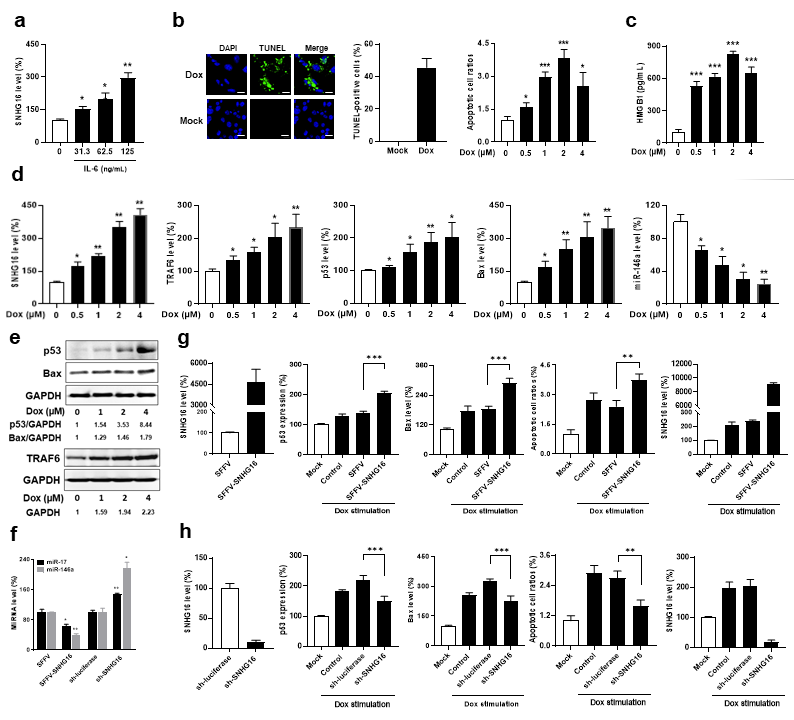

Supplement: Supplementary file 1 — Additional file 1: Fig. S1. SNHG16 expression in LV-SNHG16‑ and sh-SNHG16-transfected cells. a Left, map of pLV-SFFV-SNHG16-PGK-puro, a total of 9.3 kb in length. Right, SNHG16 levels in LV-SNHG16‑transfected 293T cells. b Left, map of pLKO.1-sh-SNHG16-puro, a total of 7.4 kb in length. Right, SNHG16 levels in sh-SNHG16‑transfected MLE-12 cells. Values are mean ± SD. Results in Fig. S1a were representative of 3 independent experiments, and in Fig. S1b were representative of 2 independent experiments with similar findings. Fig. S2. Expression of SNHG16 in purified neutrophils and sorted T cells, monocytes and B cells from PB of healthy individuals. a Flow cytometric graphs of sorted CD3-positive T cells, CD14-positive monocytes and CD19-positive B cells from healthy individual No. 1, No. 2 and No. 3. b PB cell numbers/µL (left) and SNHG16 levels/105 cells (right) in neutrophils, T cells, monocytes and B cells from healthy individual No. 1, No. 2 and No. 3. Values are mean ± SD. Fig. S3. Expression of miR-146a in PBMCs and PBNs from SLE patients. a MiR-146a levels in PBMCs from SLE patients and HC. b A negative correlation between miR-146a levels in PBMCs and SLEDAI-2K activity scores. c MiR-146a levels in PBMCs from HC, Nil. LN, SLE-AH and other AH patients. A negative correlation between miR-146a and d TLR4, e TRAF6 and f NEAT1 levels in PBMCs from SLE patients. g MiR-146a levels in PBNs from SLE patients and HC. h A negative correlation between miR-146a levels in PBNs and SLEDAI-2K activity scores. i MiR-146a levels in PBNs from HC, Nil. LN and AH patients. Values are mean ± SD. Horizontal lines are mean values. Patient numbers, n = 62 for PBMCs from SLE, 15 for PBNs from SLE, 7 for PBMCs from Nil, LN and SLE-AH, 6 for PBMCs from other AH, 5 for PBNs from Nil, LN, 4 for PBN from AH. *p < 0.05. **p < 0.01, ***p < 0.001. Fig. S4. Expression of NEAT1 in SLE patients and pristane-injected mice. NEAT1 levels in a PBMCs and e PBNs from SLE patients and HC. b A positive c [file 12929_2023_969_MOESM1_ESM.zip › New Revised Suppl Fig 7.tif]

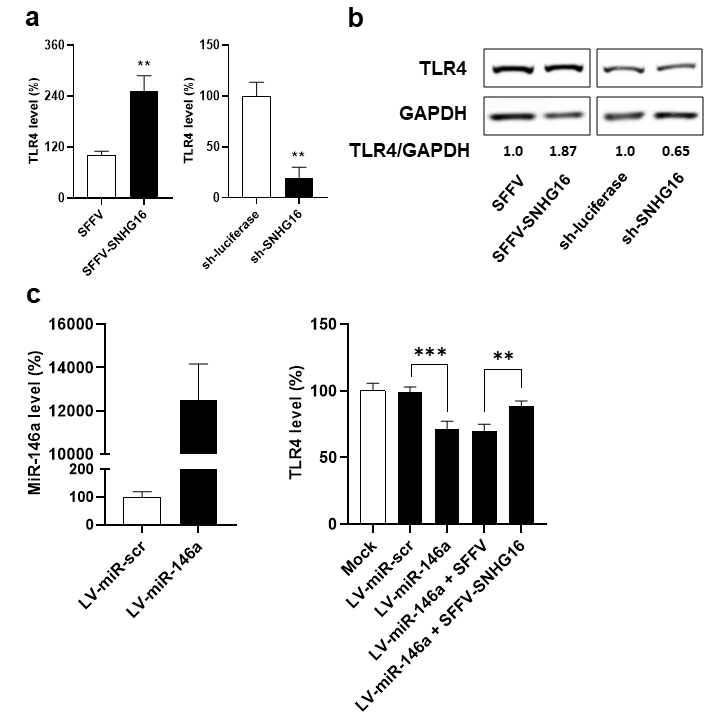

Supplement: Supplementary file 1 — Additional file 1: Fig. S1. SNHG16 expression in LV-SNHG16‑ and sh-SNHG16-transfected cells. a Left, map of pLV-SFFV-SNHG16-PGK-puro, a total of 9.3 kb in length. Right, SNHG16 levels in LV-SNHG16‑transfected 293T cells. b Left, map of pLKO.1-sh-SNHG16-puro, a total of 7.4 kb in length. Right, SNHG16 levels in sh-SNHG16‑transfected MLE-12 cells. Values are mean ± SD. Results in Fig. S1a were representative of 3 independent experiments, and in Fig. S1b were representative of 2 independent experiments with similar findings. Fig. S2. Expression of SNHG16 in purified neutrophils and sorted T cells, monocytes and B cells from PB of healthy individuals. a Flow cytometric graphs of sorted CD3-positive T cells, CD14-positive monocytes and CD19-positive B cells from healthy individual No. 1, No. 2 and No. 3. b PB cell numbers/µL (left) and SNHG16 levels/105 cells (right) in neutrophils, T cells, monocytes and B cells from healthy individual No. 1, No. 2 and No. 3. Values are mean ± SD. Fig. S3. Expression of miR-146a in PBMCs and PBNs from SLE patients. a MiR-146a levels in PBMCs from SLE patients and HC. b A negative correlation between miR-146a levels in PBMCs and SLEDAI-2K activity scores. c MiR-146a levels in PBMCs from HC, Nil. LN, SLE-AH and other AH patients. A negative correlation between miR-146a and d TLR4, e TRAF6 and f NEAT1 levels in PBMCs from SLE patients. g MiR-146a levels in PBNs from SLE patients and HC. h A negative correlation between miR-146a levels in PBNs and SLEDAI-2K activity scores. i MiR-146a levels in PBNs from HC, Nil. LN and AH patients. Values are mean ± SD. Horizontal lines are mean values. Patient numbers, n = 62 for PBMCs from SLE, 15 for PBNs from SLE, 7 for PBMCs from Nil, LN and SLE-AH, 6 for PBMCs from other AH, 5 for PBNs from Nil, LN, 4 for PBN from AH. *p < 0.05. **p < 0.01, ***p < 0.001. Fig. S4. Expression of NEAT1 in SLE patients and pristane-injected mice. NEAT1 levels in a PBMCs and e PBNs from SLE patients and HC. b A positive c [file 12929_2023_969_MOESM1_ESM.zip › New Revised Suppl Fig 8.tif]

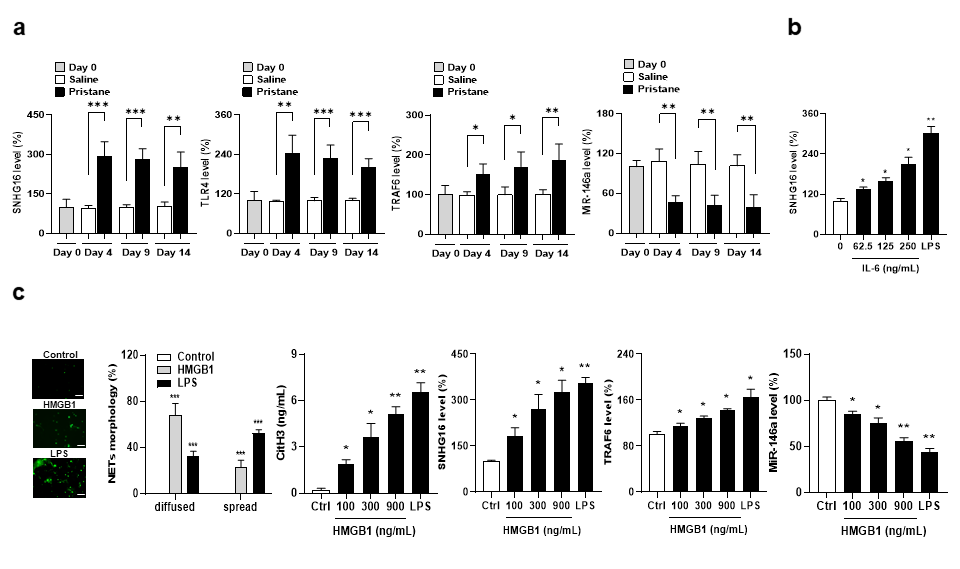

Supplement: Supplementary file 1 — Additional file 1: Fig. S1. SNHG16 expression in LV-SNHG16‑ and sh-SNHG16-transfected cells. a Left, map of pLV-SFFV-SNHG16-PGK-puro, a total of 9.3 kb in length. Right, SNHG16 levels in LV-SNHG16‑transfected 293T cells. b Left, map of pLKO.1-sh-SNHG16-puro, a total of 7.4 kb in length. Right, SNHG16 levels in sh-SNHG16‑transfected MLE-12 cells. Values are mean ± SD. Results in Fig. S1a were representative of 3 independent experiments, and in Fig. S1b were representative of 2 independent experiments with similar findings. Fig. S2. Expression of SNHG16 in purified neutrophils and sorted T cells, monocytes and B cells from PB of healthy individuals. a Flow cytometric graphs of sorted CD3-positive T cells, CD14-positive monocytes and CD19-positive B cells from healthy individual No. 1, No. 2 and No. 3. b PB cell numbers/µL (left) and SNHG16 levels/105 cells (right) in neutrophils, T cells, monocytes and B cells from healthy individual No. 1, No. 2 and No. 3. Values are mean ± SD. Fig. S3. Expression of miR-146a in PBMCs and PBNs from SLE patients. a MiR-146a levels in PBMCs from SLE patients and HC. b A negative correlation between miR-146a levels in PBMCs and SLEDAI-2K activity scores. c MiR-146a levels in PBMCs from HC, Nil. LN, SLE-AH and other AH patients. A negative correlation between miR-146a and d TLR4, e TRAF6 and f NEAT1 levels in PBMCs from SLE patients. g MiR-146a levels in PBNs from SLE patients and HC. h A negative correlation between miR-146a levels in PBNs and SLEDAI-2K activity scores. i MiR-146a levels in PBNs from HC, Nil. LN and AH patients. Values are mean ± SD. Horizontal lines are mean values. Patient numbers, n = 62 for PBMCs from SLE, 15 for PBNs from SLE, 7 for PBMCs from Nil, LN and SLE-AH, 6 for PBMCs from other AH, 5 for PBNs from Nil, LN, 4 for PBN from AH. *p < 0.05. **p < 0.01, ***p < 0.001. Fig. S4. Expression of NEAT1 in SLE patients and pristane-injected mice. NEAT1 levels in a PBMCs and e PBNs from SLE patients and HC. b A positive c [file 12929_2023_969_MOESM1_ESM.zip › New Revised Suppl Fig 9.tif]

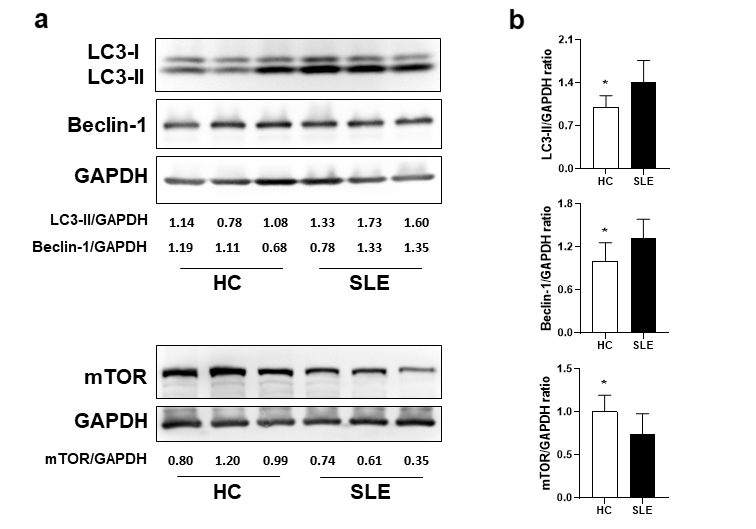

Supplement: Supplementary file 1 — Additional file 1: Fig. S1. SNHG16 expression in LV-SNHG16‑ and sh-SNHG16-transfected cells. a Left, map of pLV-SFFV-SNHG16-PGK-puro, a total of 9.3 kb in length. Right, SNHG16 levels in LV-SNHG16‑transfected 293T cells. b Left, map of pLKO.1-sh-SNHG16-puro, a total of 7.4 kb in length. Right, SNHG16 levels in sh-SNHG16‑transfected MLE-12 cells. Values are mean ± SD. Results in Fig. S1a were representative of 3 independent experiments, and in Fig. S1b were representative of 2 independent experiments with similar findings. Fig. S2. Expression of SNHG16 in purified neutrophils and sorted T cells, monocytes and B cells from PB of healthy individuals. a Flow cytometric graphs of sorted CD3-positive T cells, CD14-positive monocytes and CD19-positive B cells from healthy individual No. 1, No. 2 and No. 3. b PB cell numbers/µL (left) and SNHG16 levels/105 cells (right) in neutrophils, T cells, monocytes and B cells from healthy individual No. 1, No. 2 and No. 3. Values are mean ± SD. Fig. S3. Expression of miR-146a in PBMCs and PBNs from SLE patients. a MiR-146a levels in PBMCs from SLE patients and HC. b A negative correlation between miR-146a levels in PBMCs and SLEDAI-2K activity scores. c MiR-146a levels in PBMCs from HC, Nil. LN, SLE-AH and other AH patients. A negative correlation between miR-146a and d TLR4, e TRAF6 and f NEAT1 levels in PBMCs from SLE patients. g MiR-146a levels in PBNs from SLE patients and HC. h A negative correlation between miR-146a levels in PBNs and SLEDAI-2K activity scores. i MiR-146a levels in PBNs from HC, Nil. LN and AH patients. Values are mean ± SD. Horizontal lines are mean values. Patient numbers, n = 62 for PBMCs from SLE, 15 for PBNs from SLE, 7 for PBMCs from Nil, LN and SLE-AH, 6 for PBMCs from other AH, 5 for PBNs from Nil, LN, 4 for PBN from AH. *p < 0.05. **p < 0.01, ***p < 0.001. Fig. S4. Expression of NEAT1 in SLE patients and pristane-injected mice. NEAT1 levels in a PBMCs and e PBNs from SLE patients and HC. b A positive c [file 12929_2023_969_MOESM1_ESM.zip › Revised Suppl Fig 6.tif]
