# Supplementary material for: Long noncoding RNA SNHG16 regulates TLR4-mediated autophagy and NETosis formation in alveolar hemorrhage associated with systemic lupus erythematosus
Source: J Biomed Sci. 2023 Sep 12;30:78. doi: 10.1186/s12929-023-00969-5 (PMC10496234; doi:10.1186/s12929-023-00969-5)
Supplement: Supplementary file 2 — Additional file 2: Table S1. Clinical profiles and therapeutic modality of AH in different patient groups. Table S2. Linear regression for SNHG16, TLR4 and TRAF6 levels in PBMCs from SLE and HC. Table S3. Linear regression for SNHG16, TLR4 and TRAF6 levels in PBNs from SLE and HC. [file 12929_2023_969_MOESM2_ESM.docx]

**Additional file 2**

**Table S1** Clinical profiles and therapeutic modality of AH in different patient groups

| **Patient group** | SLE | AAV | APS | IgAV |
| --- | --- | --- | --- | --- |
| No. | 7 | 3 | 2 | 1 |
| Age (year)  Mean + SD | 21~58  37 + 14 | 53~82  65 + 15 | 27, 35  31 + 6 | 48 |
| Gender | 6 F, 1 M | 2 F, 1 M | 2 F | 1 M |
| **Clinical profile** |  |  |  |  |
| Disease period (years)  Mean + SD | 0~10  5.3 + 3.3 | 2~13  7.7 + 5.5 | 5~10  7.5 + 3.5 | 12 |
| *Activity  Mean + SD | 14~27  18.1 + 4.4 | 22~30  26.0 + 4.0 |  |  |
| Kidney involvement | 6 (86%) | 3 (100%) | 1 (50%) | 1 (100%) |
| Heart involvement | 2 (29%) | 2 (67%) | 1 (50%) | 0 |
| GI involvement | 1 (14%) | 1 (33%) | 0 | 1 (100%) |
| Involved organ No.  Mean + SD | 3~5  3.9 + 0.9 | 3 or 4  3.3 + 0.6 | 2 or 3  2.5 + 0.7 | 4 |
| ^@^Long-term survival | 6 (86%) | 2 (67%) | 1 (50%) | 1 (100%) |
| **Therapeutic modality** |  |  |  |  |
| Corticosteroid | 7 (100%) | 3 (100%) | 2 (100%) | 1 (100%) |
| Cyclophosphamide | 5 (71%) | 1 (33%) | 1 (50%) | 1 (100%) |
| Mycophenolate mofetil | 2 (29%) | 0 | 0 | 0 |
| Azathioprine | 6 (86%) | 3 (100%) | 2 (100%) | 1 (100%) |
| Rituximab | 2 (29%) | 3 (100%) | 1 (50%) | 0 |
| Plasmapheresis | 2 (29%) | 1 (33%) | 0 | 0 |
| Mechanic ventilator | 6 (86%) | 2 (67%) | 1 (50%) | 1 (100%) |

*SLEDAI-2K for SLE, BVAS for AAV, ^@^Death due to DAH-related respiratory failure in one SLE and one AAV patients; AAV: Anti-neutrophil cytoplasmic antibody-associated vasculitis, AH: alveolar hemorrhage, APS: anti-phospholipid syndrome, BVAS: Birmingham Vasculitis Activity Score, F: female, GI: gastrointestinal, IgAV: IgA vasculitis, M: male. No.: number, SD: standard deviation, SLE: systemic lupus erythematosus, SLEDAI-2K: SLE disease activity index 2000

**Table S2** Linear regression for SNHG16, TLR4 and TRAF6 levels in PBMCs from SLE and HC

| **Item** | **SLE versus HC** | | | |
| --- | --- | --- | --- | --- |
|  | Univariate analysis | | Multivariable analysis^a^ | |
| Outcome^c^ | β + SE | *p* value | β + SE | *p* value |
| SNHG16 | 1.24 + 0.54 | 0.025 | 1.24 + 0.54 | 0.025 |
| TLR4 | 0.66 + 0.17 | <0.001 | 0.66 + 0.17 | <0.001 |
| TRAF6 | 0.42 + 0.14 | 0.003 | 0.41 + 0.14 | 0.004 |
| **Item** | **SLEDAI-2k scores** | | | |
|  | Univariate analysis | | Multivariable analysis^b^ | |
| Outcome^c^ | β + SE | *p* value | β + SE | *p* value |
| SNHG16 | 0.16 + 0.05 | 0.005 | 0.19 + 0.06 | 0.002 |
| TLR4 | 0.05 + 0.02 | 0.011 | 0.07 + 0.02 | <0.001 |
| TRAF6 | 0.04 + 0.01 | 0.005 | 0.05 + 0.01 | 0.001 |

^a^Adjustments made for age and sex.

^b^Adjustments made for age, sex, and medications (corticosteroid, hydroxychloroquine and mycophenolate mofetil)

^c^Logarithmic transformation of the outcome variable

HC: healthy control, PBMC: peripheral blood mononuclear cell, SE: standard error, SLE: systemic lupus erythematosus, SLEDAI-2k: SLE disease activity 2000

**Table S3** Linear regression for SNHG16, TLR4 and TRAF6 levels in PBNs from SLE and HC

| **Item** | **SLE versus HC** | | | |
| --- | --- | --- | --- | --- |
|  | Univariate analysis | | Multivariable analysis^a^ | |
| Outcome^c^ | β + SE | *p* value | β + SE | *p* value |
| SNHG16 | 0.48 + 0.19 | 0.018 | 0.48 + 0.19 | 0.020 |
| TLR4 | 0.81 + 0.19 | <0.001 | 0.81 + 0.19 | <0.001 |
| TRAF6 | 0.48 + 0.18 | 0.015 | 0.48 + 0.19 | 0.016 |
| **Item** | **SLEDAI-2k scores** | | | |
|  | Univariate analysis | | Multivariable analysis^b^ | |
| Outcome^c^ | β + SE | *p* value | β + SE | *p* value |
| SNHG16 | 0.04 + 0.01 | 0.006 | 0.04 + 0.01 | 0.008 |
| TLR4 | 0.05 + 0.01 | 0.005 | 0.05 + 0.01 | 0.007 |
| TRAF6 | 0.04 + 0.01 | 0.002 | 0.04 + 0.01 | 0.001 |

^a^Adjustments made for age and sex.

^b^Adjustments made for age, sex, and medications (corticosteroid, hydroxychloroquine and mycophenolate mofetil)

^c^Logarithmic transformation of the outcome variable

HC: healthy control, PBN: peripheral blood neutrophil, SE: standard error, SLE: systemic lupus erythematosus, SLEDAI-2k: SLE disease activity 2000
